# Towards a brain‐based predictome of mental illness

**DOI:** 10.1002/hbm.25013

**Published:** 2020-05-06

**Authors:** Barnaly Rashid, Vince Calhoun

**Affiliations:** ^1^ Department of Psychiatry Harvard Medical School Boston Massachusetts USA; ^2^ Tri‐Institutional Center for Translational Research in Neuroimaging and Data Science (TReNDS) Georgia State University, Georgia Institute of Technology, and Emory University Atlanta Georgia USA

**Keywords:** functional magnetic resonance imaging, machine learning, multimodal studies, neuroimaging, psychiatric disorder

## Abstract

Neuroimaging‐based approaches have been extensively applied to study mental illness in recent years and have deepened our understanding of both cognitively healthy and disordered brain structure and function. Recent advancements in machine learning techniques have shown promising outcomes for individualized prediction and characterization of patients with psychiatric disorders. Studies have utilized features from a variety of neuroimaging modalities, including structural, functional, and diffusion magnetic resonance imaging data, as well as jointly estimated features from multiple modalities, to assess patients with heterogeneous mental disorders, such as schizophrenia and autism. We use the term “predictome” to describe the use of multivariate brain network features from one or more neuroimaging modalities to predict mental illness. In the predictome, multiple brain network‐based features (either from the same modality or multiple modalities) are incorporated into a predictive model to jointly estimate features that are unique to a disorder and predict subjects accordingly. To date, more than 650 studies have been published on subject‐level prediction focusing on psychiatric disorders. We have surveyed about 250 studies including schizophrenia, major depression, bipolar disorder, autism spectrum disorder, attention‐deficit hyperactivity disorder, obsessive–compulsive disorder, social anxiety disorder, posttraumatic stress disorder, and substance dependence. In this review, we present a comprehensive review of recent neuroimaging‐based predictomic approaches, current trends, and common shortcomings and share our vision for future directions.

**Abbreviations**
AAL: automated anatomical labeling
ACC: anterior cingulate cortex
ADHD: attention‐deficit hyperactive disorder
ANN: artificial neural network
AOD: auditory oddball
ASD: autism spectrum disorder
AUC: area under curve
AUD: alcohol use disorder
AZ‐CPT: AX version of continuous performance task
BOLD: blood‐oxygen level dependent
BP: bipolar disorder
BPd: bipolar disorder depressed state
BPr: bipolar disorder remitted state
cMDD: current MDD
CNN: convolutional neural network
Cohe‐ReHo: coherence regional homogeneity
CSF: cerebrospinal fluid
CT: cortical thickness
DBM: deformation‐based morphometry
DFA: discriminant function analysis
DLPFC: dorsolateral prefrontal cortex
DMN: default mode network
dMRI: diffusion MRI
DNN: deep neural network
DTI: diffusion tensor imaging
EC: effective connectivity
ED: Euclidean distance
EMD: earth mover's distance
Enet‐TV: ElasticNet‐total variation
FA: fractional anisotropy
fALEF: fractional amplitude of low frequency fluctuations
FC: functional connectivity
FFT: fast Fourier transform
FNC: functional network connectivity
GAD: generalized anxiety disorder
GBDT: Gradient Boosting Decision Tree
gLASSO: group least absolute shrinkage and selection operator
GLM: general linear modeling
GM: gray matter
GMD: gray matter density
GMV: gray matter volume
GNB: Gaussian Naïve Bayes
GPC: Gaussian process classifier
HC: healthy control
H‐ELM: extreme learning machine
HOG‐MHPC: fMRI histogram of oriented gradients‐feature‐based patient classification
ICA: independent component analysis
ITG: inferior temporal gyrus
KDA: kernel discriminant analysis
KNN: k‐nearest neighbor
LDA: linear discriminant analysis
LDC: Linear discriminant classifier
LR: logistic regression
LRC: logistic regression classifier
MD: mean diffusivity
MD: mood disorder
MDD: major depression disorder
MDDd: major depressive disorder depressed state
MDDr: major depressive disorder remitted state
MFMK‐SVM: multi‐kernel SVM learning method
MLP: multi‐layer perceptron
MMC: maximum margin clustering
mMLDA: modified maximum uncertainty linear discriminant analysis
MRS: magnetic resonance spectroscopy
MVPA: multivoxel pattern analysis
n.s.: not specified
NMF: non‐negative matrix factorization
NN: neural network
OCD: obsessive–compulsive disorder
PCA: principle component analysis
PLS: partial least squares regression
PTSD: posttraumatic stress disorder
RAVENS: regional analysis of brain volumes in normalized space
ReHo: regional homogeneity
RF: random forest
rMDD: remitted MDD
ROI: region of interest
rsfMRI: resting‐state fMRI
RSN: resting‐state network
RVM: relevance vector machine
SAD: social anxiety disorder
SAEs: sparse autoencoders
SD: substance dependence
SIFT: scale‐invariant feature transform
SM: sensorimotor
sMRI: structural MRI
SNP: single nucleotide polymorphism
SVM: support vector machine
SVM‐RFE: support vector machine with recursive feature elimination
SZ: schizophrenia
SZA: schizoaffective
TD: typically developing
TDC: typically developing children
TRD: treatment‐resistant depression
TSD: treatment‐sensitive depression
VBM: voxel‐based morphometry
VMHC: voxel‐mirrored homotopic correlations
WBM: wavelet‐based morphometry
WM: white matter
WMT: working memory task
WMV: white matter volume

## 1 INTRODUCTION

With the first steps toward establishing modern psychiatry, and the first attempts to classify different mental phenomena and disorders into disease‐specific categories, the need for diagnostic tools to allow for an objective evaluation of mental illness has surfaced. Current clinical diagnosis, disease evaluation and treatment plans for severe psychiatric disorders are solely based on cross‐sectional self‐reported clinical symptoms supported by information on longitudinal course and outcome. Researchers have been actively searching for objective, biologically based, disease indicators, or “biomarkers,” and after several decades of experiments and attempts of classifying psychiatric disorders based on predefined symptom categories, we are currently at a turning point where a new paradigm has emerged: The Research Domain Criteria (RDoC; T. Insel et al., 2010). This approach aims at incorporating the most recent findings from clinical and genetic neuroscience, thereby opening the field to a dimensional approach informed by the specific neural pathophysiology underlying psychiatric disorders. By utilizing advanced neuroimaging techniques, it is now possible to study disease‐specific structural and functional brain impairments. Neuroimaging modalities, such as magnetic resonance imaging (MRI), magnetoencephalography (MEG), and electroencephalography (EEG) offer tools to noninvasively study the neural structure of psychiatric disorders with exceptional accuracy. Using these powerful techniques, researchers have begun to understand the complex neural function and structure that may lead to specific disorders.

In recent years, there has been a growing trend in designing neuroimaging‐based prognostic/diagnostic tools. As a result, there has been a lot of effort focused on the use of neuroimaging tools to automatically discriminate patients with brain disorders from healthy control (HC) or each other. Many of these studies have reported promising prediction performances with the claim that complex mental illness can be diagnosed robustly, accurately and rapidly in an automatic fashion. However, until now, these tools have not been integrated into the clinical realm. We believe a key reason for this is that many of the studies of this nature, despite the promising results on a specific research dataset, are not designed to generalize to other datasets, specifically the clinical ones.

In this systematic review, we surveyed the existing literature on the application of machine learning‐based techniques for diagnostic‐focused predictive analyses in psychiatric research and discuss current trends and future directions. While previous reviews have focused on a specific machine learning technique (Orru, Pettersson‐Yeo, Marquand, Sartori, & Mechelli, 2012), a single disorder (e.g., schizophrenia [SZ], Calhoun & Arbabshirani, 2013; Kambeitz et al., 2015), major depression disorder (MDD; Gao, Calhoun, & Sui, 2018), and autism spectrum disorder (Retico, Tosetti, Muratori, & Calderoni, 2014)), a single imaging modality (B. Sundermann, Herr, Schwindt, & Pfleiderer, 2014), a small subset of disorders (Klöppel et al., 2012), or general brain‐based disorders (Arbabshirani, Plis, Sui, & Calhoun, 2017), we aim to provide a comprehensive review of all major psychiatric disorders. So far, the most extensive review on major psychiatric disorders is the review article by Wolfers et al., where about 120 pattern recognition studies in SZ, mood disorders, attention‐deficit hyperactivity disorder (ADHD), autism spectrum disorder (ASD), anxiety disorders, and specific phobias have been reviewed (Wolfers, Buitelaar, Beckmann, Franke, & Marquand, 2015). While there are some overlaps among the aforementioned studies and this current survey, to the best of our knowledge, this is by far the largest survey in the field of major psychiatric disorders based on the number of papers reviewed (about 250 papers). Further, in recent years, there has been an exponential growth of predictive analysis studies, and therefore, an updated survey is much warranted.

In this review, a general discussion of the current trends in the brain‐based psychiatric “predictome” and their translational perspectives will be provided, along with highlighting some of the common challenges and guidelines for future directions. We also discuss emerging trends in neuroimaging such as data sharing, multimodal brain imaging, and differential diagnosis. The main goals of this study include: (a) to review and systematically compare a large number of recent MRI‐based mental disorder diagnostic/prognostic studies in SZ, MDD, bipolar (BP) disorder, ASD, ADHD, obsessive–compulsive disorder (OCD), social anxiety disorder (SAD), posttraumatic stress disorder (PTSD) and substance dependence (SD), and (b) to discuss pitfalls and promises of existing machine learning techniques, and (c) to provide our vision and future directions to address some of the challenges. While there are a number of challenges remain to be addressed, brain‐based predictome studies have made a considerable progress in recent years. We hope that, with more sophisticated machine learning approaches integrated with large‐scale data, predictive modeling tools will transition from the “proof‐of‐concept” stage to the “ready for clinical implementation” stage in the near future.

## 2 DEVELOPING A MENTAL ILLNESS PREDICTOME PIPELINE

**FIGURE 1:**
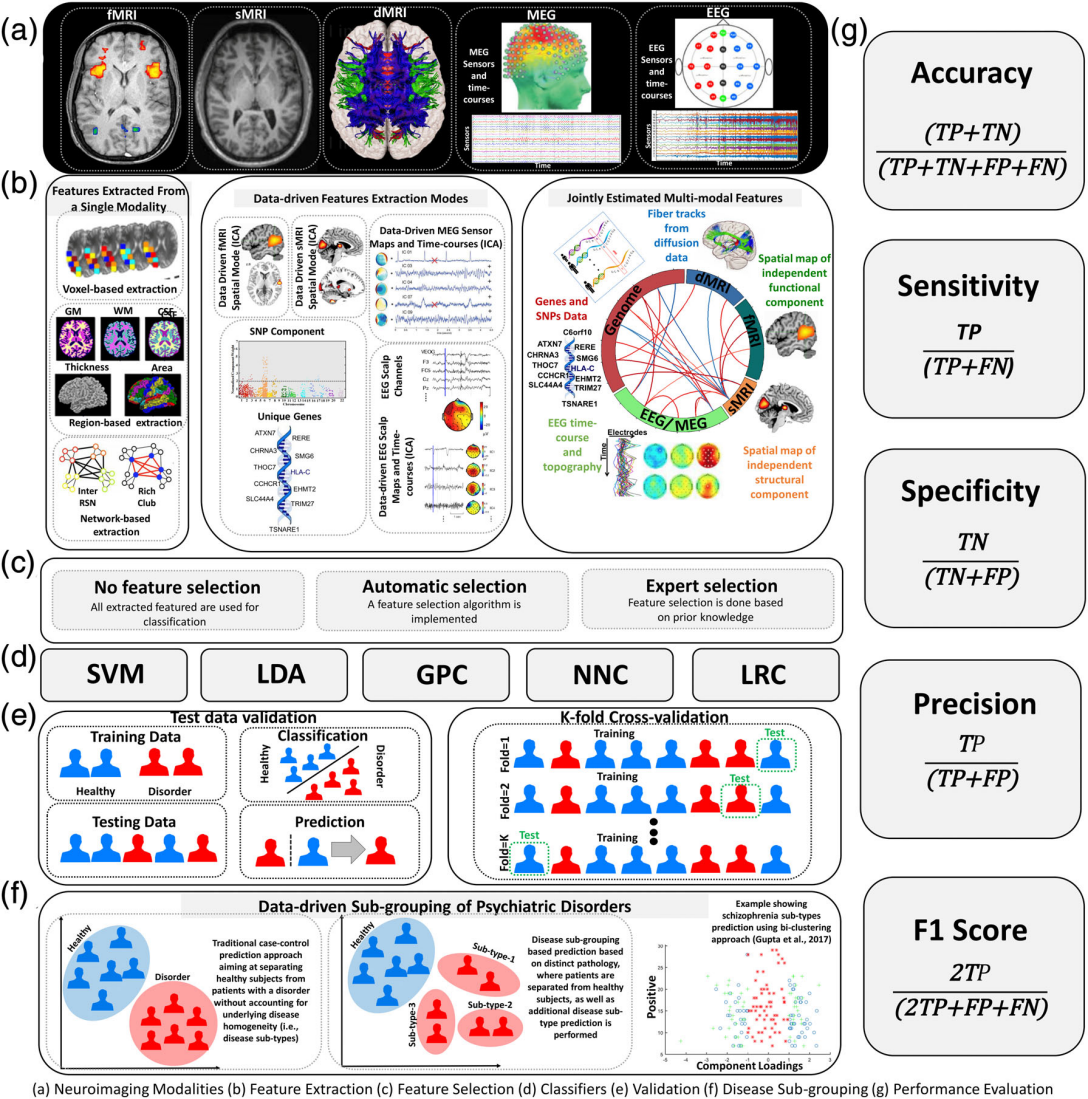
Predictome pipeline. An overview of neuroimaging‐based predictome pipeline. (a) Neuroimaging modalities typically used for mental illness prediction. (b) Current approaches for feature selection. Feature extraction can include (i) voxel‐based (ii) network‐based, (iii) data‐driven approaches (e.g., independent component analysis, ICA), or (iv) jointly estimated features from multiple modalities (e.g., fMRI and genomics). (c) Types of feature selections can include automatic or expert selection approaches. (d) Choice of classifiers may include support vector machine (SVM), linear discriminant analysis (LDA), Gaussian process classifier (GPC), neural network classifier (NNC) or logistic regression classifier (LRC). (e) Model validation can be performed using either a test‐validation setup or using a *k*‐fold cross‐validation scheme. (f) Data‐driven subtype identification can also be performed for homogeneous disorders (Gupta et al., 2017; Marquand, Rezek, Buitelaar, & Beckmann, 2016). (g) Various measures for performance evaluation such as accuracy, sensitivity, specificity, precision and F1‐score. FN, false negative; FP, false positive; TN, true negative; TP, true positive

Predictome studies using neuroimaging data aim to extract multivariate brain network features from one or more neuroimaging modalities to predict outcome measures such as specific psychiatric diagnoses. Typically, after feature extraction and selection, a classifier is trained in a supervised or semi‐supervised way with a predefined set of labels. Further model validation is performed either using an independent testing dataset or by incorporating a cross‐validation (CV) scheme. Figure 1 presents the most common components of a brain‐based predictome pipeline of mental illness prediction using neuroimaging data. While specific pipelines might vary at different preprocessing and postprocessing stages, conventional predictome analyses typically include the following steps: (a) feature extraction and selection/reduction, (b) classifier training, (c) classification and CV, and (d) performance evaluation.

### 2.1 Feature extraction, selection/reduction

The first step of a predictome analysis is to transform neuroimaging data into features (i.e., deciding what to use as features and extracting these feature values from the data). A neuroimaging feature refers to any derived variable containing valuable information about the class labels that can be extracted from the data.

In this survey, we reviewed and highlighted predictome studies based on the type of features used for classification purposes, including voxel‐based, region‐based, and brain‐network based feature selection approaches. For example, features can simply be a set of brain voxels within a particular brain network, or a region of interest (ROI), multivariate data‐driven (e.g., using independent component analysis [ICA]) brain networks, or jointly estimated multimodal features, as seen in Figure 1b. A voxel‐based approach employs feature extraction at the brain voxel level, while a region‐based approach identifies and extracts predefined region‐of‐interests (ROIs) as features based on a brain atlas (either functional or structural). A network‐based feature extraction approach, such as ICA, aims at combining multiple voxels across brain network (Calhoun, Adali, Pearlson, & Pekar, 2001b; McKeown et al., 1998).

In addition to feature extraction, it is often important to reduce the number of features from high‐dimensional neuroimaging data before proceeding with model training. In the context of neuroimaging, feature selection can help achieve higher accuracy rates (Ad‐Dab'bagh et al., 2006), and allow a more specific focus on the underlying brain regions that account for between‐group differences (Plitt, Barnes, & Martin, 2015). Indeed, the number of features in neuroimaging data is large with many irrelevant features not contributing to the prediction power of the model, and not all disorders affect every brain network in the same way. Thus, some brain‐based features might not contribute to the diagnosis labels, and some features may capture redundant information already uncovered by other features. Computational time and model generalization can also be improved by excluding redundant and unrelated features (Dash & Liu, 1997; Guyon & Elisseeff, 2003; Moradi et al., 2015). Feature selection approaches (e.g., principle component analysis [PCA]) project the high‐dimensional neuroimaging data into a lower dimensional space with a goal of preserving model discriminative power. Although not an essential step, in order to improve the strength of the prediction algorithm, it is important to select both optimum and meaningful features (Chu et al., 2012; Cuingnet et al., 2011). In a supervised learning approach, most discriminative features are selected to amplify the signal and reduce the noise. Often, prior information is used to address the dimensionality issue of neuroimaging data. Based on the characteristics of features and the type of learning problem, a particular feature selection approach is used (Mwangi, Tian, & Soares, 2014). Common feature selection approaches include: (a) expert feature selection (based on prior knowledge) and (b) automatic feature selection (based on a feature selection algorithm). A combination of these two approaches can also be used for feature selection. For example, an expert feature selection approach can first be implemented by selecting a previously known disorder‐specific ROI, and then an automatic feature selection algorithm can be used to favor discriminative features within the predefined ROI. Note that, to avoid performance bias, feature selection and extraction methods should be limited to training dataset.

### 2.2 Classifier training

A classifier is a function that takes features as input and generates a class label prediction. Based on the learning function and underlying assumptions, different types of classifiers can be developed. Neuroimaging studies have applied various classifiers for mental illness prediction. The dimensionality issue associated with the relatively large number of features and the small number of samples should be accounted for while applying such classification algorithms. Typically, the classifier learns a rule and separates the underlying classes optimally. Any type of classification or regression algorithm can be used for training purpose, such as linear and logistic regression algorithms, multilayer neural networks and Gaussian approaches (Bishop, 2006), in the current review, we have limited our focus on classifiers using discrete outcome measures (i.e., diagnostic labels), with the exception of discussion on translational perspective and advanced predictive modeling (Sections 5 and 6).

#### 2.2.1 Nearest‐neighbor

The simplest form of classifier is known as the “nearest‐neighbor” which does not require any explicit learning of a classification function. Using the *nearest‐neighbor* approach, classification of an independent test sample is performed by identifying the most similar measures, for example, lowest Euclidean distance, between the training and testing samples, and then assigning the label of the training sample (i.e., nearest neighbor) to the test sample.

#### 2.2.2 Discriminative and generative models

Other classifiers that require explicit learning function can be categorized as discriminative and generative models (Trevor, Robert, & Friedman, 2009). A discriminative classifier directly learns to predict from the training data using a learning function based on predefined parameters. In contrast, generative classifier learns a statistical model to generate class labels by modeling the distributions of feature values that are conditional on example class labels.

#### 2.2.3 Support vector machine

During the training stage of a supervised learning, data labels are used to optimize the model by finding a *hyperplane* or *decision boundary* that can maximally discriminate between groups. The most common choice for a simple learning function is predicting class labels based on a linear combination of the features that might influence the outcomes. A linear classifier can be viewed as learning a line or boundary (i.e., decision boundary) that separates points within the two classes and discriminates their labels. For instance, a linear support vector machine (SVM) is such a classifier that learns the decision boundary. Due to its widespread use and promising results in neuroimaging‐based prediction, SVM is the most commonly seen classifier in our current survey. The SVM algorithm is typically intended for binary classification that aims at maximizing the boundary between different classes in a higher dimensional space. Mathematically, the discriminant function for SVM consists of a weight vector orthogonal to the decision boundary, and is specified by the data points that lie closest to the decision boundary, known as support vectors. This decision boundary further defines the classification rule of new, unseen cases.

#### 2.2.4 Linear discriminant classifier

Another powerful linear model is the linear discriminant classifier (LDC) that attempts to separate classes by maximizing the variance of between‐class to within‐class ratio. An example of probabilistic discriminant model is the logistic regression classifier (LRC) that focuses on learning an optimum decision rule by modeling the log‐odds ratio as a linear combination of predictor variables (i.e., feature). Both LDC and LRC methods yield probabilistic predictions that a new case can be assigned to a particular class and a class label.

#### 2.2.5 Gaussian process classifier

Further, a Gaussian process classifier (GPC) is a probabilistic model and is a Bayesian extension of LRC (Wolfers et al., 2015). Briefly, GPC is first trained using the training feature to determine an optimized predictive distribution distinguishing between case and control. Note that, parameters relevant to this predictive distribution are estimated by maximizing the logarithm of the marginal likelihood on the training features. During the training stage, GPC then predicts the case and control by providing the predictive distribution of the test data using a sigmoid function (Frangou, Dima, & Jogia, 2017). For technical details of GPC, refer to Schrouff et al. (2013).

#### 2.2.6 Neural network classifier

Also, artificial neural network classifiers (NNC) have recently become popular for modeling biological networks. Multilayer NNC is the extension of linear perceptron classifier, which can yield complex nonlinear decision boundaries. Typically, the structure of NNC includes an input layer, hidden layer(s) and an output layer. Neurons from each of these layers are connected to the neurons of the subsequent layers. A variety of nonlinear transfer functions of the hidden later neurons can be used (e.g., sigmoid function). Briefly, during the training phase, the weights across a set of artificial connected neurons are adjusted for learning purposes using backpropagation technique (Werbos, 1990), and then used for classification. For example, in case of mental illness prediction, artificial NNC analyzes the training labels (i.e., healthy versus disorder) and learns to identify a test example.

#### 2.2.7 Random forest

Other recent and more powerful approaches for brain‐based prediction include random forest and deep learning classifiers. In the random forest classifier, which is an ensemble of decision tree classifiers, multiple levels of randomization are integrated (Breiman, 2001). Using a randomized subset of the training data, each decision tree is grown, and each node is subsequently formed by searching through a random subset of training features. For each feature, the classifier estimates a score to highlight the feature's discriminative power (i.e., Gini Importance [GI] score). The random forest approach offers improved generalization accuracy as it randomizes training subjects, particularly in cases with a relatively smaller training subjects compared to the number of training features. Further, the random forest classifier provides nonlinear decision boundaries, which helps to model nonlinear patters of features during training.

#### 2.2.8 Deep learning

Deep learning classifiers have recently become an attractive choice for mental illness prediction (Calhoun & Sui, 2016; Han, Huang, Zhang, Zhao, & Chen, 2017; Iidaka, 2015; Jang, Plis, Calhoun, & Lee, 2017; J. Kim, Calhoun, Shim, & Lee, 2016; Plis et al., 2014). Deep learning classifiers can learn the features with optimal discriminating power directly from the raw data by using a hierarchical approach (Schmidhuber, 2015; Vieira, Pinaya, & Mechelli, 2017). This provides a great advantage over conventional classifiers that require explicit feature reduction steps. By applying nonlinear transformations to the raw data, deep learning classifiers automatically overcome issues with feature selection which is particularly helpful for higher‐dimensional features or data with lack of prior knowledge.

### 2.3 Classification and CV framework

Once the classifier learns the decision rule based on features from a training set, the next step is to validate the model in a testing set. In order to mitigate performance bias and overfitting from predictive modeling, it is critical to analyze the training and testing datasets independently. During the training stage, the classifier learns to predict the labels from the training features based on the associated learning algorithm. For example, for learning problems without complex, iterative feature selection, the trained classifier is tested on previously unseen testing data (Wolfers et al., 2015). In order to achieve better model performance, a classifier should be trained with as much training data as possible, which is often a challenging issue in neuroimaging‐based prediction studies. CV approach offers us to train classifiers with a higher number of training samples. A common CV approach is to repeatedly evaluate model performance using multiple training and testing partitions, a validation approach known as *k*‐fold CV (*k*: number of data partitions; Kohavi, 1995; Patel, Khalaf, & Aizenstein, 2016). Other popular CV approaches include, leave‐one‐out (LOO‐CV), and holdout. LOO‐CV is an iterative process, typically used on smaller sample size, where *k* is equal to the number of samples and every subject in the whole sample is left out once for testing the classifier. Briefly, the LOO‐CV procedure includes the following steps: (a) leave one sample out, train on the remaining ones, make a prediction for this sample (b) repeat for each sample in turn, and (c) compute the accuracy of the predictions made for all the samples. While a popular choice, leaving each sample out could become computationally expensive as it requires training of as many classifiers as the number of samples. In addition, LOO‐CV has also been shown to potentially introduce some prediction bias (Varoquaux et al., 2017), as it could introduce high variance by providing more data during the training state, which could also result in overfitting (Elisseeff & Pontil, 2003; Refaeilzadeh, Tang, & Liu, 2009). Because of this, the preferred approach is k‐fold CV where *k* < number of samples. Common choices for partitioning are *k* = 10 or *k* = 5, corresponding to leaving out 10 or 20% of the total samples during each validation fold. Other important considerations for designing a CV procedure include: (a) inclusion of examples from all classes in the training data for better prediction accuracy, (b) having roughly equal number of samples across classes (i.e., balanced classes), and (c) inclusion of correlated samples in the same fold to avoid misleading performance that may accurately predict test samples with a correlated counterpart in the training set (Pereira, Mitchell, & Botvinick, 2009).

Performance measures, including accuracy, are averaged across iterations for the training and testing phase. For a supervised approach, a model is optimized using labeled data to find a discriminative decision boundary or *hyperplane* differentiating between case and control groups. The model parameters are optimized for maximum discrimination between groups. The CV approach helps ensure generalization of the training. During the classification stage, the trained model is then used to predict the label for new, unseen observations from testing set. For an unbiased generalization, it is important that the testing data do not overlap with the training data (Lemm, Blankertz, Dickhaus, & Müller, 2011). Further, the new data should be preprocessed in the same way as the training data.

More recently, another type of CV has been introduced, where various types of classifiers are cross‐validated by running multiple classifiers on the same training data. For example, “Polyssifier” can be used to cross‐validate multiple classifiers, where the baseline is first computed by applying multiple classifiers, such as nearest neighbors, linear SVM, radial basis function or RBF‐SVM, decision tree, random forest, logistic regression, naive Bayes, and linear discriminative analysis (LDA; http://mialab.mrn.org/software/#polyssifier).

### 2.4 Measures for performance evaluation

The most commonly used performance evaluation measures for predictive algorithms include accuracy, sensitivity, specificity and the receiver operating characteristic (ROC) curve. These measures provide an evaluation of how accurately a classifier can generalize to new test samples (i.e., cases). In a clinical context, accuracy indicates how accurately the model classifies the cases and controls, sensitivity shows the proportion of true positives correctly identified (i.e., what percentage of cases are truly identified), and specificity demonstrates the proportion of true negatives correctly identified (i.e., what percentage of controls are truly identified) by the model. The overall performance of the model can be assessed by the ROC curve which provides a summary of the area under the curve (AUC). A high sensitivity suggests that only a few participants are falsely diagnosed as HCs while actually being patients, and a high specificity indicates that a few participants are falsely diagnosed as patients while actually being HCs. The accuracy refers to the total proportion of samples correctly classified. The ROC curves show the balance between the true positive rate (sensitivity) and the false positive rate (1‐specificity) across a range of decision thresholds within the model. To avoid bias by potential imbalances between groups, a common practice is to report balanced accuracy measures, by taking an average accuracy obtained for each diagnostic label (Brodersen, Ong, Stephan, & Buhmann, 2010). A useful measure to summarize the classification performance is to provide a confusion matrix, which represents actual labels on one side and the predicted labels on the other side. This is more important with models predicting more than two groups (Baldi, Brunak, Chauvin, Andersen, & Nielsen, 2000). Other useful performance measures can be extracted from the confusion matrix including positive predictive value (PPV), negative predictive value (NPV), F1‐score (harmonic mean of precision and recall), and G‐mean (geometric mean of precision and recall; Alberg, Park, Hager, Brock, & Diener‐West, 2004). Positive and negative predictive values are important for predictive studies as they directly quantify the potential utility of the classifier for clinical diagnosis. The positive predictive value is defined as the number times the classifier correctly predicted participants as patients (i.e., positive diagnosis) divided by the total number of positive predictions. The negative predictive value is defined as the number of times the classifier correctly predicted a negative diagnosis divided by the total number of negative predictions.

## 3 PREDICTION OF MENTAL ILLNESS USING NEUROIMAGING TECHNIQUES

With recent advancements in medical imaging technology, neuroimaging data is being collected more rapidly and at finer resolution than ever before. In recent years, there has been an increasing interest in leveraging this vast amount of brain data across analytic levels, acquisition approaches, and experimental designs to achieve a deeper understanding of brain structure and function. In this review, we use the term “predictome” to describe the use of multivariate brain network features from one or more neuroimaging modalities to predict mental illness. In the predictome, multiple brain network‐based features (either from the same modality or multiple modalities) are incorporated into a predictive model to jointly estimate features that are unique to a disorder and predict subjects accordingly. Here, we review recent predictomic approaches used for neuroimaging classification and prediction, and provide an overview of studies for prediction of mental illness from their healthy counterparts.

### 3.1 Survey procedure for the current literature review

**FIGURE 2:**
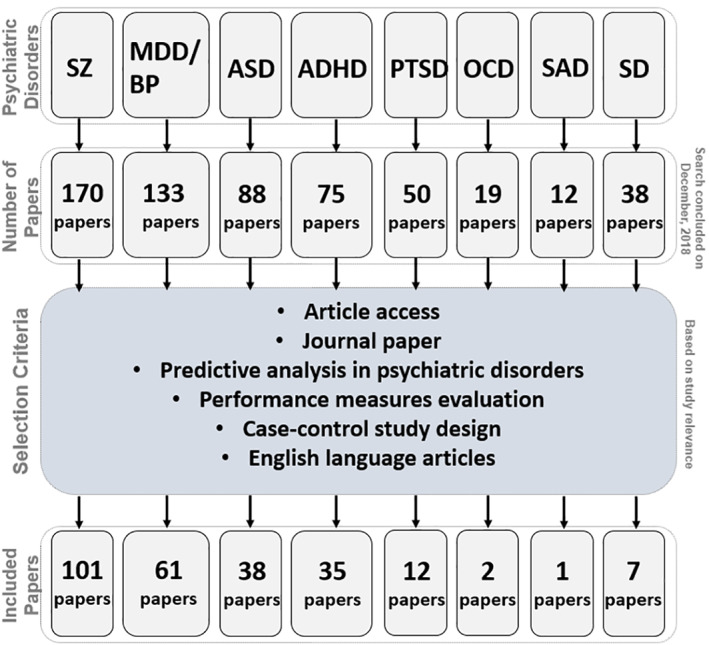
The systematic literature review procedure, the inclusion criteria and the number of surveyed studies for each modality. ADHD, attention‐deficit/hyperactivity disorder; ASD, autism spectrum disorder; MDD/BP, major depression disorder/bipolar disorder; OCD, obsessive–compulsive disorder; PTSD, posttraumatic stress disorder; SAD, social anxiety disorder; SD, substance dependence; SZ, schizophrenia

**FIGURE 3:**
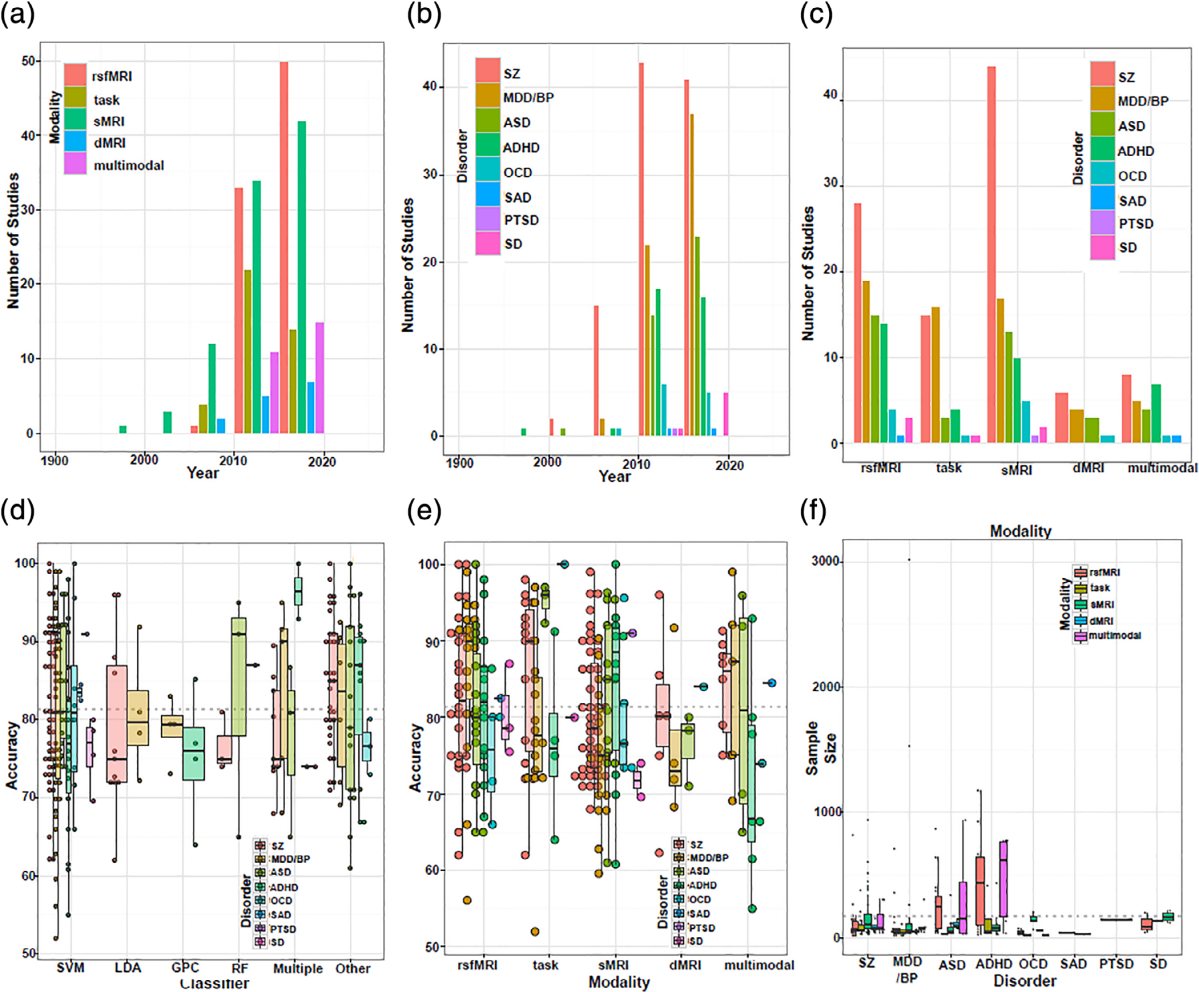
Visual summary of the surveyed mental illness prediction studies. (a) The number of studies published in each year for each modality. (b) The number of studies published in each year for each disorder type. (c) The number of studies published in each year for each disorder type and each modality. (d) The overall prediction accuracy against the commonly used classifiers in each disorder type. (e) The overall prediction accuracy against each modality and each disorder type. (f) The total sample size against each disorder type for each modality

**FIGURE 4:**
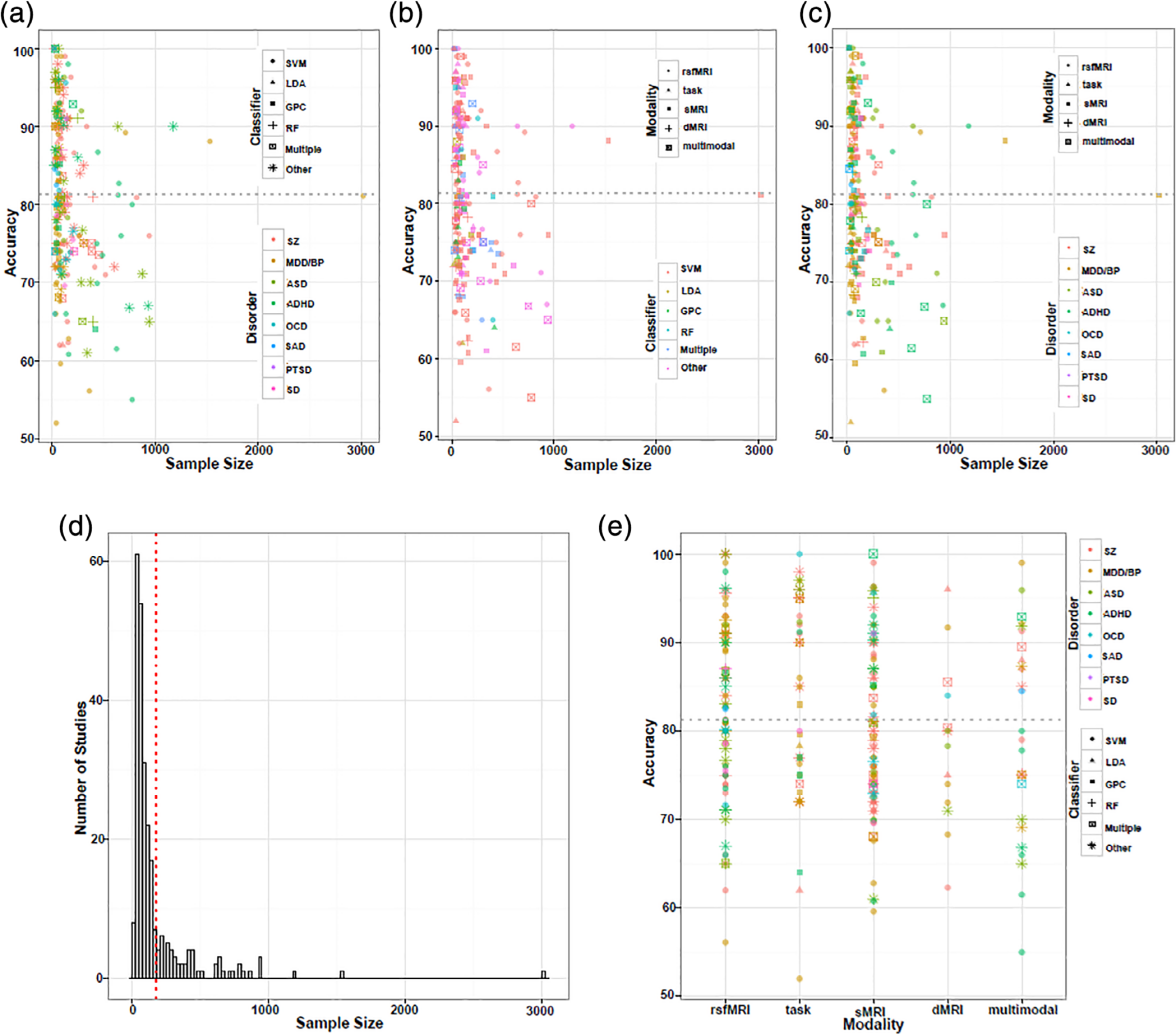
Visual summary of the surveyed mental illness prediction studies. (a) The overall accuracy against the total sample size for each disorder and for each classifier used in the studies. (b) The overall accuracy against the total sample size for each modality and for each classifier used in the studies. (c) The overall accuracy against the total sample size for each disorder and each modality used in the studies. (d) The sample size distribution for number of studies in the survey. (e) The overall accuracy against each modality for each disorder and for each classifier used in the studies

**FIGURE 5:**
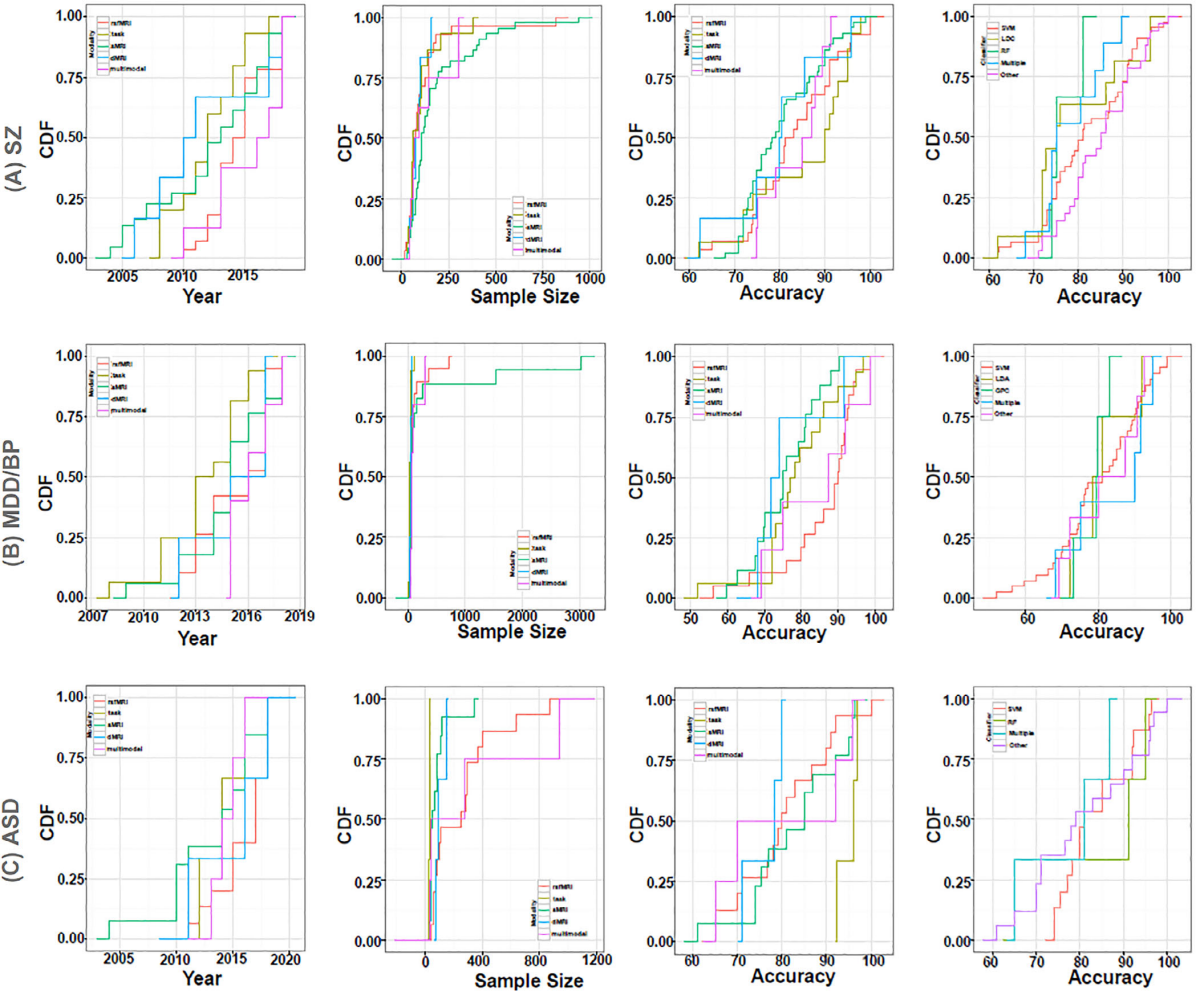
Disorder‐specific cumulative density function (CDF) of the surveyed mental illness prediction studies. Summary results are shown for (a) schizophrenia (SZ), (b) major depression disorder/ bipolar disorder (MDD/BP), (c) autism spectrum disorder (ASD). For each of the disorders, CDFs for publication year per modality, sample size per modality, accuracy per modality, and accuracy per classifier are presented

**FIGURE 6:**
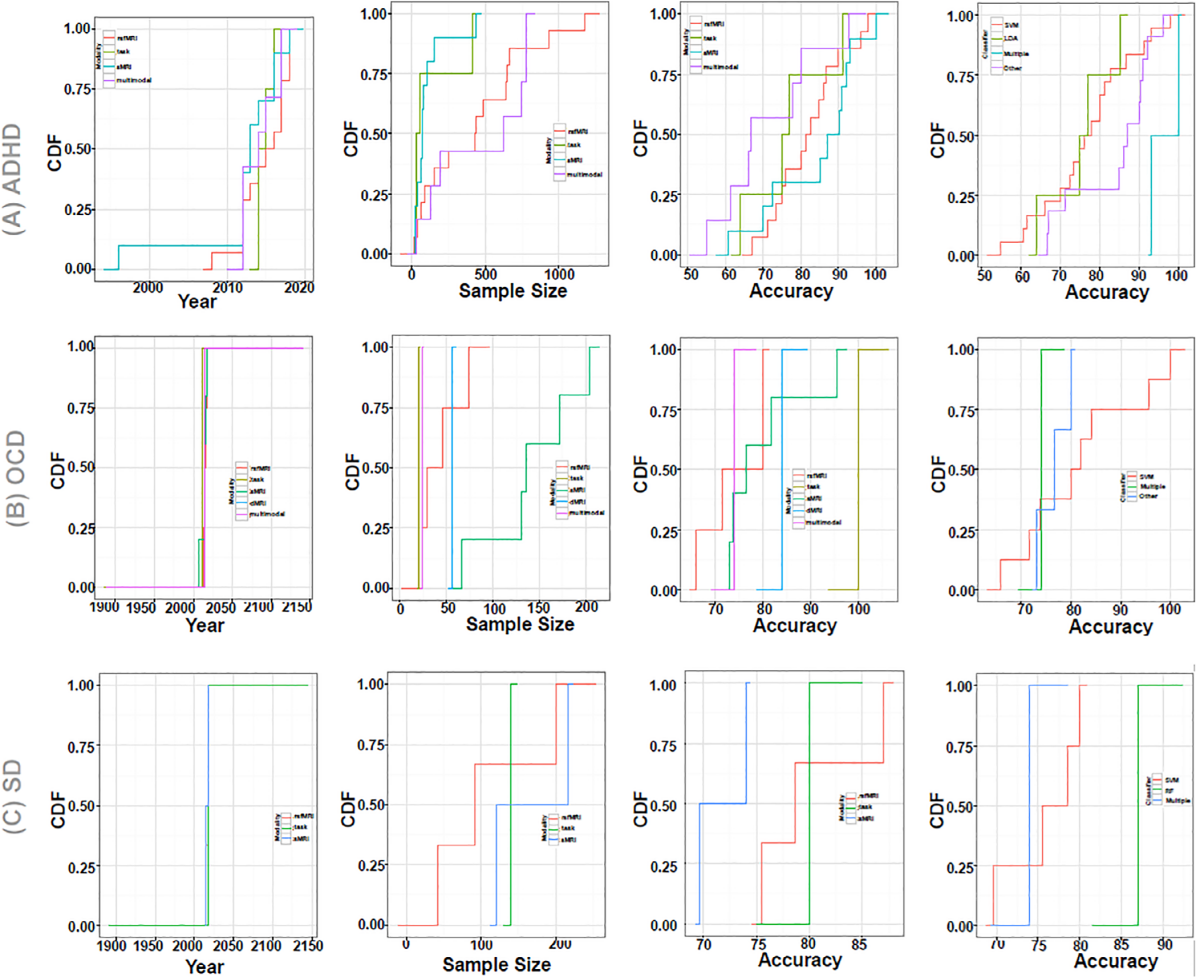
Disorder‐specific cumulative density function (CDF) of the surveyed mental illness prediction studies. Summary results are shown for (a) attention‐deficit/hyperactivity disorder (ADHD), (b) obsessive–compulsive disorder (OCD), and (c) substance dependence (SD). Summary for posttraumatic stress disorder (PTSD) and social anxiety disorder (SAD) were excluded due to very few publication number. For each of the disorders, CDFs for publication year per modality, sample size per modality, accuracy per modality, and accuracy per classifier are presented

The current review is based on a comprehensive literature search for research articles performing MRI‐based predictive analyses of psychiatric illnesses. A systematic literature search was performed primarily in PubMed from 1990 to 2018, and more than 550 articles were found. SZ (Calhoun, Kiehl, Liddle, & Pearlson, 2004) was one of the first disorders investigated with predictive analyses, followed by major depressive disorder (MDD; Fu et al., 2008; Marquand, Mourão‐Miranda, Brammer, Cleare, & Fu, 2008) and BP disorder (Arribas, Calhoun, & Adali, 2010), ADHD (C.‐Z. Zhu et al., 2008), ASD (Ecker et al., 2010), PTSD (Q Gong et al., 2014), OCD (Weygandt et al., 2012), SAD (Liu et al., 2015) and SD (Vergara, Mayer, Damaraju, Hutchison, & Calhoun, 2017; Vergara, Weiland, Hutchison, & Calhoun, 2018). Figure 2 illustrates the systematic literature search process for this current study. Briefly, the search consistent of the following steps: (a) different terms related to classification/machine learning as well as their abbreviations (e.g., for support vector machine, search with the term “SVM”), (b) all terms and abbreviations related to structural, functional and diffusion MRI (dMRI) combined with the term “biomarker”, and (c) all terms and abbreviations for one of the eight psychiatric disorders mentioned above. These steps were repeated for all disorders, and the identified references were further checked for missed publications which were included in the review as well. An additional screening process included on the relevance of the publications for the current review. Finally, we focused on all publications using a predictive analyses approach on MRI‐based data in a case–control design of mental illness diagnoses that explicitly evaluated classification performance measures (e.g., overall classification accuracy). Further, the same search procedure was repeated in Google Scholar in order to reduce the probability of missing relevant articles of interest. About 250 papers were eventually selected for this survey that includes: 101 SZ, 61 MDD/BP, 35 ADHD, 38 ASD, 1 PTSD, 12 OCD, 2 SAD, and 7 SD. We categorized these articles based on a scheme developed for this review as depicted in Figure 1a–e and a summary of all articles is presented in Tables 1, 2, 3, 4, 5, 6, 7, 8. Further, we limited our search range to journal articles in English published up until December 2018. Search criteria also included exclusion of articles without available full‐text, and similar papers published by the same authors. For each study, key aspects such as imaging modality, classification method, sample size, and type features were investigated in a quantitative manner, as seen in Figures 3, 4, 5, 6.

**TABLE 1:** Schizophrenia

References	Disorder	Modality	Number of subjects	Feature type	Classifier	Overall accuracy
Caprihan, Pearlson, and Calhoun (2008)	SZ	dMRI	HC = 45, SZ = 45, Total = 90	Discriminant PCA of FA maps	Fisher's LDC	80%
Caan et al. (2006)	SZ	dMRI	HC = 24, SZ = 34, Total = 5	FA map	LDA	75%
Ardekani et al. (2011)	SZ	dMRI	HC = 50, SZ = 50, Total = 100	Voxels of FA and MA maps reduced by PCA	LDA	96%
Honorio, Tomasi, Goldstein, Leung, and Samaras (2012)	SZ	fMRI (SM, AOD, WMT)	HC = 15, SZ = 13, Total = 28	Mean activation of the largest activation cluster	Majority vote of three decision stumps	96%
Demirci et al. (2008)	SZ	fMRI (AOD/Sternberg/SM tasks)	HC = 91, SZ = 57, Total = 138	ICA spatial maps	Projection pursuit	80–90%
Yoon et al. (2012)	SZ (first episode)	fMRI (AX‐CPT task)	HC = 51, SZ = 51, Total = 102	Voxels of left DLPFC in the contrast map	LDA	62%
Koch et al. (2015)	SZ	fMRI (monetary incentive delay task)	HC = 44, SZ = 44, Total = 88	MVPA of task activation pattern (best result for right palladium)	Searchlight SVM	93%
Cao, Duan, Lin, Calhoun, and Wang (2013)	SZ	fMRI (SM task) and SNP	HC = 116, SZ = 92, Total = 208	Sparse representation based variable selection	Sparse representation‐based classifier	77%
Costafreda et al. (2011)	SZ/BP	fMRI (verbal fluency task)	HC = 40, SZ = 32, BP = 40, Total = 104	Thresholded voxels in activation map by ANOVA tests	SVM	92%
Yoon et al. (2008)	SZ	fMRI (visual task)	HC = 15, SZ = 19, Total = 34	Selected active voxels from the contrast map	MVPA	59–72%
Bleich‐Cohen et al. (2014)	SZ with and without OCD	fMRI (WMT task)	HC = 20, SZ (with OCD) = 16, SZ (without OCD) = 17, Total = 53	MVPA on GLM contrast values	SVM	75–91%
Castro, Gómez‐Verdejo, Martínez‐Ramón, Kiehl, and Calhoun (2014)	SZ	fMRI (AOD task)	HC = 21, SZ = 31, Total = 52	ICA spatial maps of magnitude and phase data	Multiple kernel learning	85%
Castro, Martínez‐Ramón, Pearlson, Sui, and Calhoun (2011)	SZ	fMRI (AOD task)	HC = 54, SZ = 52, Total = 106	ICA (temporal and DMN network) and GLM spatial maps parcellated into AAL atlas	Recursive composite kernels	95%
Calhoun, Maciejewski, Pearlson, and Kiehl (2008)	SZ/BP	fMRI (AOD task)	HC = 26, SZ = 21, BP = 14, Total = 61	Distance to mean image for each group build using ICA spatial maps (DMN and temporal lobe)	Minimum distance	83–95%
Arribas et al. (2010)	SZ	fMRI (AOD task)	HC = 25, SZ = 21, BP = 14, Total = 60	ICA spatial maps (DMN and temporal lobe)	Bayesian generalized Softmax perceptron	82–90% (AUC)
W. Du et al. (2012)	SZ	fMRI (AOD task) and rsfMRI	HC = 28, SZ = 28, Total = 56	Kernel PCA on ICA spatial maps	Fisher's LDC	93–98%
Çetin et al. (2015)	SZ	fMRI (AOD task) and rsfMRI	HC = 28, SZ = 27, Total = 55	FNC scores derived from ICA‐based multinetwork fusion template	LDA and shaplet based classifier	72%
Yang, Liu, Sui, Pearlson, and Calhoun (2010)	SZ	fMRI (AOD task) and SNP	HC = 20, SZ = 20, Total = 40	Three types of features: Selected voxels in fMRI activation map, selected SNPs and ICA components	Majority voting among 3 SVMs	87%
Yu et al. (2013)	SZ	rsfMRI	HC = 25, SZ = 24, sibling, HC = 22, Total = 71	FC among 116 regions in AAL atlas reduced by PCA	SVM	62%
Yu, Shen, Zeng, Ma, and Hu (2013)	SZ/ MDD	rsfMRI	HC = 38, SZ = 32, MDD = 19, Total = 89	FC among ROIs	SVM	80.90%
Watanabe, Kessler, Scott, Angstadt, and Sripada (2014)	SZ	rsfMRI	HC = 74, SZ = 71, Total = 145	FC among 347 nodes placed as a grid in the entire brain	Fused lasso, GraphNet	91%
W. Guo et al. (2014)	SZ	rsfMRI	HC = 46, unaffected sibling of SZ patients = 46, Total = 92	fALFF values of the left ITG	SVM	75%
Venkataraman, Whitford, Westin, Golland, and Kubicki (2012)	SZ	rsfMRI	HC = 18, SZ = 18, Total = 36	FC among 90 ROIs	RF	75%
Tang, Wang, Cao, and Tan (2012)	SZ	rsfMRI	HC = 22, SZ = 22, Total = 44	FC among 90 ROIs reduced by PCA	SVM	93%
Su, Wang, Shen, Feng, and Hu (2013)	SZ	rsfMRI	HC = 32, SZ = 32, Total = 64	FC among 116 AAL regions	SVM	83%
Shen, Wang, Liu, and Hu (2010)	SZ	rsfMRI	HC = 20, SZ = 32, Total = 52	Dimension‐reduced FC (local linear embedding) among AAL ROIs	C‐means clustering	86%
J. Kim et al. (2016)	SZ	rsfMRI	HC = 50, SZ = 50, Total = 100	FC among 116 regions in AAL atlas	DNN	86%
Kaufmann et al. (2015)	SZ	rsfMRI	HC = 196, SZ = 71, Total = 267	FC based on ICA decomposition	Regularized LDC	75–84%
H. Cheng et al. (2015)	SZ	rsfMRI	HC = 29, SZ = 19, Total = 48	Graph measures of FC	SVM	80.00%
Fekete et al. (2013)	SZ	rsfMRI	HC = 10, SZ = 8, Total = 18	Local and global complex network measures	SVM	100%
Fan et al. (2011)	SZ	rsfMRI	HC = 31, SZ = 31, Total = 62	FC patterns	Ensemble of SVM classifiers	85–87%
Chyzhyk, Savio, and Graña (2015)	SZ	rsfMRI	HC = 74, SZ = 72, Total = 146	Pearson correlation features derived from ReHo, ALFF, FALF and voxel‐mirrored homotopic connectivity	Ensemble of extreme learning machines	80–91%
Bassett, Nelson, Mueller, Camchong, and Lim (2012)	SZ	rsfMRI	HC = 29, SZ = 29, Total = 58	Size of connected components in graphs build from correlation among time‐courses for 90 AAL regions	SVM	75%
Arbabshirani, Kiehl, Pearlson, and Calhoun (2013)	SZ	rsfMRI	HC = 28, SZ = 28, Total = 56	FNC among ICA time‐courses	SVM (best)	96%
Anticevic et al. (2013)	SZ	rsfMRI	HC = 90, SZ = 90, Total = 180	MVPA based on whole brain thalamic connectivity map	SVM	73.90%
A. Anderson and Cohen (2013)	SZ	rsfMRI	HC = 74, SZ = 72, Total = 146	Graph metrics based on FNC computed from ICA	SVM	65%
T. Zhang and Davatzikos (2013)	SZ	sMRI	HC = 79, SZ = 69, Total = 148	Voxels from five regions based on optimally discriminative VBM	SVM	71%
Zanetti et al. (2013)	SZ (first episode)	sMRI	HC = 62, SZ = 62, Total = 124	Whole brain volumetric measurements based on RAVENS	SVM	73%
Takayanagi et al. (2011)	SZ (first episode)	sMRI	HC = 40, SZ = 52, Total = 92	Volume and mean CT of selected ROIs	DFA	80%
Sun et al. (2009)	SZ and psychosis	sMRI	HC = 36, SZ = 36, Total = 72	Cortical GMD	Sparse multinomial LRCr	86%
Schnack et al. (2014)	SZ/BP	sMRI	HC1 = 66, HC2 = 43, SZ1 = 66, SZ2 = 46, BP1 = 66, BP2 = 47, Total1 = 198, Total2 = 136	Voxel‐wise GM maps	SVM	67–90%
(Radulescu et al., 2014)	SZ	sMRI	HC = 24, SZ = 27, Total = 51	Texture and volumetric measures	LDA	65.0–72.7%
Pina‐Camacho et al. (2015)	SZ	sMRI	HC = 42, SSD = 36, non‐SSD = 45, Total = 123	Clinical, neuropsychological, biochemical and volumetric measures	SVM	81.0–99.0%
Pardo et al. (2006)	SZ/BP	sMRI	HC = 8, SZ = 10, BP = 10, Total = 28	Volume of 23 ROIs along with 22 neuropsychological test scores	LDA	96%
Ota et al. (2012)	SZ	sMRI	HC = 105, HC2 = 23, SZ1 = 38, SZ2 = 23, Total = 189	GM and CSF volumetric measures of ROIs	LDA	70–76%
Nieuwenhuis et al. (2012)	SZ	sMRI	HC1 = 111, HC2 = 122, SZ1 = 128, SZ2 = 155, Total1 = 239, Total2 = 277	GMDs based VBM of top 10% voxels	SVM	71%
Nakamura et al. (2004)	SZ	sMRI	HC = 47, SZ = 57, Total = 104	Volume of several ROIs in the brain	LDA	78–86%
Koutsouleris et al. (2015)	SZ/MD	sMRI	MD = 104, SZ = 158, Total = 262	GM maps of regional analysis of brain volumes in normalized space (RAVENS)	SVM‐RFE	76%
Kawasaki et al. (2007)	SZ	sMRI	HC = 46, SZ = 46, Total = 92	The mean expression of Eigen image derived from VBM	Simple thresholding	80–90%
Kasparek et al. (2011)	SZ (first‐episode)	sMRI	HC = 39, SZ = 39, Total = 78	Whole brain voxel intensity values	Maximum‐uncertainty LDA	72%
Karageorgiou et al. (2011)	SZ (recent onset)	sMRI	HC = 47, SZ = 28, Total = 75	Volumetric measurements of 95 ROIs	LDA	72%
Janousova, Schwarz, and Kasparek (2015)	SZ	sMRI	HC = 49, SZ = 49	MR intensities, GMDs, and deformation based morphometry	Combination of mMLDA, centroid method, and the average linkage	81.60%
Iwabuchi, Liddle, and Palaniyappan (2013)	SZ	sMRI	HC = 20, SZ = 19, Total = 39	GM and WM maps	SVM	66.6–77%
Ingalhalikar et al. (2012)	SZ (identifying subtypes)	sMRI	HC = 29, SZ = 23, Total = 52	Multi‐edge graphs build from structural connectivity networks with 78 ROIs	Spectral clustering	78%
Greenstein, Weisinger, Malley, Clasen, and Gogtay (2012)	SZ (childhood onset)	sMRI	HC = 99, SZ = 98, Total = 197	CT	RF	74%
Gould et al. (2014)	SZ (cognitive deficit and cognitive spared)	sMRI	HC = 163, SZ = 208, SZA = 41, Total = 412	Whole brain VBM	SVM	56–72%
Fan, Shen, Gur, Gur, and Davatzikos (2007)	SZ	sMRI	HC1 = 38, HC2 = 41, SZ1 = 23, SZ2 = 46, Total 1 = 61, Total 2 = 87	Volumetric measurements based on deformation‐based morphometry	SVM	91%
Fan, Shen, and Davatzikos (2005)	SZ	sMRI	HC = 38, SZ = 23, Total = 61	Volumetric measures of all WM, GM, and CSF	SVM‐RFE	92%
Davatzikos et al. (2005)	SZ	sMRI	HC = 79, SZ = 69, Total = 148	Whole brain volumetric measurements	Nonlinear classifier (n.s.)	81%
Csernansky et al. (2004)	SZ	sMRI	HC = 65, SZ = 52, Total = 117	Hippocampal and thalamic shape eigenvectors	DFA	79%
Castellani et al. (2012)	SZ	sMRI	HC = 54, SZ = 54, Total = 108	Visual words extracted from DLPFC by SIFT and clustered by k‐means	SVM with local kernel	66–75%
Bansal et al. (2012)	SZ	sMRI	HC = 40, SZ = 65, Total = 105	Surface morphological measures	Semi‐supervised (hierarchical clustering)	94.00%
Ota et al. (2013)	SZ/ MDD	sMRI and dMRI	MDD = 25, SZ = 25, Total = 50	Volume and FA of insula, thalamus, ACC, ventricles and corpus callosum	LDA	72–88%
Sui et al. (2013)	SZ	sMRI, rsfMRI, and dMRI	HC = 28, SZ = 35, Total = 63	GMDs from structural, FA from DTI and ALFF from fMRI	SVM	79%
Yushkevich et al. (2005)	SZ	sMRI	SZ = 46, HC = 46, Total = 92	Normalized regional volumes	N.S.	71%
Davatzikos, Shen, et al. (2005)	SZ	sMRI	SZ = 69, HC = 69, Total = 138	GM, WM, and ventricular CSF volumes	N.S.	81%
Shi et al. (2007)	SZ	sMRI	SZ = 48, HC = 35, Total = 83	ReHo	Pseudo‐Fisher LDA	80%
Pohl and Sabuncu (2009)	SZ	sMRI	SZ = 16, HC = 17, Total = 33	Structure‐specific transformation parameters	SVM	90%
Rathi et al. (2010)	SZ	dMRI	SC = 21, HC = 20, Total = 41	Diffusion measures	Multiple classifiers	85.50%
Mueller, Wang, Pan, Holt, and Liu (2015)	SZ	rsfMRI	SC = 31, HC = 37, Total = 68	Regional specialization of caudate nucleus	SVM	74%
Pouyan and Shahamat (2015)	SZ	rsfMRI	SZ = 10, HC = 10, Total = 20	Independent components	Majority vote method using multiple classifiers	100%
Lee et al. (2018)	SZ	sMRI and dMRI	SZ = 47, HC = 23, Total = 70	Structural measures (volume and/or FA)	RF and SVM	87.6% ‐ 89.5%
Pergola et al. (2017)	SZ	sMRI	SZ = 96, nonaffected siblings = 55, HC = 249, Total = 400	Thalamic GMV	RF	81% ‐ 75%
Y. Liu et al. (2018)	SZ	rsfMRI	Adolescent‐onset schizophrenia (AOS) = 48, HC = 31, Total = 79	Cohe‐ReHo	SVM	89.87%
Winterburn et al. (2017)	SZ	sMRI	Dataset 1: SZ = 88, HC = 103, Dataset 2: SZ = 91, HC = 67, Dataset 3: SZ = 50, HC = 50, Total = 449	Tissue density estimates and CT	LR, SVM and LDA	73.5% (best)
Huang et al. (2018)	SZ	rsfMRI	SZ = 41, HC = 23, Total = 64	FC between 116 ROIs from AAL atlas	SVM	81.30%
J. Shao et al. (2017)	SZ	dMRI	SZ = 21, MDD = 25, HC = 25, Total = 71	Structural connectivity from ROIs	Multiple classifiers	80.4% (best)
Liu et al. (2017)	SZ	sMRI and dMRI	SZ = 62, HC = 33, Total = 95	GM and WM features	MFMK‐SVM	91.28%
Vyškovský, Schwarz, Janoušová, and Kašpárek (2016)	SZ	sMRI	SZ = 52, HC = 52, Total = 104	GMD	MLP and SVM	68% (best)
Ulloa, Plis, and Calhoun (2018)	SZ	sMRI and fMRI	SZ = 135, HC = 169, Total = 304	GM map and ALFF maps	Multi‐layer perceptron model	85% (ROC)
S. Liang et al. (2018)	SZ	sMRI and dMRI	Dataset 1: First‐episode schizophrenia (FES) = 98, HC = 106; Dataset 2: FES = 54, HC = 48, Total = 306	Regional GMV, CT, gyrification, FA, and MD	GBDT	75.05%
P Mikolas et al. (2016)	SZ	rsfMRI	Schizophrenia‐spectrum disorder (FES) = 63, HC = 63, Total = 126	Seed‐based FC maps	SVM	73.00%
de Pierrefeu et al. (2018)	SZ	sMRI	SZ = 276, HC = 330, Total = 606	Multiple structural measures (GM VBM, vertex‐based CT)	Enet‐TV penalty	72%
Moghimi, Lim, and Netoff (2018)	SZ	rsfMRI	Chronic SZ = 52, first episode = 30, HC = 88, Total = 170	Graph‐theoretic measures	SVM	80%
Janousova, Montana, Kasparek, and Schwarz (2016)	SZ	sMRI	First‐episode SZ = 52, HC = 52, Total = 104	GM volumes and local deformation from DBM	Multiple classifiers	83.7% (best)
Salvador et al. (2017)	SZ	sMRI	SZ = 128, BP = 128, HC = 127, Total = 383	sMRI features including GM and WM VBM, vertex‐based CT and volume, ROI volumetric measures and WBM maps	Multiple classifiers	75% (best)
Kottaram et al. (2018)	SZ	rsfMRI	SZ = 41, HC = 41, Total = 82	Static and dynamic FC measures	SVM	91%
Dagnew, Squarcina, Rivolta, Brambilla, and Sassi (2017)	SZ	sMRI	SZ = 20, HC = 20, Total = 40	Features characterizing brain anatomy	SVM and KNN	74%
Lu et al. (2016)	SZ	sMRI	SZ = 42, HC = 42, Total = 84	GMV and WMV between	SVM	88.40%
Tas et al. (2018)	SZ	sMRI	SZ = 23, schizo‐obsessive = 23, Total = 46	Voxel of‐interest values	SVM	78.26%
W. Cheng et al. (2015)	SZ	rsfMRI	SZ = 415, HC = 405, Total = 820	Resting‐state thalamocortical FC	SVM	73.53% –80.92%
Liu et al. (2017)	SZ	sMRI	SZ = 38, HC = 38, Total = 76	CT	SVM‐RBF	88.72%
Pavol Mikolas et al. (2018)	SZ	dMRI	First episode SZ = 77, HC = 77, Total = 154	FA	SVM	62.34%
Q. Zhu, Huang, and Xu (2018)	SZ	rsfMRI	SZ = 24, HC = 21, Total = 45	FC measures	KDA	95.56%
B. Cao et al. (2018)	SZ	rsfMRI	SZ = 43, HC = 29, Total = 72	FC measures	SVM	78.60%
Gheiratmand et al. (2017)	SZ	Task fMRI (AOD)	SZ = 184, HC = 196, Total = 380	Whole‐brain FC measures	Multiple classifiers	74%
Chin, You, Meng, Zhou, and Sim (2018)	SZ	sMRI	SZ = 141, HC = 71, Total = 212	GMD	SVM	86.6% (recognition)
Guo, Palaniyappan, Liddle, and Feng (2016)	SZ	sMRI	SZ = 98, HC = 83, Total = 181	CT	SVM	96.30%
Cabral et al. (2016)	SZ	rsfMRI and sMRI	SZ = 74, HC = 71, Total = 145	GM volume and FC measures	MVPA	75%
Rozycki et al. (2017)	SZ	sMRI	SZ = 440, HC = 501, Total = 941	Regional volumes, voxel‐wise measures, and complex distributed patterns	SVM	76%
Dwyer et al. (2018)	SZ	sMRI	SZ = 71, HC = 74, Total = 145	GMV	SVM	68.5–73%

**TABLE 2:** Major depression/bipolar disorder

References	Disorder	Modality	Number of subjects	Feature type	Classifier	Overall accuracy
Rubin‐Falcone et al. (2018)	BP/MDD	sMRI	BP = 26, MDD = 26, Total = 52	GM	SVM	75%
Deng et al. (2018)	BP/MDD	DTI	BP = 31, MDD = 36, Total = 67	FA	SVM	68.30%
Gao et al. (2017)	BP/MDD	rsfMRI	BP = 37, MDD = 36, Total = 73	Spatial independent components	SVM	93.00%
Jing et al. (2017)	cMDD/rMDD	rsfMRI	cMDD = 19, rMDD = 19, HC = 19, Total = 57	Hurst exponent	SVM	87.0% (cMDD vs. HC), 84.0% (rMDD vs. HC), 89.0% (cMDD vs. rMDD)
Yoshida et al. (2017)	MDD/BP	rsfMRI	MDD = 58, HC = 65, Total = 123	FC	PLS	80.00%
M. Li et al. (2017)	MDD/BP	rsfMRI	BP = 22, MDD = 22, Total = 44	Degree centrality	SVM	86.00%
Zhong et al. (2017)	MDD	rsfMRI	MDD1 = 29, HC1 = 33; MDD2 = 46, HC2 = 57; Total1 = 62, Total2 = 103	FC	SVM	91.9% (first sample), 86.4% (second sample)
Wang, Ren, and Zhang (2017)	MDD	rsfMRI	MDD = 31, HC = 29, Total = 60	FC	SVM	95.00%
Schnyer, Clasen, Gonzalez, and Beevers (2017)	MDD	DTI	MDD = 25, HC = 25, Total = 50	FA	SVM	74.00%
Sundermann et al. (2017)	MDD	rsfMRI	MDD = 180, HC = 180, Total=	FC	SVM	45.0% ~ 56.1%
Bhaumik et al. (2017)	MDD	rsfMRI	MDD = 38, HC = 29, Total=	FC	SVM	76.10%
He et al. (2017)	MDD/BP	rsfMRI/sMRI	BP = 13, MDD = 40, HC = 33, Total=	FNC, GM	SVM	91.3% (three groups), 99.0% (BD vs. MDD)
Bürger et al. (2017)	MDD/BP	Task fMRI	BP = 36, MDD = 36, HC = 36, Total=	Contrast maps	SVM, GPC	72.00%
Hilbert, Lueken, Muehlhan, and Beesdo‐Baum (2017)	GAD/MDD	sMRI	GAD = 19, MDD = 14, HC = 24, Total=	GM	SVM	58.7% (GAD and MDD vs. HC), 68.1% (GAD vs. MDD)
Drysdale et al. (2017)	MDD (biotypes)	rsfMRI	MDD = 333(4 biotypes), HC = 378, Total=	FC	SVM	89.20%
Sankar et al. (2016)	MDD	sMRI	MDD = 23, HC = 20, Total=	GM, WM	SVM	70.00%
Frangou et al. (2017)	MDD/BP	Task fMRI	BP = 30, MDD = 30, Total=	Contrast maps	GPC	73.10%
Ramasubbu et al. (2016)	MDD	rsfMRI	MDD = 15, HC = 19, Total=	Spatial independent components	SVM	66.00%
W. Yang et al. (2016)	MDD	Task fMRI	MDD = 16, HC = 16, Total=	Contrast maps	SVM	75.00%
Rive et al. (2016)	MDD/BP	sMRI/rsfMRI	MDDr = 23, BPr = 26, MDDd = 22, BPd = 10, Total = 81	GM, spatial independent components	GPC	69.10%
N.‐F. Jie et al. (2015)	MDD/BP	sMRI/rsfMR	BP = 21, MDD = 25, Total = 46	GM, fALFF	SVM	92.10%
Foland‐Ross et al. (2015)	MDD	sMRI	MDD = 18, HC = 15, Total = 33	CT	SVM	69.70%
Sacchet, Livermore, Iglesias, Glover, and Gotlib (2015)	MDD/BP	sMRI	BP = 40, MDD = 57, HC = 61, Total = 158	GM	SVM	59.5% (BP vs. MDD), 62.8% (MDD vs. HC)
Sacchet, Prasad, Foland‐Ross, Thompson, and Gotlib (2015)	MDD	DTI	MDD = 14, HC = 18, Total = 32	Graph metric of WM connectivity	SVM	71.90%
(João R Sato et al. (2015))	MDD	Task fMRI	MDD = 25, HC = 21, Total = 46	Contrast maps	LDA	78.30%
Johnston, Steele, Tolomeo, Christmas, and Matthews (2015)	MDD	sMRI	MDD = 20, HC = 21, Total = 41	GM	SVM	85.00%
Johnston et al. (2015)	MDD	Task fMRI	MDD = 19, HC = 21, Total = 40	Contrast maps	SVM	97.0% (best)
Koutsouleris et al. (2015)	MDD	sMRI	MDD = 104, SZ = 158, Total = 262	GM	SVM	76.00%
(Shimizu et al. (2015))	MDD	Task fMRI	MDD = 31, HC = 31, Total = 62	Contrast maps	gLASSO, SVM	95.0% (best)
Fung et al. (2015)	MDD/BP	sMRI	BP = 16, MDD = 19, Total = 35	CT and surface area	SVM	74.30%
Rosa et al. (2015)	MDD	Task fMRI	MDD = 19, HC = 19, Total = 38	FC	SVM	85.00%
Patel et al. (2015)	MDD	rsfMRI, sMRI, DTI	MDD = 33, HC = 35, Total = 68	rsfMRI, sMRI, DTI	Decision tree	87.30%
Redlich et al. (2014)	MDD/BP	sMRI	BP = 58, MDD = 58, Total = 116	GM	GPC	79.30%
L. Cao et al. (2014)	MDD	rsfMRI	MDD = 39, HC = 37, Total = 76	FC	SVM	84.00%
MacMaster, Carrey, Langevin, Jaworska, and Crawford (2014)	MDD/BP	sMRI	BP = 14, MDD = 32, Total = 43	GM	LDA	81.00%
L. L. Zeng, Shen, Liu, and Hu (2014)	MDD	rsfMRI	MDD = 24, HC = 29, Total = 53	FC	MMC	92.5% (clustering), 92.5% (classification)
Rondina et al. (2014)	MDD	Task fMRI	MDD = 30, HC = 30, Total = 60	Voxel intensity	SVM	72.00%
H. Guo, Su, et al. (2014)	MDD	rsfMRI	MDD = 36, HC = 27, Total = 63	FC	NN	90.50%
(Serpa et al. (2014))	MDD/BP	sMRI	BP = 23, MDD = 19, HC = 38, Total = 80	GM, WM, and ventricular RAVENS maps	SVM	54.8% (BD vs. MDD), 59.6% (MDD vs. HC)
Habes et al. (2013)	MDD	Task fMRI	MDD = 9, HC = 9, Total = 18	Contrast maps	LDA	72.20%
Wei et al. (2013)	MDD	rsfMRI	MDD = 20, HC = 20, Total = 40	Spatial independent components	SVM	90.00%
Grotegerd et al. (2014)	MDD/BP	Task fMRI	BP = 22, MDD = 22, Total = 44	Contrast maps	GPC	79.60%
Yu, Shen, Zeng, et al. (2013)	MDD	rsfMRI	MDD = 19, SZ = 32, Total = 51	FC	SVM	80.90%
Modinos et al. (2013)	MDD	Task fMRI	MDD = 17, HC = 17, Total = 34	Contrast maps	SVM	77.00%
Ma, Li, Yu, He, and Li (2013)	MDD	rsfMRI	MDD = 19, HC = 18, Total = 37	ReHo	LDA	91.90%
Grotegerd et al. (2013)	MDD/BP	Task fMRI	MDD = 10, BP = 10, HC = 10, Total = 30	Contrast maps	SVM, GPC	90.00%
Fang et al. (2012)	MDD	DTI	MDD = 22, HC = 26, Total = 48	Anatomical connectivity	SVM	91.70%
Mwangi, Ebmeier, Matthews, and Douglas Steele (2012)	MDD	sMRI	MDD = 62, HC = 62, Total = 124	Voxel‐based feature (GM)	RVM	90.30%
Lord, Horn, Breakspear, and Walter (2012)	MDD	rsfMRI	MDD = 22, HC = 22, Total = 44	FC	SVM	99.00%
F. Liu et al. (2012)	TRD/TSD	sMRI	TRD = 18, TSD = 17, HC = 17, Total = 52	GM, WM	SVM	82.90%
L.‐L. Zeng et al. (2012)	MDD	rsfMRI	MDD = 24, HC = 29, Total = 53	FC	SVM	94.30%
Mourão‐Miranda et al. (2011)	MDD	Task fMRI	MDD = 19, HC = 19, Total = 38	Contrast maps	SVM	52.0% (true positive)
Hahn et al. (2011)	MDD	Task fMRI	MDD = 30, HC = 30, Total = 60	Contrast maps	GPC	83.00%
Nouretdinov et al. (2011)	MDD	Task fMRI	MDD = 19, HC = 19, Total = 38	Contrast maps	SVM	76.30%
Costafreda, Chu, Ashburner, and Fu (2009)	MDD	sMRI	MDD = 37, HC = 37, Total = 74	GM	SVM	67.60%
Fu et al. (2008)	MDD	Task fMRI	MDD = 19, HC = 19, Total = 38	Contrast maps	SVM	86.00%
Matsuo et al. (2018)	MDD/BP	sMRI	MDD = 596, BP = 158, HC = 777, Total = 1,531	GMV	SVM	88.1% (best)
Geng, Xu, Liu, and Shi (2018)	MDD	rsfMRI	MDD = 24, HC = 24, Total = 48	FC and EC	Linear and nonlinear SVM, KNN, LR	91.70%
Rubin‐Falcone et al. (2018)	MDD/BP	sMRI	MDD = 26, BP = 26, Total = 52	GMV	SVM	75%
Nunes et al. (2018)	BP	sMRI	BP = 853, HC = 2,167, Total = 3,020	Regional CT, surface area and subcortical volume	SVM	81.10%
J. Yang et al. (2018)	MDD	dMRI and sMRI	Test dataset: MDD = 147, HC = 52; validation dataset: MDD = 83, HC = 25, Total = 307	Multiple structural measures	Penalized LR, RF and SVM	75.10%

**TABLE 3:** Autism spectrum disorder

References	Disorder	Modality	Number of subjects	Feature type	Classifier	Overall accuracy
Ingalhalikar, Parker, Bloy, Roberts, and Verma (2011)	ASD	dMRI	TDC = 30, ASD = 45, Total = 75	FA and MD of selected ROIs	SVM	80%
Just, Cherkassky, Buchweitz, Keller, and Mitchell (2014)	ASD	fMRI (social interaction task)	HC = 17, TDC = 17, Total = 34	Activation of selected voxels processed by factor analysis	GNB	97%
Murdaugh et al. (2012)	ASD	fMRI (two language tasks and a theory‐of‐mind task	TD = 14, ASD = 13, Total = 30	AG, MPFC and PCC based FC maps	LR	96.00%
Deshpande, Libero, Sreenivasan, Deshpande, and Kana (2013)	ASD	Task fMRI and DMRI	TDC = 15, ASD = 15, Total = 30	Causal connectivity weights, FC values and FA values	SVM	95.90%
Uddin et al. (2013)	ASD	rsfMRI	TDC = 20, ASD = 20, Total = 40	ICA components of rsfMRI	LR	78.00%
Plitt et al. (2015)	ASD	rsfMRI	TD1 = 59, TD2 = 89, ASD1 = 59, ASD2 = 89, Total = 296	FC among ROIs	LR and SVM (best)	76.70%
Iidaka (2015)	ASD	rsfMRI	TDC = 328, ASD = 312, Total = 640	FC among 90 ROIs	Probabilistic neural network	90%
C. P. Chen et al. (2015)	ASD	rsfMRI	TDC = 126, ASD = 126, Total = 252	FC among 220 ROIs	RF	91%
J. S. Anderson et al. (2011)	ASD	rsfMRI	TD = 40, ASD = 40, Total = 80	FC among ROIs	Thresholding	79.00%
Wee, Wang, Shi, Yap, and Shen (2014)	ASD	sMRI	HC = 59, ASD = 58, Total = 117	Thickness and volumetric of ROIs along with interregional features	Multi‐kernel SVM	96.30%
Uddin et al. (2011)	ASD	sMRI	TD = 24, ASD = 24, Total = 48	Voxel‐wise GM and WM maps	SVM	92.00%
Segovia et al. (2014)	ASD	sMRI	HC = 40, ASD = 52, ASD‐sib = 40	GM volume map	SVM	80.0–85.0%
Jiao et al. (2010)	ASD	sMRI	HC = 16, ASD = 22, Total = 38	Regional thickness measurements extracted from SBM	Logistic model trees	87%
Gori et al. (2015)	ASD	sMRI	HC = 20, ASD = 21, Total = 41	Morphometric features of selected ROIs	SVM	74% (AUC)
Ecker, Rocha‐Rego, et al. (2010)	ASD	sMRI	HC = 22, ASD = 22, Total = 44	GM and WM maps	SVM	77%
Ecker et al. (2010)	ASD	sMRI	HC = 20, ASD = 20, Total‐40	Volumetric and geometric features of selected cortical locations	SVM	85%
Calderoni et al. (2012)	ASD	rsfMRI	TDC = 38, ASD = 30, Total = 76	Gray matter maps	SVM	80% (AUC)
Akshoomoff et al. (2004)	ASD	sMRI	TDC = 15, ASD = 52, Total = 67	Volumetric measures and cerebellar vermis area	DFA	92.3–95.8%
Libero, DeRamus, Lahti, Deshpande, and Kana (2015)	ASD	sMRI, dMRI and MRS	TD = 18, ASD = 19, Total = 37	Cortical thickness, FA and neurochemical concentration	Decision tree	91.90%
Zhou, Yu, and Duong (2014)	ASD	sMRI, rsfMRI	TDC = 153, ASD = 127, Total = 280	Volume of selected subcortical regions, fALFF, number of voxels and Z‐values of selected regions and global VMHC voxel Number	Random tree classifier	70%
F. Zhang et al. (2018)	ASD	dMRI	ASD = 70, TD = 79, Total = 149	FA and MD measures	SVM	78.33%
Bernas, Aldenkamp, and Zinger (2018)	ASD	rsfMRI	Dataset 1: ASD = 12, HC = 12; Dataset 2: ASD = 12, HC = 18, Total = 54	Time of in‐phase coherence	LDA and SVM	86.70%
Chanel et al. (2016)	ASD	Task fMRI (social stimuli)	ASD = 15, HC = 14, Total = 29	Beta map voxels	SVM‐RFE	69–92.3%
Dodonova, Belyaev, Tkachev, Petrov, and Zhukov (2016)	ASD	dMRI	ASD = 51, TD = 43, Total = 94	Graph spectral distributions	EMD‐based kernel	71% (AUC)
Tejwani, Liska, You, Reinen, and Das (2017)	ASD	rsfMRI	ASD = 147, HC = 146, Total = 293	Dynamic FC variability	Naive Bayes, RF, SVM and multilayer perceptron algorithm	65%
Katuwal, Baum, and Michael (2018)	ASD	sMRI	ASD = 15, non‐ASD = 18, Total = 33	Multiple brain morphometric features	RF	95% (AUC)
Bhaumik, Pradhan, Das, and Bhaumik (2018)	ASD	rsfMRI	ASD = 145, HC = 159, Total = 372	FC measures from ROIs	MVPA	70% (best)
Fredo, Jahedi, Reiter, and Müller (2018)	ASD	rsfMRI	Training: ASD = 160, HC = 160; validation: ASD = 40, HC = 40, Total = 400	FC measures from ROIs	RF	65%
Zhao, Zhang, Rekik, An, and Shen (2018)	ASD	rsfMRI	ASD = 54, HC = 46, Total = 100	FC measures	SVM	81%
Soussia and Rekik (2018)	ASD	sMRI	ASD = 155, HC = 186, Total = 341	Network‐based measures	Supervised ensemble classifier	61% (best)
Retico, Gori, Giuliano, Muratori, and Calderoni (2016)	ASD	sMRI	ASD = 41, HC = 40, Total = 81	Surface‐based measures	SVM	74% (AUC, best)
Ghiassian, Greiner, Jin, and Brown (2016)	ASD	rsfMRI & sMRI	ADHD dataset: ADHD = 279, HC = 490; ASD dataset: ASD = 77, HC = 94, Total = 940	sMRI and fMRI measures	HOG‐ MHPC	65.00%
Dekhil et al. (2018)	ASD	rsfMRI	ASD = 123, TD = 160, Total = 283	Higher level fMRI features from SAEs	Probabilistic SVM	92% (best)
L. Wang, Wee, Tang, Yap, and Shen (2016)	ASD	sMRI	ASD = 54, HC = 57, Total = 111	GM and WM features	SVM	75.40%
H. Chen et al. (2017)	ASD	rsfMRI	ASD dataset: ASD = 22, HC = 24, SZ dataset: SZ = 35, HC = 31, Total = 112	FC measures	MVPA	83%
Emerson et al. (2017)	ASD	rsfMRI	ASD = 11, non‐ASD = 48, Total = 59	FC measures	Linear kernel	100% (PPV)
Wong, Anderson, Zielinski, and Fletcher (2018)	ASD	rsfMRI	ASD = 403, HC = 468, Total = 871	Pearson's correlation matrices, eigenvalue‐regularized and log‐Euclidean transformed matrices	Logistic regression	71.10%
X. Xiao et al. (2017)	ASD	sMRI	ASD = 46, delayed development (DD) = 39, Total = 85	Regional CT, cortical volume, and cortical surface area	SVM, RF and Naıve Bayes	80.9% (best)

**TABLE 4:** Attention‐deficit hyperactive disorder

References	Disorder	Modality	Number of subjects	Feature type	Classifier	Overall accuracy
Hart et al. (2014)	ADHD	fMRI (stop task)	HC = 30, ADHD = 30, Total = 60	Whole brain GLM coefficient map	GPC	77%
Park, Kim, Seo, Lee, and Park (2016)	ADHD	fMRI (six task)	ADHD‐IA = 13, ADHD‐C = 21, Total = 34	Network measures based on FC values	SVM	91.20%
Hart et al. (2014)	ADHD	fMRI (temporal discrimination task)	HC = 20. ADHD = 20, Total = 40	Brain activation map	GPC	75.00%
(Zou & Hastie, 2005)	ADHD	rsfMRI	HC = 12, ADHD = 12, Total = 24	ReHo maps	PCA‐based fisher discriminative analysis	85.00%
Wang, Jiao, Tang, Wang, and Lu (2013)	ADHD	rsfMRI	HC = 23, ADHD = 23, Total = 46	ReHo maps	SVM	80.00%
Sidhu, Asgarian, Greiner, and Brown (2012)	ADHD	rsfMRI	HC = 429, ADHD‐I = 98, ADHD‐C = 141, Total = 668	FFT and different varation of PCA on the BOLD signals along with phenotypic measures	SVM	68.86–76%
Sato, Hoexter, Fujita, and Rohde (2012)	ADHD	rsfMRI	HC = 546, ADHD‐IA = 122, ADHD‐HI = 12, ADHD‐C = 249, Total = 929	ReHO, ALLF and RSN	LRC (best)	54% ADHD Subtype: 67%
D. Fair et al. (2013)	ADHD	rsfMRI	TDC = 455, ADHD‐I = 80, ADHD‐C = 112, Total = 647	Graph based features based on FC	SVM‐based MVPA	63.4–82.7%
Dey, Rao, and Shah (2014)	ADHD	rsfMRI	HC = 307, ADHD = 180, Total = 487	Graph‐based measures compressed by multi‐dimensional scaling	SVM	73.50%
Deshpande, Wang, Rangaprakash, and Wilamowski (2015)	ADHD	rsfMRI	TDC = 744, ADHD = 433, Total = 1,177	Directional connectivity measures	ANN	90%
Semrud‐Clikeman et al. (1996)	ADHD/dyslexia	sMRI	HC = 10, ADHD = 10, Dyslexia = 10, Total = 30	Morphometric measures of ROIs	Discriminant function analysis	60.0–87%%
Peng, Lin, Zhang, and Wang (2013)	ADHD	sMRI	HC = 55, ADHD = 55, Total = 110	CT measures	ELM	90.20%
Lim et al. (2013)	ADHD	sMRI	HC = 29, ASD = 19, ADHD = 29, Total = 77	Voxel‐wise GM volumetric measures	Gaussian process classifier	68.2–85.2%
Johnston et al. (2014)	ADHD	sMRI	HC = 34, ADHD = 34, Total = 68	WM maps	SVM	93%
Igual et al. (2012)	ADHD	sMRI	HC = 39 AHDH = 39, Total = 78	Caudate nucleus volumetric measures	Adaboost and SVM	72.50%
Chang, Ho, and Chen (2012)	ADHD	sMRI	HC = 226, ADHD = 210, Total = 436	Texture features based on isotropic local binary patterns on three orthogonal planes	SVM	69.90%
Bansal et al. (2012)	ADHD	sMRI	HC = 42, ADHD = 41, Total = 83	Surface morphometric measures	Semi supervised (hierarchical Clustering)	91.00%
A. Anderson et al. (2014)	ADHD	sMRI + rsfMRI +phenotypic data	TD = 472, ADHD = 276, Total = 748	Curvature index, folding index, Gaussian curvature, gray matter volume, mean curvature, surface area, thickness average, and thickness standard deviation along with functional connectivity measures and phenotypic data	NMF + decision tree	66.80%
Bohland, Saperstein, Pereira, Rapin, and Grady (2012)	ADHD	sMRI + rsfMRI	TDC = 491, ADHD = 285, Total = 776	Various anatomical, network and nonimaging measures	SVM	80.0% (AUC)
Iannaccone et al. (2015)	ADHD	sMRI and fMRI‐ task (flanker/NoGo)	HC = 18, ADHD = 18, Total = 36	Whole brain GLM coefficients and GM maps from VBM	SVM	61.1–77.8%
Dai, Wang, Hua, and He (2012)	ADHD	sMRI and rsfMRI	TCD = 402, ADHD = 222, Total = 624	Cortical thickness and GM maps from sMRI and ReHo and FC from rsfMRI	SVM and multi‐kernel learning	61.50%
Colby et al. (2012)	ADHD	sMRI and rsfMRI	TD = 491, ADHD = 285, Total = 776	Morphological measures, FC, power spectra and graph measures	Multiple SVM	55%
L. Shao, Xu, and Fu (2018)	ADHD	rsfMRI	Dataset 1: ADHD = 22, HC = 61; Dataset 2: ADHD, 24, HC = 61; Dataset 3: ADHD = 76, HC = 118; Dataset 4: ADHD = 98, HC = 179, Total = 639	FC measures using 116 ROIs from AAL atlas	Bi‐objective SVM	81.25% (best)
Wolfers et al. (2016)	ADHD	Task fMRI (stop signal task)	ADHD = 184, unaffected sibling = 103, HC = 128, Total = 415	Activation maps derived from three task regressor	GPC	64% (AUC)
Cicek, Akan, and Metin (2018)	ADHD	sMRI	ADHD = 15, HC = 11, Total = 26	Multiple structural features	KNN and naive Bayes	100%
Y. Li et al. (2016)	ADHD	rsfMRI	ADHD = 23, HC = 45, Total = 68	ROI time‐series	Kernel ELM	96.06%
Qureshi, Jo, and Lee (2017)	ADHD	rsfMRI	AHDH‐I = 30, ADHD‐C = 30, TDC = 30, Total = 90	Global connectivity	H‐ELM	71.11%
Miao and Zhang (2017)	ADHD	rsfMRI	ADHD = 82, HC = 72, Total = 154	fALFF	SVM	98.04%
Yao et al. (2018)	ADHD	rsfMRI	Dataset 1: ADHD = 112, HC = 77; Dataset 2: ADHD = 34, HC = 28, Total = 251	FC measures	Ensemble learning	80–86%
Y. Zhang, Tang, Chen, Zhou, and Wang (2018)	ADHD	rsfMRI	Dataset 1: ADHD = 25, HC = 23; Dataset 2: ADHD = 22, HC = 61; Dataset 3: ADHD = 24, HC = 62; Dataset 4: ADHD = 118, HC = 98, Total = 433	FC measures	SVM	75%
Chaim‐Avancini et al. (2017)	ADHD	sMRI and dMRI	ADHD = 67, HC = 66, Total = 133	Multiple structural features	SVM	66%
Qureshi, Oh, Min, Jo, and Lee (2017)	ADHD	sMRI and rsfMRI	Training: ADHD‐I = 53, ADHD‐C = 53, TD = 53; testing: ADHD‐I = 14, ADHD‐C = 14, TD = 14, Total = 201	CT, image intensity, volume, CT standard deviation, surface Area, and ANOVA based features	Linear ELM and SVM	92.857% (best)
Riaz, Asad, Alonso, and Slabaugh (2018)	ADHD	rsfMRI	Dataset 1: ADHD = 25, HC = 23; Dataset 2: ADHD = 22, HC = 61; Dataset 3: ADHD = 24, HC = 61; Dataset 4: ADHD = 118, HC = 98, Total = 442	Dense functional brain network and phenotypic data	SVM	86.7% (best)
C. Xiao et al. (2016)	ADHD	sMRI	ADHD = 32, HC = 15, Total = 47	CT of ROIs	Generalized Lasso	92%

**TABLE 5:** Obsessive–compulsive disorder

References	Disorder	Modality	Number of subjects	Feature type	Classifier	Overall accuracy
Soriano‐Mas et al. (2007)	OCD	sMRI	OCD1 = 72 HC2 = 72 rep. Cohort: OCD2 = 30 HC2 = 30, Total1 = 144, Total2 = 60	Mean difference value of OCD and controls	N.S.	76.6
Weygandt et al. (2012)	OCD	Task fMRI	OCD = 10 HC = 10, Total = 20	Voxel‐based feature set and	SVM	100
Shenas, Halici, and Çiçek (2014)	OCD	rsfMRI; task fMRI	OCD = 12 HC = 12, Total = 24	Region‐based feature set and	LDC; SVM	74
Li et al. (2014)	OCD	dMRI	OCD = 28 HC = 28, Total = 56	Voxel‐based feature set	SVM	84
Shenas, Halici, and Cicek (2013)	OCD	rsfMRI	OCD = 12, HC = 12, Total = 24	FC measures	SVM	66%
Gruner et al. (2014)	OCD	rsfMRI	OCD = 23, HC = 23, Total = 46	ICA components	LR	80.10%
Parrado‐Hernández et al. (2014)	OCD	sMRI	OCD = 86, HC = 86, Total = 172	Voxel‐based measures	SVM	73.87%
Yun, Jang, Kim, Jung, and Kwon (2015)	OCD	sMRI	OCD = 56, HC = 75, Total = 131	Structural covariance	SVM	90.7–95.6%
X. Hu et al. (2016)	OCD	sMRI	OCD = 33, HC = 33, Total = 66	Voxel‐based measures	SVM	81.82%
Sen et al. (2016)	OCD	rsfMRI	OCD = 16, HC = 13, Total = 29	Network measures	SVM	80%
Takagi et al. (2017)	OCD	sMRI	Main dataset OCD = 56, HC = 52; validation: OCD = 10, HC = 18, Total = 136	FC measures	Sparse LR	73%
Trambaiolli, Biazoli Jr, Balardin, Hoexter, and Sato (2017)	OCD	rsfMRI	OCD = 38, HC = 36, Total = 74	Regional measures	SVM	71.64%

**TABLE 6:** Social anxiety disorder

References	Disorder	Modality	Number of subjects	Feature type	Classifier	Overall accuracy
F. Liu, Guo, et al. (2015)	SAD	rsfMRI	SAD = 20, HC = 20, Total = 40	Region‐based feature set	SVM	82.5%
Frick et al. (2014)	SAD	Task fMRI; sMRI	SAD = 14, HC = 12, Total = 26	Voxel‐based feature set	SVM	84.5%

**TABLE 7:** Posttraumatic stress disorder

Reference	Disorder	Modality	Number of subjects	Feature type	Classifier	Overall accuracy		
Q Gong et al. (2014)	PTSD	sMRI	PTSD earthquake = 50 no‐PTSD earthquake = 50 HC = 40, Total = 140	Voxel‐based feature set	SVM	No‐PTSD vs. PTSD: 76.00	No‐PTSD vs. HC: 85.00	PTSD vs. HC: 91.00

**TABLE 8:** Substance dependence

References	Disorder	Modality	Number of subjects	Feature type	Classifier	Overall accuracy
X. Zhu, Du, Kerich, Lohoff, and Momenan (2018)	AUD	rsfMRI	HC = 46, AUD = 46, Total = 92	Within‐ and between‐network FC	RF	87%
Steele et al. (2018)	Heroin‐dependent (treatment completion)	Task fMRI	SD = 139	FNC measures	SVM	80.58%
Pariyadath, Stein, and Ross (2014)	Nicotine dependence (smoking status)	rsfMRI	Smoker = 21, nonsmoker = 21, Total = 42	FC measures	SVM	78.6% (best)
Ding, Yang, Stein, and Ross (2017)	Nicotine dependence	rsfMRI	Smoker = 100, nonsmoker = 100, Total = 200	Local measures and network measures	SVM	75.50%
Guggenmos et al. (2018)	Alcohol dependence	sMRI	HC = 97, AUD = 119, Total = 216	Regional GM	Weighted robust distance, SVM	74%
Ding, Yang, Stein, and Ross (2015)	Nicotine dependence	sMRI	Smoker = 60, nonsmoker = 60, Total = 120	Mean GM volumes	SVM‐RFE	69.60%
Vergara et al. (2017)	Nicotine dependence	rsfMRI	Dataset 1: mTBI = 48, HC = 48; Dataset 2: Smoker = 21, nonsmoker = 21, Total1 = 96, Total2 = 42	FNC measures	SVM	95.20%

### 3.2 Schizophrenia

SZ is a chronic mental disorder (Bhugra, 2005), which is typically characterized by cognitive problems, disintegration in perception of reality, auditory and/or visual hallucination, and a chronic course with lasting impairment (Heinrichs & Zakzanis, 1998). There is currently no standard clinical diagnostic test for SZ, and there has been considerable focus on identifying a biologically based marker using neuroimaging features which has shown some promise. We surveyed 101 peer‐reviewed articles, which are presented in Table 1. Calhoun et al. (2004), Davatzikos, Shen, et al. (2005) and Yushkevich et al. (2005) are among the first studies to perform predictive analyses on SZ using MRI‐based neuroimaging data (Table 1).

1. *Structural MRI*: By utilizing sMRI data, Davatzikos, Shen, et al. (2005) used voxel‐based feature set and applied a high‐dimensional nonlinear pattern classification approach to compute the degree of separation between SZ and HCs (HC). Using the leave‐one out CV (LOO‐CV), the authors reported 81% classification accuracy (for gender‐wise classification, 82% for women and 85% for men). Another study by Yushkevich et al. (2005) used SVM classifier and region‐based feature sets to discriminate SZ patients with 72% accuracy. More recently, Koutsouleris et al. (2009) used sMRI and a principal component feature selection approach, where based on the overall predictive performance of the feature selection algorithm, an optimal number of principal components was identified to predict SZ. This study is particularly of importance as it reported to reliably predict different subcategories of SZ, with a three‐class classification for SZ showing a maximal accuracy of 82%. Another large‐scale study with a sample size of 256 case–control as well as a similar sized replication cohort predicted SZ based on sMRI‐derived features with an accuracy of about 70% for both CV and replication study (Nieuwenhuis et al., 2012).
2. *Functional MRI*: More recently, a high number of studies used features from resting‐state and task functional MRI (fMRI) for predictive modeling of SZ, and achieved promising outcomes.
  (a) *Task‐based*: Studies using features from task‐based fMRI paradigms include experiments with verbal fluency, working memory and auditory oddball (Castro et al., 2011; Costafreda et al., 2011; Honorio et al., 2012). One of the first relatively large‐scale study (i.e., sample size >150) classified SZ using three different task‐based fMRI data (i.e., auditory oddball, Sternberg item recognition and sensorimotor tasks) of 155 participants from two sites (Demirci, Clark, Magnotta, et al., 2008). The authors applied a projection pursuit algorithm on ICA spatial maps, and achieved classification accuracies ranging between 80 and 90%, with sensorimotor task providing the best performance. Further, based on regions with greater synchrony estimated from synchronous hemodynamic independent maps of auditory cortex as features, Calhoun and colleagues used a within‐participant subtractive comparison to discriminate SZ from HC with 97% initial accuracy and 94% accuracy after a retest validation using a new subjects scanned at a different site (Calhoun et al., 2004). Another study focusing on three‐class differential diagnosis of SZ, BP and HC individuals reported that verbal fluency led to a reliable diagnostic specificity for SZ with 92% accuracy (Costafreda et al., 2011). Many psychotic disorders, such as SZ, schizoaffective and BP disorders may share substantial number of overlapping symptoms, risk genes, brain dysfunctions, and treatment response. Thus, it becomes very challenging to clinically differentiate these patients based on traditional diagnostic approaches. A few recent studies have investigated the heterogeneity associated with SZ and schizoaffective disorder, as well as BP disorder with psychosis (Cardno & Owen, 2014; Cosgrove & Suppes, 2013; Pearlson, Clementz, Sweeney, Keshavan, & Tamminga, 2016). Clementz and colleagues proposed a “biotypes” based approach where they identified three neurobiologically unique biologically defined psychosis categories and shown that biotypes did not follow a straightforward disease severity continuum, with heritable properties in unaffected first‐degree relatives (Clementz et al., 2015).
  (b) *Resting‐state*: The rsfMRI studies for SZ prediction included a variety of classifiers, such as, SVM, fused lasso, GraphNet, RF, C‐means clustering, regularized LDC, and ensembles of SVM classifiers, as presented in Table 1. Overall, the sample size was relatively large across these studies, with classification accuracies ranging from 62% to 100%, although the study with 100% accuracy rate had a very small sample size (20 participants) and therefore, the results might not be generalizable across other studies (Pouyan & Shahamat, 2015).
3. *Diffusion MRI*: The dMRI studies surveyed in this review reported accuracies ranging from 62% to 96%, with classifiers such as SVM, LDA, Fisher's LDC or combination of multiple classifiers (Ardekani et al., 2011; Caan et al., 2006; Caprihan et al., 2008; Mikolas et al., 2018; Rathi et al., 2010; J. Shao et al., 2017). Features from these studies included fractional anisotropy (FA) maps and structural connectivity from ROIs.
4. *Multimodal*: Recently, by using connectivity measures from multimodal dMRI and sMRI data, Zhu, Shen, Jiang, and Liu (2014) predicted SZ patients and achieved a perfect accuracy (i.e., 100%). However, given the small sample size (i.e., HC = 10 and SZ = 10) and the choice of CV (i.e., LOO‐CV), the framework might have introduced classification bias with lack of generalizability with independent study sample.

In general, the existing SZ predictome studies mostly used functional and structural MRI data as features and LOO‐CV approach as CV, with many studies having a very small sample size. Therefore, future studies examining predictive modeling with more robust CV approaches and greater small size are necessary. Further, while these initial results suggest that SZ can be predicted with higher accuracies, the accuracy range varies substantially across these studies, and replication studies are required to confirm generalizability.

### 3.3 Major depression/bipolar disorder

While the overlapping symptoms make it challenging to differentiate between MDD and BP as well as with other disorders (e.g., SZ and schizoaffective disorder), recent studies have reported successful diagnostic prediction of of BP and MDD (Arribas et al., 2010; Costafreda et al., 2011; Lueken, Hilbert, Wittchen, Reif, & Hahn, 2015; Redlich et al., 2014). We reviewed 61 studies that used neuroimaging for automatic diagnose BP and MDD which are listed in Table 2. Among the surveyed studies, structural MRI was primarily used to predict BP patients, although only few studies included BP samples. Therefore, further investigation is required prior to recommending particular classifiers or machine learning frameworks as diagnostic tools for BP prediction. As presented in Table 2, many studies have performed classification on MDD samples. While there is an increase in studies predicting MDD using rsfMRI, sMRI, and task‐based fMRI as features and primarily SVM classifier, with the accuracies ranging from 52% to 99%, the sample size is relatively small in most studies (Table 2).

1. *Structural MRI*: Costafreda et al. (2009) utilized sMRI data where they used gray matter maps as features from 37 MDD and 37 HC participants and achieved 67.6% classification accuracy. Another recent study with relatively large sample size (i.e., 62 MDD and 62 HC) used voxel‐based gray matter maps driven from sMRI and SVM classifier and achieved 90% accuracy (Mwangi et al., 2012). Few recent studies have also focused on cross‐disorder prediction of MDD and BP, as well as MDD and SZ. For instance, using multivariate patterns form gray matter differences and LOO‐CV, Redlich and colleagues differentiated MDD from BP patients with 79.3% accuracy, and further validated the findings using an independent dataset and test–retest validation with 65.5% accuracy (Redlich et al., 2014).
2. *Functional MRI*:

- *Task‐based*: Among the initial MDD prediction studies are two task‐based fMRI studies by Marquand et al. (2008) (verbal memory N‐back task, 67.5% accuracy) and Fu et al. (2008) (sad facial processing task, 84% accuracy).Other task‐based MRI studies include the works performed by Sato et al. (2015) (task paradigm included written statements of actions counter to social and moral values described by social,78.3% accuracy), Johnston, Tolomeo, et al. (2015) (instrumental loss‐avoidance and win–gain reinforcement learning task, 97% accuracy [best result]), and Shimizu et al. (2015) (semantic and phonological verbal fluency task, 95% accuracy [best result]).
- *Resting‐state*: Among the rsMRI studies, Craddock, Holtzheimer III, Hu, and Mayberg (2009) used region‐based feature set from rsfMRI and achieved 95% accuracy. Further, using rsfMRI data and SVM classifier, Yu, Shen, Zeng, et al. (2013) achieved 84.2% accuracy.
- *Diffusion MRI*: Studies utilizing features from dMRI data include SVM classifier based pipelines and features such as anatomical connectivity, graph matric of white matter connectivity, and FA, with classification accuracies ranging from 63% to 91% (Deng et al., 2018; Fang et al., 2012; Sacchet, Prasad, et al., 2015; Schnyer et al., 2017).
- *Multimodal*: Using sMRI and dMRI data and LDC classifier, Ota et al. (2013) obtained about 80% accuracy, to distinguish MDD from SZ and HC individuals. Another large‐scale MDD study (total sample size = 307) by J. Yang et al. (2018) used structural and dMRI data and reported 75% accuracy.

While these studies utilized a variety of MRI based features, such as sMRI, resting and task‐based fMRI and dMRI, they provide evidence of brain‐based differential diagnoses of MDD and BP. Future studies should employ large‐scale sample size, and more BP prediction research.

### 3.4 Autism spectrum disorder

ASD is a neurodevelopmental disorder characterized by impaired social communication, deficits in social–emotional reciprocity, deficits in nonverbal communicative behaviors used for social interaction and stereotypic behavior. Since 2010, a few studies have investigated automatic diagnosis of ASD in both male‐only and male–female samples (Ecker, Marquand, et al., 2010; Ecker, Rocha‐Rego, et al., 2010; Jiao et al., 2010) with promising results showing accuracies ranging from 81% to 90% Table 3). We surveyed 30 papers in automatic diagnosis of ASD using MRI‐based features which are listed in Table 3.

1. *Structural MRI*: Using voxel‐based feature set (Ecker, Rocha‐Rego, et al., 2010) and region‐based feature set (Ecker, Marquand, et al., 2010) from sMRI data and SVM classifiers, Ecker and colleagues predicted ASD with 81 and 90% accuracies, respectively. Interestingly, Uddin and colleagues employed a searchlight algorithm to sMRI features, where a small number of voxels within the spatial proximity with one another provide the predicting features (Uddin et al., 2011). The resulting model obtained 92% classification accuracy for ASD diagnosis.
2. *Functional MRI*:
  (a) *Task‐based*: More recently, features from task‐based fMRI and SVM, LRC, LDC, and GPC classifiers were used for ASD prediction, with accuracies ranging from 70% to 96% (Table 3).
  (b) *Resting‐state*: Other ASD prediction studies include region‐based feature set and automatic feature selection from resting fMRI data with 79% accuracy (J. S. Anderson et al., 2011) and ICA component based features from resting fMRI data with 78% accuracy (Uddin et al., 2013).
3. *Diffusion MRI*: Ingalhalikar and colleagues used region‐based feature set and automatic feature selection from dMRI data and achieved 80% accuracy (Ingalhalikar et al., 2011). Another dMRI‐based prediction study by Zhang and colleagues used SVM classifier on FA and mean diffusivity measures and reported to achieve 78% accuracy.
4. *Multimodal*: Studies utilizing multimodal MRI data for ASD prediction include the works by Deshpande and colleagues where both task fMRI and dMRI data were used to identify causal connectivity weights, connectivity values, and FA features, resulting in 95.9% accuracy (Deshpande et al., 2013). Another work discriminating between ASD and HC participants used rsfMRI and sMRI based features and achieved 65% accuracy (Ghiassian et al., 2016).

While these studies have utilized all available MRI data modalities as features for predicting ASD, the number of sample size as well as studies based on each modality are relatively low. Additionally, heterogeneity associated with ASD, including subtypes of lower versus higher functioning patients, should be further investigated using predictive models.

### 3.5 Attention‐deficit/hyperactivity disorder

One of the most commonly found neurodevelopmental disorders is ADHD. However, given the lack of biological‐based diagnosis approach, ADHD is currently diagnosed based on behavioral symptoms only. In this review, we surveyed 35 papers in automatic diagnosis of ADHD using MRI‐based features which are listed in Table 4.

1. *Structural MRI*: Studies based on sMRI features reported accuracies for ADHD classification ranging from about 72% to 93% (Igual et al., 2012; Johnston et al., 2014; Lim et al., 2013). Using voxel‐based feature set from sMRI data and automatic feature selection approach, Johnston and colleagues trained the features using a SVM classifier and obtained 93% accuracy (Johnston et al., 2014). Lim and colleagues used whole brain gray and white matter from sMRI data and GPC classifier and predicted ADHD with 79.3% accuracy (Lim et al., 2013)
2. *Functional MRI*:

- *Task‐based*: In order to predict ADHD, Hart and colleagues used stop signal task‐based fMRI data (Hart, Chantiluke, et al., 2014; 77% accuracy) and temporal discounting task‐based fMRI data (Hart, Marquand, et al., 2014; 75% accuracy). However, these studies were performed using relatively small sample sizes, and therefore may lack generalizability across other independent samples.
- *Resting‐state*: A recent study by Zhu and colleague used voxel‐based feature set and automatic feature from rsfMRI data and LDC and SVM classifiers to classify ADHD with an accuracy of 75–80% (C.‐Z. Zhu et al., 2008). Other resting‐state studies include the work by Sidhu and colleagues where they used SVM classifier in a large sample with 668 participants and obtained 76% accuracy (best result; Sidhu et al., 2012).

*Diffusion MRI*: While studies have reported disrupted white matter tracts in ADHD patients (van Ewijk, Heslenfeld, Zwiers, Buitelaar, & Oosterlaan, 2012), to date no published studies have examined dMRI based features for ADHD prediction.
*Multimodal*: Several multimodal studies for ADHD prediction include sMRI, rsfMRI, and phenotypic data (A. Anderson et al., 2014), sMRI and rsfMRI data (Bohland et al., 2012; Colby et al., 2012; Dai et al., 2012), and sMRI and task‐based MRI data (Iannaccone et al., 2015), with accuracy ranging from 55% to 80%.

### 3.6 Obsessive–compulsive disorder

Only a few recent studies have applied classification algorithms to OCD. We surveyed 12 papers focused on automatic diagnosis of OCD using MRI‐based features which are listed in Table 5.

1. *Structural MRI*: Using an approach to calculate the distance between individual participants and the mean of OCD and control group based on sMRI derived measures, Soriano‐Mas and colleagues distinguished between OCD patients and HCs (Soriano‐Mas et al., 2007). Other sMRI studies for OCD prediction reported overall accuracies ranging from 73% to 95.6% (X. Hu et al., 2016; Parrado‐Hernández et al., 2014; Takagi et al., 2017; Yun et al., 2015).
2. *Functional MRI*:

- *Task‐based*: In a task‐based fMRI study presenting emotional valence stimuli, OCD patients were classified with perfect accuracy in a very small sample of 10 participants per group (Weygandt et al., 2012).
- *Resting‐state*: The surveyed rsfMRI studies mostly used SVM classifier to predict OCD (Sen et al., 2016; Shenas et al., 2013; Trambaiolli et al., 2017; accuracy ranging from 66% to 80%), with an exception of logistic regression classifier (Gruner et al., 2014; 80% accuracy).
- *Diffusion MRI*: Li and colleagues used voxel‐based feature set from dMRI data and SVM classifier to classify OCD with 84% accuracy (Li, Tran, et al., 2014).
- *Multimodal*: Using features from multimodal resting‐state MRI and task MRI data Shenas and colleagues reported 74% accuracy for OCD prediction while applying SVM and LDC classifiers (Shenas et al., 2014).

### 3.7 Social anxiety

To date, only two studies on SAD have been published which included relatively small samples, with accuracies above 80% (Frick et al., 2014; Liu et al., 2015). These studies derived multivariate patterns from different MRI modalities suggesting that features relevant for SAD classification can be extracted and analyzed across modalities. Both studies reported useful features to be distributed across widespread brain areas, rather than localized brain regions typically associated with anxiety. We surveyed two papers focused on automatic diagnosis of SAD using MRI‐based features which are listed in Table 6.

### 3.8 Posttraumatic stress disorder

Only one study to date has performed discriminative analysis on PTSD, where 50 earthquake survivors with and without PTSD were compared to controls using structural imaging (Q Gong et al., 2014). Patients with PTSD were classified with an accuracy of 91%, with the most discriminative features were found in different brain areas, particularly in left and right parietal regions. Table 7 presents the surveyed paper in automatic diagnosis of PTSD using MRI‐based features.

### 3.9 Substance dependence

To date, only a few predictive studies on SD (e.g., alcohol, nicotine, and cocaine addiction) and treatment completion have been performed, with only one study implementing a multimodal imaging approach to predict alcohol consumption and treatment effects in the brain (Cosa et al., 2017), although using data from Marchigian–Sardinian (msP) rats. Table 8 provides the seven surveyed paper in automatic diagnosis of SD using MRI‐based features.

1. *Structural MRI*: Using SVM‐RFE classifier, Ding and colleagues utilized mean gray matter volume features from sMRI data to predict nicotine dependence with 69.6% accuracy (Ding et al., 2015). Alcohol dependence was predicted in a recent study using regional gray matter maps from sMRI and weighted robust distance and SVM classifiers (Guggenmos et al., 2018), resulting in 74%classification accuracy.
2. *Functional MRI*:
  (a) *Task‐based*: Only one study investigated heroin‐dependence using FNC measures from task‐based fMRI and SVM classifiers, and achieved 80.58% accuracy (Steele et al., 2018).
  (b) *Resting‐state*: Using FC measures as features from rsfMRI data and SVM classifiers, Pariyadath and colleagues predicted nicotine dependence with a maximum accuracy of 78.6% (Pariyadath et al., 2014). Ding and colleagues further used rsfMRI data and SVM based classifiers to successfully predict nicotine dependence (Ding et al., 2017; local measures and network measures, 75.5% accuracy). Alcohol Dependence was predicted in a recent study using rsfMRI based FC measures and Random Forest classifier, obtaining 87% accuracy (Zhu, Du, et al., 2018). Using connectivity measures from rsfMRI data, SVM classifier was used to predict nicotine dependence with 95.2% accuracy (Vergara et al., 2017).

### 3.10 Analysis of the survey

Figure 3 demonstrates some key features from this survey. Figure 3a presents the number of studies published in each year for each modality, Figure 3b shows the number of studies published in each year for each disorder type, and Figure 3c shows the number of studies published in each year for each disorder type and each modality. These figures show that there is a publication growth since 2007, and since 2010, the number of studies has been growing rapidly. Only one ADHD prediction study was performed before 2000, which included a sample size relatively low compared to the recent ADHD publications (Semrud‐Clikeman et al., 1996). Particularly, all major disorders’ studies (i.e., SZ, MDD/BP, ASD, and ADHD) have shown a peak between 2011 and 2013, which might be due to the recent data‐sharing initiatives. From Figure 3c, it can be observed that all MRI‐based features have been utilized for prediction of these major disorders, with a few multimodal studies. Further, it shows that structural MRI (sMRI) is the most popular choice of modality, particularly for SZ studies. For MDD/BP, ASD, and ADHD studies, rsfMRI is the most popular modality. Moreover, compared to dMRI, multimodal studies are more common across these major disorders.

Figure 3d shows the overall prediction accuracy against the commonly used classifiers in each disorder type, Figure 3e reports the overall prediction accuracy against each modality and each disorder type, and Figure 3f presents the total sample size against each disorder type for each modality. SVM classifier was the most popular across all major disorders, followed by LDA.

Figure 4a shows the overall accuracy against the total sample size for each disorder and for each classifier used in the studies, Figure 4b shows the overall accuracy against the total sample size for each modality and for each classifier used in the studies, and Figure 4c shows the overall accuracy against the total sample size for each disorder and each modality used in the studies. Interestingly, even with a sample size smaller than 100, almost all studies reported very high accuracies, with few reporting 100% classification accuracy. The reported overall accuracy decreases with increasing sample size in most of the major disorders, including SZ and MDD/BP, which is concerning as it indicates that the classification framework utilized in these small sample size studies might not be generalized across large‐scale studies. Further, SVM classifier shows to achieve very high accuracy across most of the major disorders. Also, rsfMRI and task‐based SZ, MDD/BP and ADHD studies show high accuracy rate.

Figure 4d reports the sample size distribution for number of studies in this survey. The dashed lines represent mean (red) sample size across all studies. Figure 4e shows the overall accuracy against each modality for each disorder and for each classifier used in the studies. MDD/BP and ADHD studies reported the lowest accuracies.

Based on our surveyed studies (Tables 1, 2, 3, 4, 5, 6, 7, 8), volume and cortical thickness from structural MRI, the activation maps and functional connectivity among ROIs or ICA components from fMRI data and FA from dMRI data were some of the most commonly used features for classification. Further, SVM was the most popular classifier of choice across all disorders’ studies, and different extensions of SVM methods, including linear, nonlinear (different kernel), and SVM with recursive feature elimination (SVM‐RFE), were observed. Other popular classifiers from the surveyed studies include LDA, Gaussian process classifier (GPC) and random forest (RF).

Figures 5 and 6 present the cumulative density function (CDF) for (a) publication year per modality, (b) sample size per modality, (c) accuracy per modality, and (d) accuracy per classifier, for the main disorders (disorders with very few number of studies were excluded). An interesting observation from this summary is that, there is a growth in multimodal prediction studies for many of these major mental disorders.

## 4 TRANSLATIONAL PERSPECTIVE OF BRAIN‐BASED PREDICTOME RESEARCH FOR CLINICAL APPLICATION

### 4.1 Translating predictive outcomes toward clinical utility

Typically, in a research‐based setup, predictive studies are implemented using two or more well‐proportioned groups of patients with mental illness and their healthy counterparts. The group labels are carefully diagnosed before training a supervised classification algorithm, where exclusion of subjects with uncertain diagnoses or comorbidities is a common practice (Wolfers et al., 2015). However, in case of real clinical population, the disease diagnosis is a more complex and sophisticated process. Thus, considerable improvement in the field of predictive modeling is required before they will be useful to apply in clinical practice. In many clinical cases, the central question to be addressed is not as simple as how to distinguish patients from controls, but rather the specific distinction between different illnesses in the same population (i.e., subtypes). Simply put, a differential diagnostic process is required before accurate clinical implementation of the available tools can be made available. Moreover, another limitation of the current predictive modeling approaches is the lack of appropriate (or any) identification of comorbidities among patients, which is essential to properly assign memberships across multiple diagnostic classes. To date, there are only a few existing studies in the current literature across different disorders have demonstrated multi‐class classification (i.e., subtype classification) of three or more disorders (Costafreda et al., 2011; Du et al., 2015; Dwyer et al., 2018; Grotegerd et al., 2013; Koutsouleris et al., 2015; Lim et al., 2013; Redlich et al., 2014; Serpa et al., 2014; Yu, Shen, Zeng, et al., 2013). However, to the best of our knowledge, no such studies have adequately addressed the issue of comorbidity.

Although existing limitations should be addressed before clinical application of the research‐based diagnostic/predictive tools become available, even current approaches can be used as a supplement to clinical diagnosis with higher decision‐making uncertainty or even to make decisions for any follow‐up medical procedure requirements. Beyond the traditional subjective approaches, incorporating quantitative prediction approaches of psychiatric disorders can assist more accurate clinical diagnosis (Krueger et al., 2018). However, there are several key factors that need to be considered prior to a successful clinical translations of the neuroimaging predictive modeling, including reliability of neuroimaging‐based findings and subject‐level clinical transition (Saggar & Uddin, 2019). Moreover, some of the existing gaps in psychiatric neuroimaging research need to be addressed (Etkin, 2018, 2019).

More specifically, some immediate clinical application of predictive modeling may include:

- *Diagnosis based on “biomarkers” in addition to clinical interviews*: Current clinical diagnoses are primarily made based on clinical interviews. In addition to the existing behavioral measures, predictive modeling outcomes can provide more accurate brain‐based endophenotypes or “biomarkers”, and combining both measures can provide deeper understanding of biological pathways, mechanisms and progression of the disease. For example, brain‐based biomarkers for ASD and ADHD could aid in disease diagnosis more objectively (Uddin, Dajani, Voorhies, Bednarz, & Kana, 2017).
- *Multi‐class classification*: In clinical practice, it is very challenging to diagnose severe mental disorders that share substantial overlapping symptoms such as SZ, schizoaffective, unipolar and bipolar depression, and mood disorders. By obtaining estimates of predictive confidence level across multiple disease classes, predictive models could offer to identify comorbidities in patient groups. Recent multi‐class approaches can specify a predictive value for all classes of interest for each subject, and are able to indicate comorbidity of subjects for multiple disorders based on their predictive confidence level (Filippone et al., 2012). Other approaches to identify comorbidities include multi‐label classification (M.‐L. Zhang & Zhou, 2014) and multi‐task learning (Pan & Yang, 2010), where multiple class labels are assigned to each sample.
- *Patient screening*: Before proceeding with clinical procedures or expert opinions, neuroimaging‐based predictive outcomes can be utilized as a patient screening phase. A prior screening of patients using predictive modeling might reduce the time and expenses related to clinical interviews.
- *Treatment response/outcome prediction*: In addition to aiding the decision‐making procedure in clinical diagnoses, predictive techniques can also be utilized in predicting treatment response (Bzdok & Meyer‐Lindenberg, 2018) (e.g., Gong et al., 2011) and treatment outcome (e.g., Schmaal et al., 2015). By monitoring treatment outcomes and pursuing potential treatments using predictive modeling, the clinical diagnosis can become more cost‐efficient.
- *Drug trial design*: Based on predictive modeling outcomes, future response can also be classified. By selecting subsets of individuals who are most likely to response to a particular medication, more efficient drug trails can be designed. For example, medication‐class of response to mood stabilizers (bipolar) or antidepressants (depression) can be classified using machine learning approaches (Osuch et al., 2018).

### 4.2 Prediction of continuous measures versus categorical diagnoses

Most of the mental illness prediction studies surveyed in this review are based on assignment of discrete or categorical class labels for test samples. However, the categorical diagnosis approach overlooks the continuous measures while predicting a certain disease class, which can lead to misleading outcomes, or miss sub‐clinical tendencies that can be useful for predicting risk. For more reliable outcomes, predictive modeling using continuous measure, such as pattern regression, can become a valuable tool. Moreover, for mental illness prediction using brain‐based features, regression‐based modeling can be used to estimate the disease progression and treatment outcomes, and can estimate continuous measures (e.g., neuropsychiatric or cognitive measures). In order to estimate continuous clinical measures from neuroimaging data, a recent study by Wang and colleagues proposed a framework using the relevance vector machine (RVM) to build regression and obtained higher classification accuracy and better generalizing ability compared to support vector regression (Y. Wang, Fan, Bhatt, & Davatzikos, 2010). Another study explored interregional cortical thickness correlations to identify and characterize the autism diagnostic observation schedule score in ASD (Sato et al., 2013). The results from this study showed that structural covariance measures among multiple brain networks are associated with autistic symptoms. Further, Tognin and colleagues used relevance vector regression to Predict Positive and Negative Syndrome Scale scores of subjects at high risk of psychosis based on gray matter volume and cortical thickness measurements (Tognin et al., 2014). More recently, studies have started to predict continuous measures of assessment in both health and disease (e.g., the research domain criterion [RDoC]; T. Insel et al., 2010). These studies suggest that promising results can be achieved by using continuous measure for disease prediction in addition to categorical diagnosis.

### 4.3 Prediction of disease risk (prodromal state)

While challenging, early diagnosis of individuals at high risk of future mental illness is very critical in order to delay or prevent the disease progression. Since most mental illnesses typically have an onset in adolescence or early adulthood (Kessler et al., 2005), early detection could delay, or even prevent, future onset of these severe illnesses in high‐risk adolescents. Predictive modeling based approaches offer promising tools to be used for clinical diagnosis, such as identification of neuroimaging‐based biomarker that can support early identification of potentially at‐risk individuals of developing mental disorders, with the potential risk being unidentified. However, only a limited number of studies have explored the discriminative power of predictive modeling to identify healthy adolescents genetically at‐risk for mental illnesses. Mourão‐Miranda and colleagues investigated whether Gaussian process classifiers (GPC) based machine learning algorithm using neuroimaging data could help differentiate healthy adolescents genetically at‐risk for BP disorder and other Axis I psychiatric disorders from healthy adolescents at low risk of developing these disorders, and identify those healthy genetically at‐risk adolescents who were most likely to develop future Axis I disorders (Mourão‐Miranda et al., 2012). Results from this study showed that the model can discriminate healthy low‐risk from healthy adolescents genetically at‐risk for Axis I disorders, as well as help predict which at‐risk adolescents subsequently develop these disorders. Further, Guo and colleagues used fractional amplitude of low‐frequency fluctuation (fALFF) to characterize signals from the default‐mode network in unaffected siblings of SZ patients using resting‐state functional MRI data (W. Guo, Su, et al., 2014). Also, using machine learning methods, Fan and colleagues explored structural endophenotypes in unaffected family members of SZ patients, and reported that family members had structural profiles highly overlapping with those of SZ patients (Fan et al., 2008).

### 4.4 Prediction of disease onset and treatment outcome/responses

Typically, prediction approach is applied for predicting disease onset, progression, diagnosis, or treatment outcome/response. For more clinical context, predictive modeling can be used as a diagnostic tool or for predicting treatment response. In order to predict the onset of MDD, a recent machine learning‐based longitudinal, 5‐year follow‐up study was performed (Foland‐Ross et al., 2015), where cortical thickness measures were used to predict differences between participants who later experienced depression and subjects who remained healthy, with a predictive accuracy of 70% (*p* = .021). A few studies have attempted to predict the treatment response of mental disorders. Using machine learning approach, Liu and colleagues obtained an accuracy of 82.9% to predict treatment response and resistance (F. Liu et al., 2012). Another study by Osuch and colleagues examined the translational perspective of fMRI (i.e., spatially independent components [ICs]) based classification for medication‐class of response prediction in patients with MDD and BP (Osuch et al., 2018). Using SVM classifiers and a nested CV approach, the algorithm predicted the medication class of response 91.7% of the time. To prevent or delay disease progression, as well as to develop optimized treatment plans, further applications of machine learning‐based disease onset and treatment response prediction are required.

## 5 CURRENT TRENDS IN THE BRAIN‐BASED PREDICTOME

### 5.1 Univariate versus multivariate approaches

The recent methodological developments of a wide range of multivariate neuroimaging analysis approaches have received growing attention due to their ability to examine additional features beyond the voxel‐wise, univariate techniques. In case of univariate, functional neuroimaging approaches, typically a model of the expected response variable is fitted independently at each voxel's time course, and then further tests are performed using the estimated response levels during experimental conditions (K. J. Friston et al., 1994). Although convenient, this approach introduces a large number of statistical tests as it results in statistical difference maps of responses by targeting only selective brain locations related to a specific stimulus (i.e., voxels or ROIs; K. J. Friston et al., 1994). Thus, it limits the study of stimulus‐independent relationships among brain regions (e.g., functional connectivity). In addition, the univariate approach does not allow estimation of the stimulus effects at multiple brain locations. Multivariate neuroimaging approaches take into account the full spatial pattern of brain activity simultaneously at multiple spatial locations, and are able to detect subtle but localized measures of brain activity that are not captured by univariate approaches (Allen et al., 2011; Habeck et al., 2008; Habeck et al., 2005; J. Liu & Calhoun, 2014; McIntosh & Lobaugh, 2004; Moeller & Strother, 1991; Narayanan et al., 2015; Sui, Adali, Yu, Chen, & Calhoun, 2012). In contrast to the traditional univariate, model‐based approach which lacks the ability to directly address interaction between voxels/regions, multivariate approach estimates correlation or covariance of activation across brain regions. Multivariate results can also be more reliably translated as a signature of underlying brain networks. Recent multivariate neuroimaging methods offer to analyze the relationship between a stimulus and the responses simultaneously measured at many locations, such as spatial response patterns or multi‐voxel response patterns. Compared to univariate techniques, multivariate approaches provide potentially greater statistical power and better reproducibility.

**FIGURE 7:**
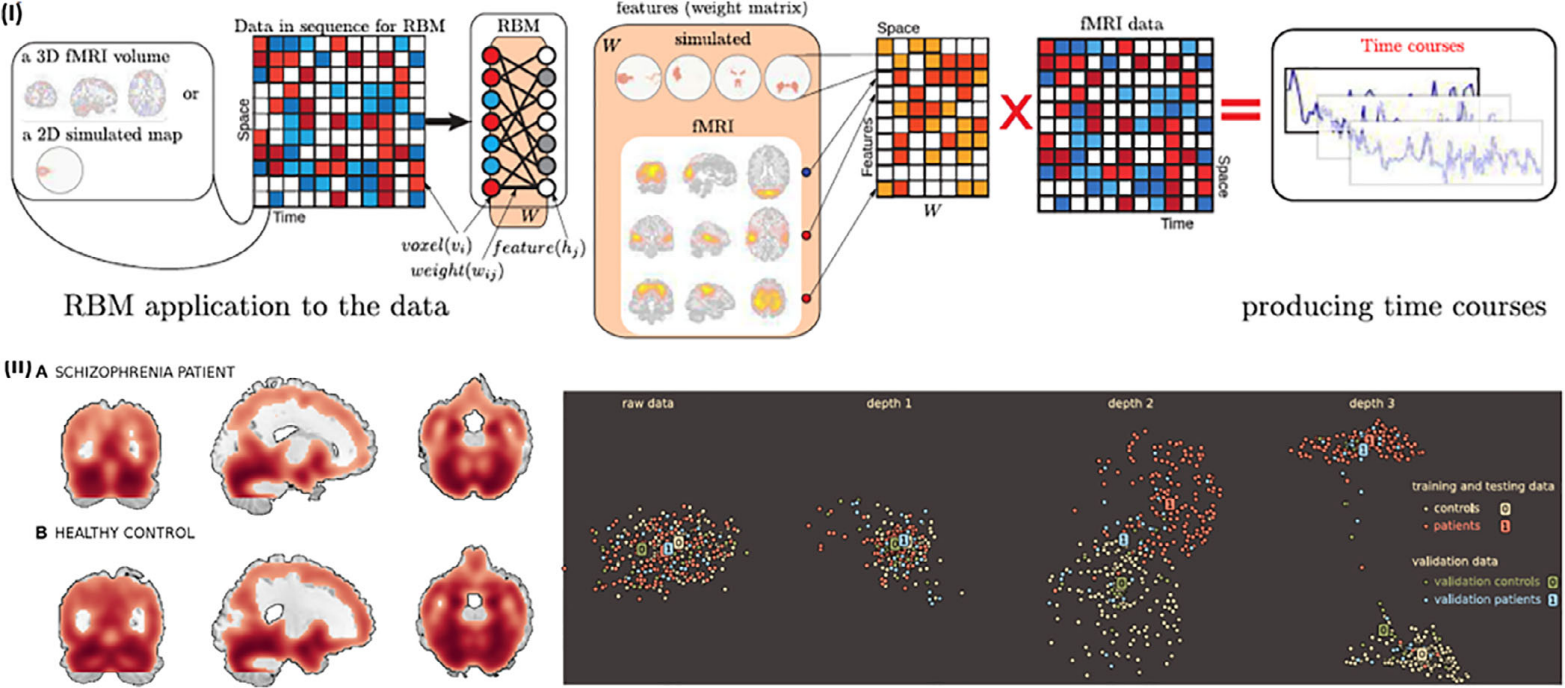
A deep‐learning approach for schizophrenia prediction. (I) Methodological illustration of restricted Boltzmann machine (RBM) based deep learning pipeline. Features were learned from the time‐courses of the data. (II) Example showing a smoothed gray matter segmentation of a training sample of a schizophrenia patient and a healthy control (left), and the effect of a deep belief network's (DBN) depth on neighborhood relations (right). Results showing that after Depth 1 and Depth 2, the DBN continues distilling details that pull the classes further apart. Figures reprinted with permission from Plis et al. (2014)

In neuroimaging‐based diagnoses, multivariate machine learning methods integrate available features simultaneously to jointly differentiate between groups. Typically, for a multivariate machine learning approach, a classifier is trained on a training dataset to predict different classes (e.g., patient groups), which is then applied to the testing dataset. The classification accuracy (i.e., correct classification rate) is then estimated, using a CV procedure for improved accuracy. To date, a number of multivariate machine learning methods have been applied in neuroimaging‐based prediction, such as SVM, k‐nearest neighbor (KNN), Gaussian Naive Bayes (GNB), and LDA. Among these classifiers, the SVM‐based classifier has been reported to obtain greater classification performance (Davatzikos et al., 2005; Mitchell et al., 2004; Mourao‐Miranda, Bokde, Born, Hampel, & Stetter, 2005; Wang, Hutchinson, & Mitchell, 2004). Other recent and more efficient classifiers include: The Random Forest (Breiman, 2001), deep learning (Calhoun & Sui, 2016; Han et al., 2017; Jang et al., 2017; Plis et al., 2014; Figure 7) and artificial neural network (ANN; H. Guo et al., 2014; J. Kim et al., 2016) classifiers.

Example of multivariate machine learning methods for mental illness diagnoses includes classification of SZ using structural MRI data with classification accuracies ranging from 81% to 93% (Gould et al., 2014; Greenstein et al., 2012; Kawasaki et al., 2007; Sun et al., 2009; Yoon et al., 2008).

### 5.2 Multimodal studies

Although neuroimaging techniques have become popular tools to identify mental illness related biomarkers, each imaging technique has its limitations (Calhoun & Adali, 2009; Calhoun & Sui, 2016; S. Liu, Cai, et al., 2015). The modality‐specific limitations can be partly overcome by developing multimodal neuroimaging techniques, by combining data obtained from multiple neuroimaging techniques, such as EEG, structural magnetic resonance imaging (sMRI), and fMRI, which provides more informative and reliable results on brain structure and dynamics than unimodal neuroimaging approach. Multimodal neuroimaging is a relatively new and rapidly expanding field that integrates data from different modalities to understand the pathophysiology of mental illness (Calhoun et al., 2006; Sui et al., 2012). For instance, by linking the genomic variation to brain function, structure and connectivity measures, imaging genomics approach can characterize the underlying neural mechanisms through which genomics variations affect cognition and behavior in mental illness. Multimodal data fusion approaches utilize complementary information available from both data modalities to jointly estimate their association by accounting for inter‐modality relationships. Particularly, for complex disorders such as SZ, the relationship between features from multiple modalities may be better understood by incorporating additional information provided by advanced multimodal modeling. Thus, it is important to study the inter‐relationships between multimodal neuroimaging data, as well as nonimaging features, such as, behavioral and genomic data, while performing predictive modeling of mental illness.

There are currently multiple approaches to integrate data acquired from various unimodal imaging techniques, as described by Calhoun and Sui (2016). These include a separate visual inspection of unimodal results, multimodal integration after individually analyzing unimodal results to prevent any interaction between the unimodal data, and data fusion using either asymmetric approach where one modality constrains the other (e.g., fMRI constrained EEG) or a symmetric approach where multiple modalities are analyzed jointly (e.g., joint independent component analysis [joint‐ICA] or parallel independent component analysis [parallel‐ICA]).

While most of the studies in the existing literature used a single modality for mental illness prediction, recent development in data fusion approaches have made multimodal neuroimaging a popular trend. Several recent studies have differentiated SZ patients from HC by combining data from rs‐fMRI or task‐based fMRI, and sMRI (Cabral et al., 2016; J. Ford, Shen, Makedon, Flashman, & Saykin, 2002; Qureshi, Oh, Cho, Jo, & Lee, 2017; Yang, He, & Zhong, 2016), fMRI and single nucleotide polymorphism (SNP; genomic feature; Yang et al., 2010), and rs‐fMRI and MEG (Cetin et al., 2016), while only a few studies combined data from three or more modalities (Sui et al., 2013; Sui et al., 2014), with accuracy ranging from 75% to 100%. Other recent data fusion advances include integration of multiple task‐based fMRI data sets (D. I. Kim et al., 2010; Sui, Adali, Pearlson, & Calhoun, 2009; Sui et al., 2015) from the same participants, where common versus specific sources of activity was specified to a greater degree than conventional general linear model‐based approaches. Using a Fisher's linear discriminate classifier, Ford and colleagues classified SZ and HC based on tasked‐based fMRI data with 78% accuracy, and sMRI data with 52% accuracy, however, the combined multimodal data (fMRI and sMRI) resulted in the highest accuracy of 87% (J. Ford et al., 2002). Another recent multimodal neuroimaging study by Yang and colleagues integrated rs‐fMRI based connectivity features and sMRI based structural features extracted using ICA, and used SVM classifier to compare unimodal versus multimodal accuracy (Yang, Chen, et al., 2016). Results from this study showed that multimodal features achieved higher accuracy (77.91%) than single modality accuracy (72.09%). Using multimodal sMRI and rs‐fMRI data, Cabral and colleagues classified SZ patients and HC individuals with 75% accuracy, where the multimodal features based classification outperformed both of the unimodal features based prediction accuracy (69.7% accuracy using sMRI data and 70.5% accuracy using rs‐fMRI data; Cabral et al., 2016). Qureshi and colleagues employed a similar approach to classify SZ patients and HC individuals using combined rs‐fMRI and sMRI data but on a higher number of samples, and achieved 10‐by‐10‐fold nested cross‐validated prediction accuracy of 99.29% (Qureshi, Oh, Cho, et al., 2017). Note that, in order to use as much training data as possible and overcome the sample size issue, the framework utilized a nested CV without novel data for testing, which might have introduced classification bias and resulted in such high prediction accuracy. Regardless of the methodological limitations, these and other studies show the potential of leveraging multimodal imaging data. However, more robust multimodal fusion approaches and validations are required before making them available for clinical purposes.

**FIGURE 8:**
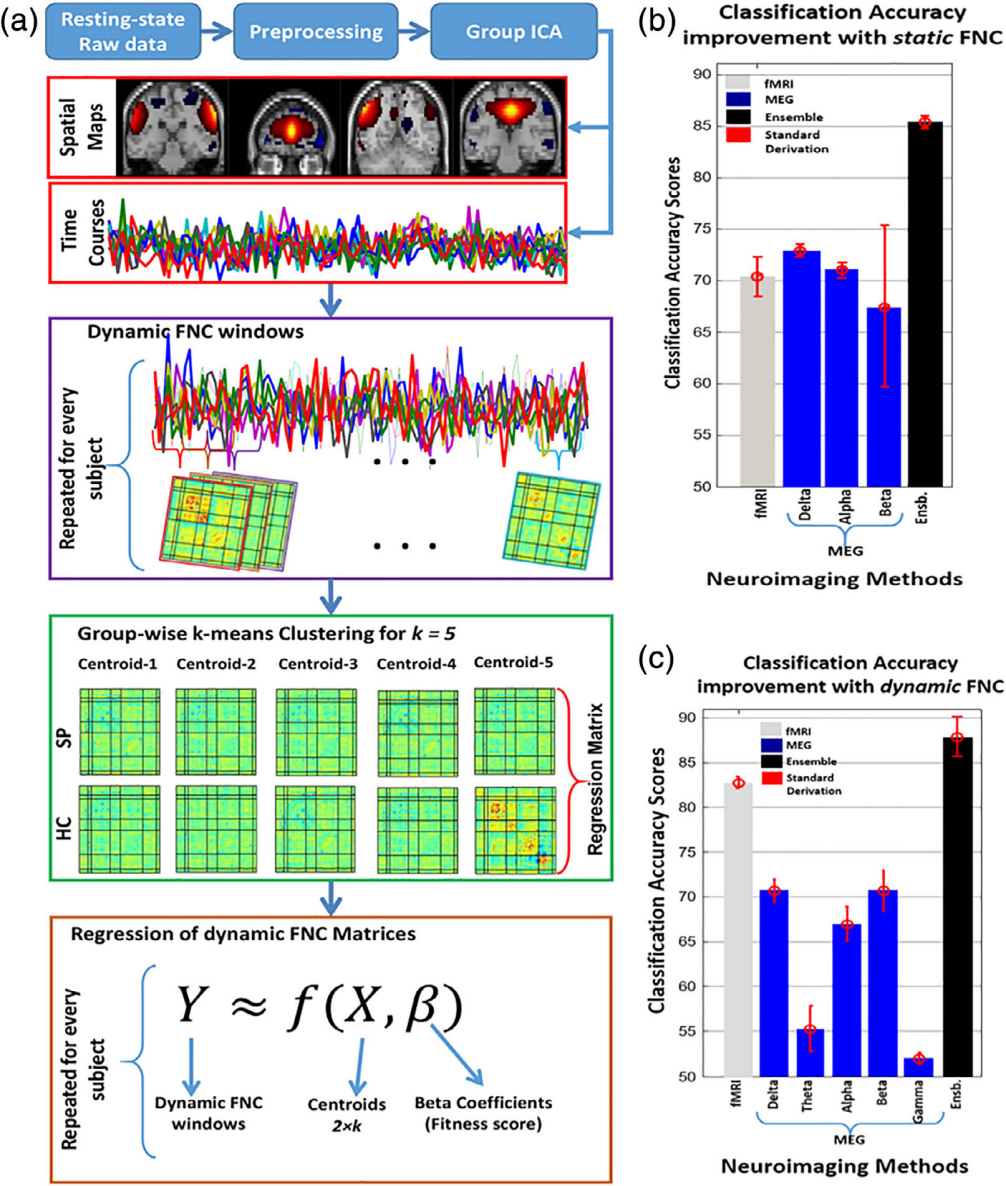
Schematic description showing the multimodal, MEG‐fMRI classification framework. (a) Both resting‐state fMRI and MEG data went through group ICA, windowed‐FNC and k‐means clustering approach. Regression analyses were performed on dynamic FNC measures to extract features. (b) Bar plots showing average classification accuracy improvement with static FNC approach. (c) Bar plots showing average classification accuracy improvement with dynamic FNC approach. Dynamic approach clearly outperformed static FNC approach. Figures reused with permission from Cetin et al. (2016)

Besides multimodal integration of MRI modalities, recent studies have also combined neuroimaging data with non‐neuroimaging features, such as genomics data (e.g., SNPs), DTI, MEG, and EEG for classification of mental illnesses. Using ICA and SVM‐based classifier ensemble (SVME), in a relatively small sample size and SNPs array, Yang and colleagues classified SZ patients and HC with 73.88% accuracy for SNPs data, 81.63% accuracy for voxel‐level fMRI activations, 82.50% accuracy for ICA component‐specific fMRI activation, and finally, 87.25% accuracy for combined fMRI‐SNPs data (Honghui Yang et al., 2010). Further, in a large dataset, using multiple classifiers including sparse representation‐based classifier (SRC), fuzzy c‐means (FCM) classifier, and SVM‐based classifier, Cao and colleagues discriminated between SZ patients and HC individuals by combining fMRI and SNP data modalities, and found the best classification accuracy of 89.7% achieved using SRC (L. Cao et al., 2014). Another recent study by Cetin and colleagues integrated rs‐fMRI and MEG data to distinguish SZ from HC, and found that the best performance of 87.91% accuracy was obtained by using the ensemble classifier (Cetin et al., 2016; Figure 8). Using a novel data fusion technique known as mCCA + jICA and multiple types of classifiers, Sui and colleagues integrated features from rs‐fMRI, sMRI, and DTI (i.e., FA) to classify SZ patients and HC individuals, and achieved a maximum classification accuracy of over 90% using radial basis function support vector machine (RSVM) classifier on DTI (i.e., FA) and sMRI (gray matter) features (Sui, He, Pearlson, et al., 2013). Further, using the sample data fusion technique (i.e., mCCA+jICA) and features from rs‐fMRI, sMRI, and EEG modalities, Sui and colleagues utilized a SVM classifier with recursive feature elimination (SVM‐RFE), and obtained 91% accuracy in training data and 100% accuracy (i.e., predication rate) with combination of all modalities to classify SZ patients and HC individuals (Sui et al., 2014). In order to classify ultra‐high‐risk individuals for psychosis, first‐episode psychosis and HC, Pettersson‐Yeo and colleagues used a multi‐step data fusion approach that includes an unweighted sum of kernels, multi‐kernel learning, prediction averaging, and majority voting, and obtained 86.33% accuracy by combining features from DTI and fMRI modalities (Pettersson‐Yeo et al., 2014).

The results of the above‐mentioned studies are encouraging for using multimodal neuroimaging in classification of mental illness, suggesting that data fusion methods combined with advanced machine learning techniques present a promising direction for mental illness prediction.

### 5.3 Multi‐class classification for disease subtype to reduce diagnoses heterogeneity

While the case (i.e., patient with mental illness) versus control diagnostic approach has been successfully implemented in the existing mental illness prediction literature, it does not address the differential diagnosis aspect of mental illness prediction (i.e., distinguishing between illnesses with overlapping symptoms or subgroup diagnosis). Traditional case versus control model ignores the heterogeneity within the disease group by assigning predefined case or control label to the test sample. Many severe mental illnesses, such as SZ, schizoaffective disorder, unipolar and bipolar depression, and other mood disorders share substantial overlapping symptoms, and therefore disease prediction based on the reported symptoms alone is not adequate for accurate diagnosis. To address this, the National Institute of Mental Health introduced the research domain criteria (RDoC; http://www.nimh.nih.gov/research-funding/rdoc), as a way to classify mental illnesses based on multiple symptom, behavioral and biological dimensions, with a goal of reducing heterogeneity across diagnostic groups (T. Insel et al., 2010; T. R. Insel, 2014). Consistent with the European roadmap for mental health research (Schumann et al., 2014), the RDoC offers an improved classification validation at the neurobiological level. Most of the studies addressing the RDoC initiative have implemented various data‐driven, clustering approaches based on neuropsychological measures in order to divide the clinical populations. These studies include subtype diagnoses of SZ (Bell, Corbera, Johannesen, Fiszdon, & Wexler, 2011; Brodersen et al., 2014), mood disorders (Lamers et al., 2010; Van Loo, De Jonge, Romeijn, Kessler, & Schoevers, 2012), and ADHD (Dias et al., 2015; D. A. Fair, Bathula, Nikolas, & Nigg, 2012; Koutsouleris et al., 2015; van Hulst, De Zeeuw, & Durston, 2015).

As discussed above, one of the main limitations of the traditional case–control prediction is the binary disease characterization, where test samples are assigned to either case or control category. This approach overlooks the associated disease heterogeneity, commonly known as the disease subtype. However, many heterogeneous mental illnesses including autism and SZ are defined as spectrum disorders (i.e., a continuum) with multiple disease etiologies lying under the same diagnostic category. While it is a common practice to classify such spectrum‐like disorders using the generic category to find diagnostic biomarkers, a major issue in mental illness diagnostic procedure is the lack of differential diagnosis of patients across several disease subtypes. Accurate diagnosis of disease subtype is very critical for the appropriate course of treatments. For example, in case of SZ, patients can exhibit similar cognitive deficits but with variable magnitude. Therefore, to emphasize the phenotypic heterogeneity in SZ, two major subtypes with different genetic and cognitive profiles have been introduced: (a) cognitive deficit and (b) cognitively spared (Green et al., 2013; Jablensky, 2006). However, differential diagnosis of SZ subtypes has been rarely studied, due to limited sample size. The number of subjects in each disease subtype is small in most of the existing datasets, which limits the ability to develop robust subtype predictor to accurately differentiate them. Ingalhalikar and colleagues proposed an unsupervised spectral clustering approach using multi‐edge graphs derived from a structural connectivity network among 78 ROIs to classify subtypes of autism and SZ (Ingalhalikar et al., 2012).

**FIGURE 9:**
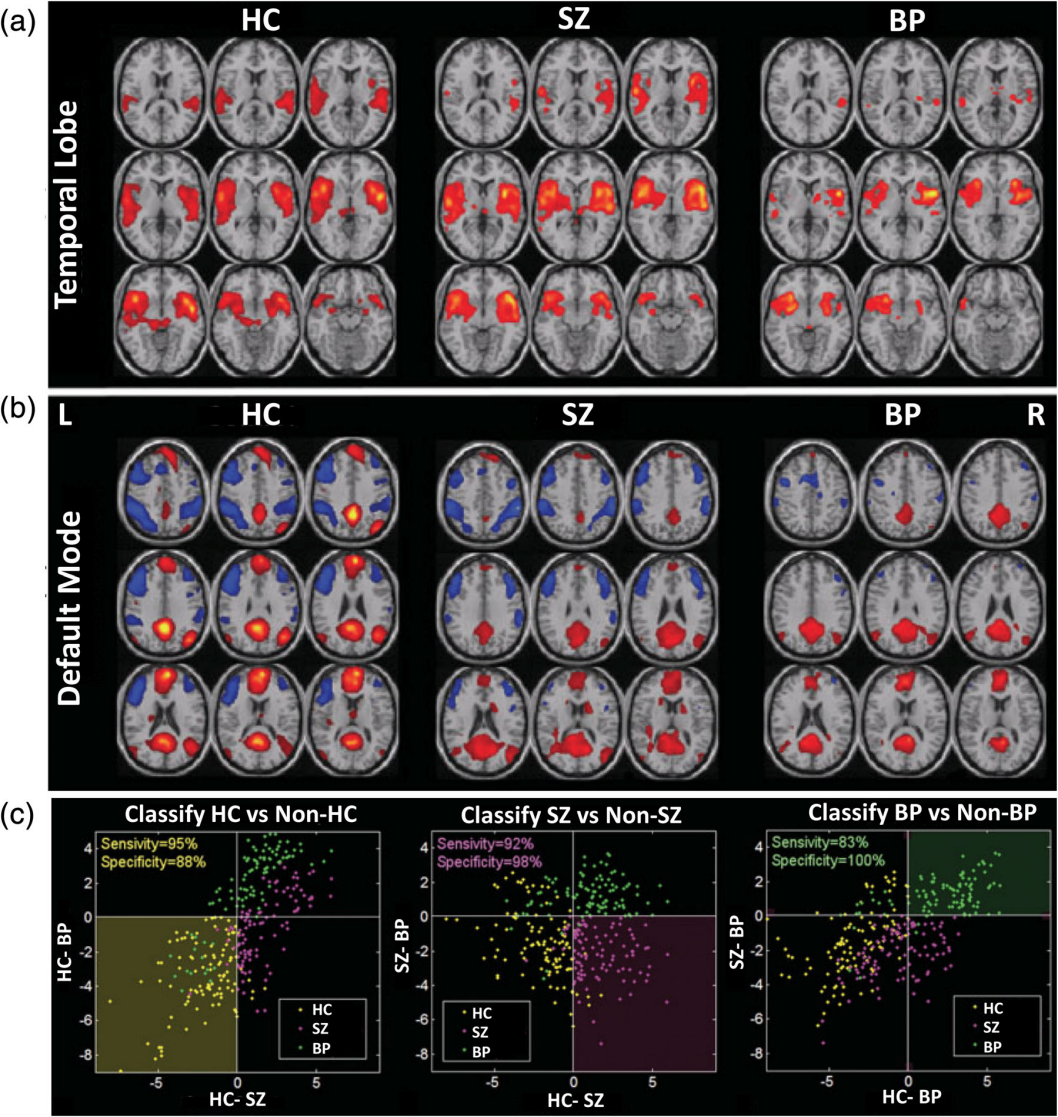
Prediction of schizophrenia (SZ) and bipolar disorder (BP) using temporal lobe and default mode components. (a) Group‐wise average temporal lobe and (b) default mode features, extracted from fMRI data from healthy control (HC), SZ, and BP patients. Components are thresholded at *p* < .001 (corrected). (c) Classification results illustrated with a priori decision regions, and actual diagnosis of test subjects. The average sensitivity and specificity were 90 and 95%, respectively. Figures modified and reprinted with permission from Calhoun et al. (2008)

**FIGURE 10:**
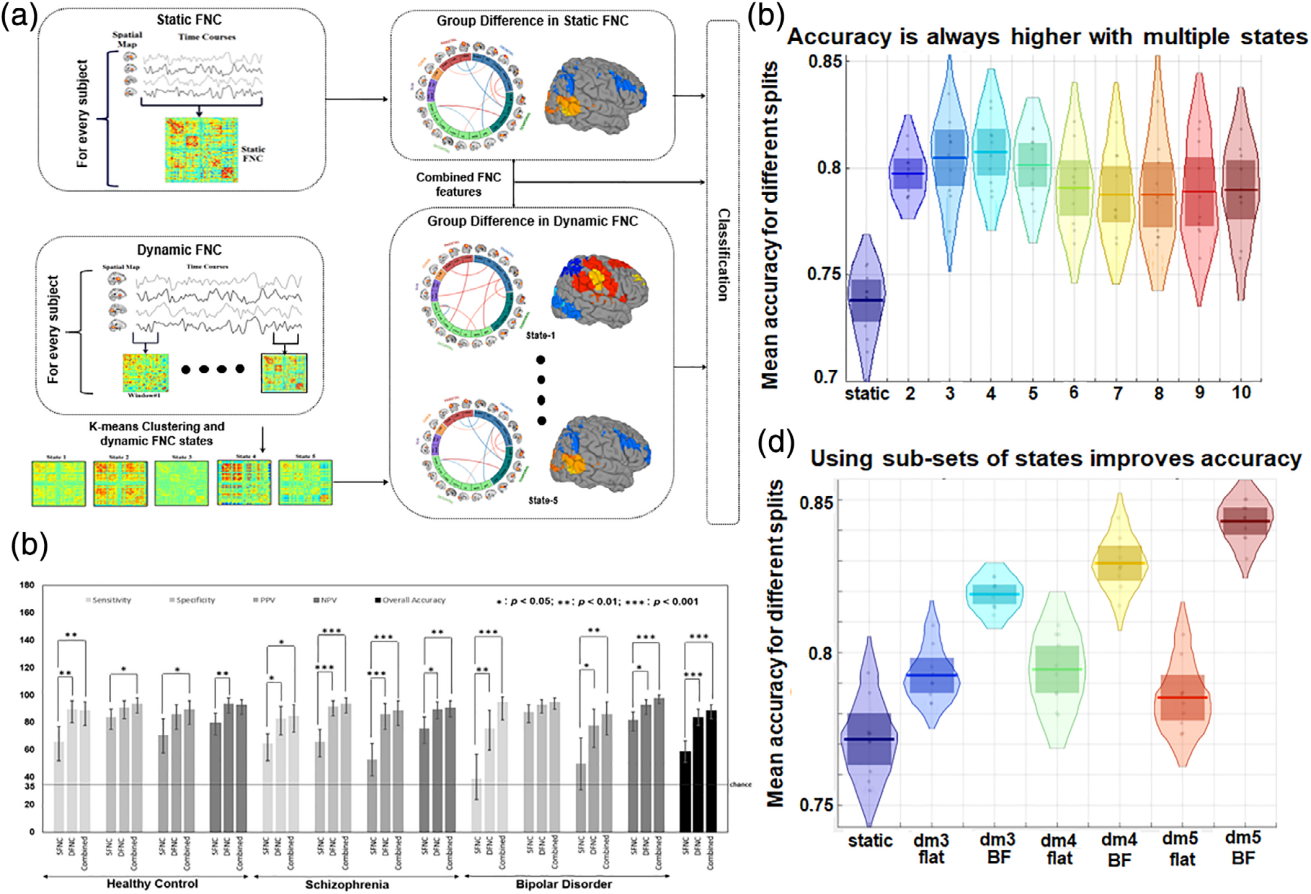
Classification approach using static and dynamic FNC measures. (a) Windowed FNC and k‐means clustering methods were used to extract dynamic FNC features from schizophrenia (SZ), bipolar (BP) and healthy control (HC). (b) Classification results showing that dynamic FNC approach outperformed static FNC framework (accuracy: 84% versus 59%, respectively). (c) Another FNC‐based approach showing dynamic FNC approach clearly outperforms static FNC approach (Saha et al., 2019, ISBI). Multiple dynamic states (*k* = 2–10) were utilized to evaluate classification performance. (d) Further, using subsets of states and flat and Brute Force (BF) approaches, performance was evaluated, showing improvement in accuracy with subsets of states (Saha et al., 2019, ohbm). Figures modified and reprinted with permission from Rashid et al. (2016), Saha, Abrol, et al. (2019), and Saha, Damaraju, et al. (2019)

Among the surveyed studies in this review, only a few considered the important area of automatic differential diagnosis. Costafreda and colleagues used fMRI with a verbal fluency task for subject‐level classification of SZ, bipolar and HCs (Costafreda et al., 2011). Two studies by Calhoun et al. (2008) (Figure 9) and Arribas et al. (2010) used fMRI with an auditory oddball task where they have applied an ICA approach to extract the default model network related features and the temporal lobe of the brain. Both of these studies achieved high prediction accuracy between SZ and BP disorder. Rashid and colleagues proposed static dynamic functional network connectivity (FNC) features‐based algorithms (Figure 10) to automatically classify SZ, bipolar and HCs (Rashid et al., 2016). Another study by Pardo and colleagues used a combination of volumes of 23 ROIs derived from structural MRI along with 22 neurophysiological test scores for automatic classification of SZ, bipolar and HCs (Pardo et al., 2006). More recently, using gray matter densities, Schnack and colleagues proposed a classification framework for SZ, bipolar and HCs (Schnack et al., 2014). Using gray matter maps from structural MRI, Koutsouleris and colleagues classified SZ from mood disorder (Koutsouleris et al., 2015). Ota and colleagues combined volumetric measures derived from structural MRI with FA from dMRI in selected ROIs to classify SZ from MDD (Ota et al., 2013). Moreover, using gray matter volumes of caudate and ventral diencephalon, Sacchet, Livermore, et al. (2015) proposed an algorithm to classify MDD, bipolar and remitted MDD patients.

Although the limited sample size in most of the current datasets makes it challenging to perform disease subtype prediction as the number of subjects in each diseases subtype is small, it shows the potential for a paradigm shift in the predictive modeling of spectrum‐like mental illness beyond discrete, case–control diagnosis.

### 5.4 Advanced algorithms for brain‐based prediction

**FIGURE 11:**
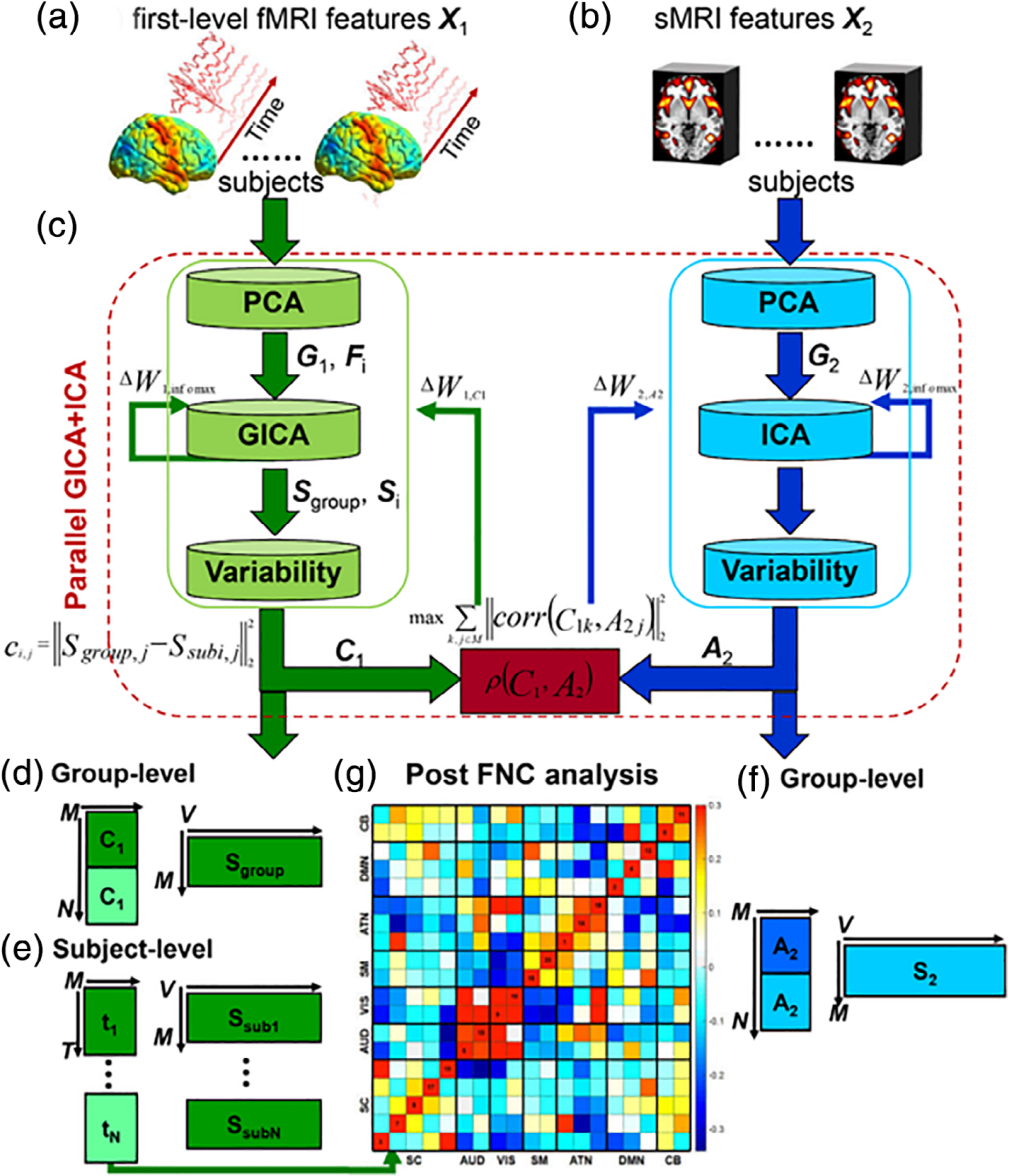
A joint estimation approach for schizophrenia prediction using parallel group ICA + ICA. Flowchart showing steps to extract first‐level fMRI (a) and sMRI (b) features, feature integration using parallel GICA+ICA (c), group‐level components extraction from GICA (d), subject‐level GICA components extraction (e), group‐level GICA components extraction (f), FNC analysis. Figures reprinted with permission from Qi et al. (2019)

**FIGURE 12:**
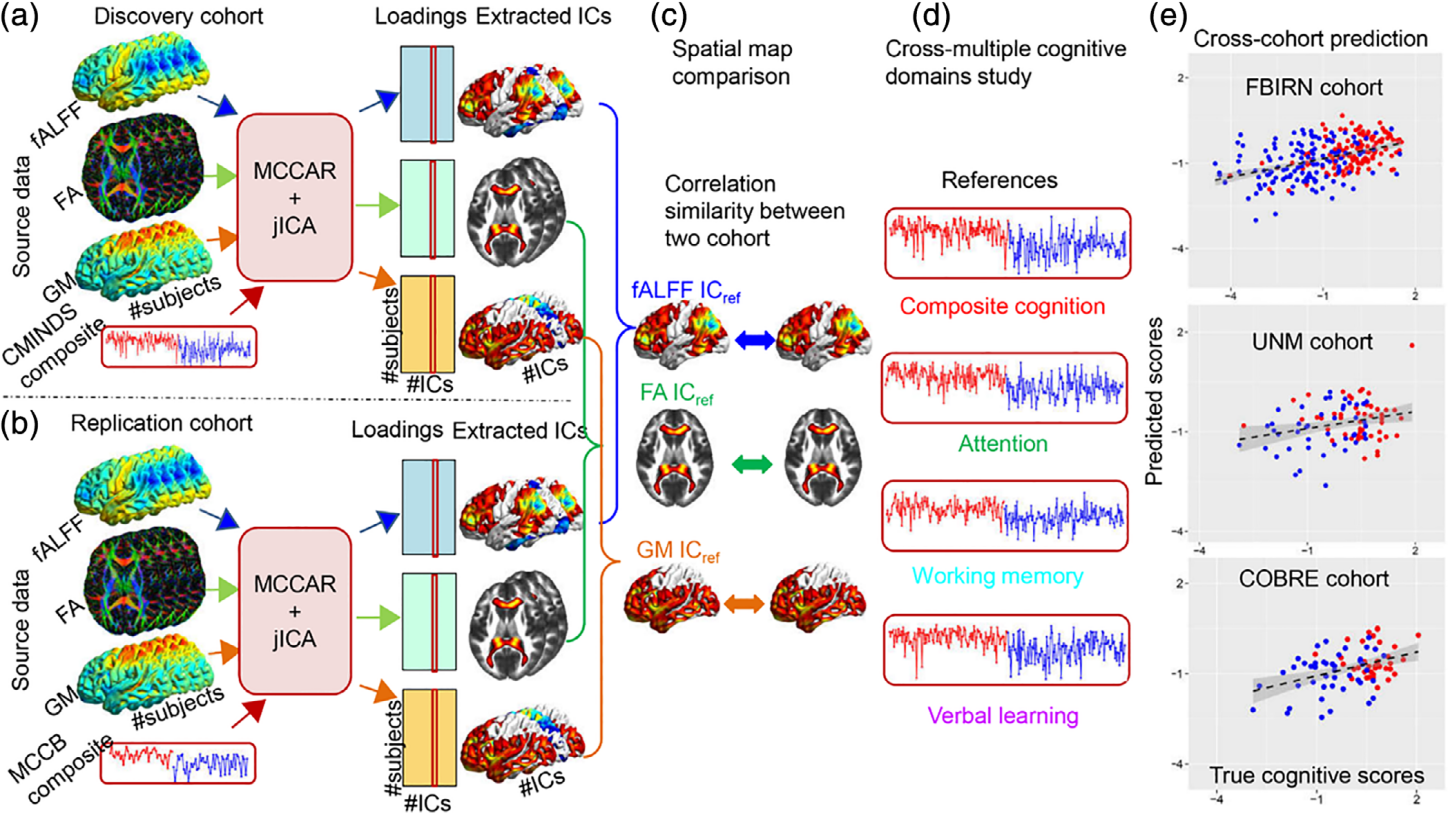
Predicting cognition. An approach to predict cognition with constrained data fusion. Comparison of composite cognition associated with multimodal covarying patterns between multiple cohorts. The composite cognitive scores were used as references for multisite data cohorts. Figures reprinted with permission from Sui et al. (2018)

Recent advanced machine learning algorithms have shown tremendous potential for neuroimaging‐based mental illness prediction. For example, a recent study proposed a novel parallel group ICA‐based framework to jointly estimate the association between functional network variability and structural covariation in SZ, as well as to predict several cognitive domain scores based on these associated functional/structural features (Qi et al., 2019; Figure 11). Briefly, by jointly incorporating and estimating temporal domain features from fMRI (extracted from group ICA) and structural MRI features within a parallel group ICA algorithm, functional network variability and structural covariation were jointly estimated to identify between‐modality linkage. Using real neuroimaging data, a significant functional and structural MRI component pair was identified that captured group difference in both imaging modalities, which further correlated with cognitive scores suggesting that multimodal brain features can predict multiple cognitive scores. Another recent SZ study proposed a multimodal fusion with reference algorithm by combining multi‐site canonical correlation analysis with reference and joint‐ICA (MCCAR+jICA) to identify co‐varying multimodal feature patterns using a reference (specifically, working memory performance) in a three‐way data fusion (fMRI, sMRI, and dMRI; Qi et al., 2018). Results identified several brain regions that were previously linked with working memory deficits in SZ, suggesting that the novel MCCAR+jICA method has great potentials to identify biomarkers for severe mental disorders, such as SZ. Further, Sui and colleagues implemented a constrained fusion approach to predict cognition in SZ (Sui et al., 2015; Figure 12). The assessment of cognition was measured using the MATRICS Consensus Cognitive Battery (MCCB), and using multi‐set canonical correlation, the linkage between MCCB and brain abnormalities as measured by fractional amplitude of low‐frequency fluctuations (fALFF) from resting fMRI, gray matter density (GM) from structural MRI, and FA from dMRI were explored. Findings from this study suggested that the associated functional and structural deficits might be linked to cognitive impairments in SZ. Other recent neuroimaging‐based data‐driven advanced algorithms include biclustering and triclustering ICA approaches that utilize spatial and temporal variance as a measure to cluster mental disorders into homogeneous subgroups. For instance, using gray matter concentration (GMC) from SZ patients, Gupta and colleagues implemented source‐based morphometry (SBM) decomposition, followed by subtype component reconstruction using group information‐guided ICA (GIG‐ICA), and identified two subtypes (i.e., two different subsets of subjects; Gupta et al., 2017). Also, Rahman and colleagues have used structural MRI features from SZ patients to perform multi‐component and symptom bi‐clustering (Rahaman et al., 2019), and further extended this approach into tri‐clustering framework using dynamic FNC measures to identify disease subtypes (Rahaman, Damaraju, & Calhoun, 2019).

### 5.5 Functional connectivity measures for brain‐based prediction

In recent years, brain connectivity studies using neuroimaging have become popular to investigate the associations among brain networks. Functional connectivity (FC) can be quantified using a variety of different neuroimaging techniques. A commonly used measure is fMRI, which measures synchronized brain activity via blood oxygenation and infers functional interactions among different brain regions (Craddock et al., 2013). FC, defined as temporal correlation (or other types of statistical dependency) among spatially distant brain regions (K. Friston, 2002), has recently been used to examine the functional organization and temporal dependencies among these remote brain regions. Different analytic tools have been applied to resting‐state fMRI data to describe brain functional connectivity. Two widely used FC approaches are (a) seed‐based analysis (B. Biswal, Zerrin Yetkin, Haughton, & Hyde, 1995; Greicius, Krasnow, Reiss, & Menon, 2003) and (b) purely data‐driven methods, such as ICA (Calhoun & Adali, 2012; Calhoun, Adali, Pearlson, & Pekar, 2001a; Calhoun, Eichele, & Pearlson, 2009; Fox & Raichle, 2007). FC can also be investigated at the network level using spatial ICA, and connectivity among spatial components is referred to as FNC (Jafri, Pearlson, Stevens, & Calhoun, 2008). In most common cases, FC (as well as FNC) is considered to be stationary over the entire scanning period, formally known as the sFC/sFNC analysis.

**FIGURE 13:**
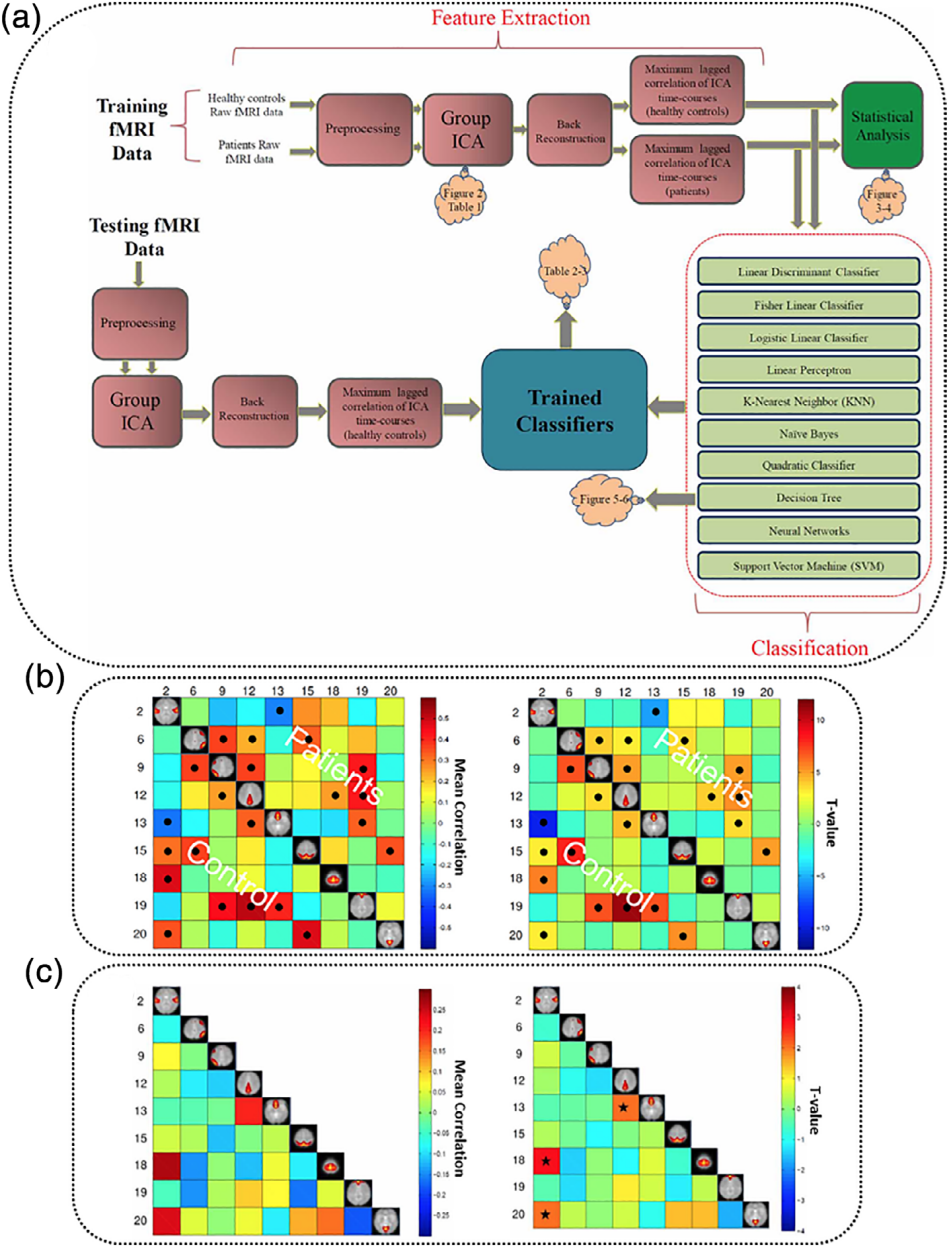
An approach to classify schizophrenia (SZ) using resting‐state functional network connectivity (FNC) measures. (a) Feature extraction and classification steps of the FNC‐based framework. (b) group‐wise mean FNC (left), and *T* values (FDR corrected, *p* < .05) (right). (c) Group difference in mean FNC measures (left), and FDR corrected (*p* < .05) *T* values showing group difference. Figures reprinted with permission from Arbabshirani et al. (2013)

For predictive analyses, specific methods for measuring functional connectivity might influence the features of interest as well as the classification accuracy. Given the high dimensional features resulting from FC analysis, without efficient but meaningful feature selection, the classifier could introduce overfitting problem, leading to poor classification performance. Thus, to eliminate redundant features and choose only appropriate features from FC measures, application of suitable feature selection strategies is critical. For FC based feature selection, approaches such as filter (e.g., statistical test, Fisher score and correlation coefficient; Figure 13; J. S. Anderson et al., 2011; Arbabshirani et al., 2013; Bassett et al., 2012; Calhoun et al., 2008; W. Du et al., 2012; Guyon & Elisseeff, 2003), wrapper (e.g., recursive feature elimination [RFE], sequential feature selection and genetic algorithm; Fan et al., 2011; Guyon & Elisseeff, 2003; Venkataraman et al., 2012; Yu, Shen, Zhang, et al., 2013), and embedded (e.g., LASSO regularization, decision tree; Fonti & Belitser, 2017; B. Jie et al., 2014; Lal, Chapelle, Weston, & Elisseeff, 2006; Watanabe et al., 2014) methods can be applied.

**FIGURE 14:**
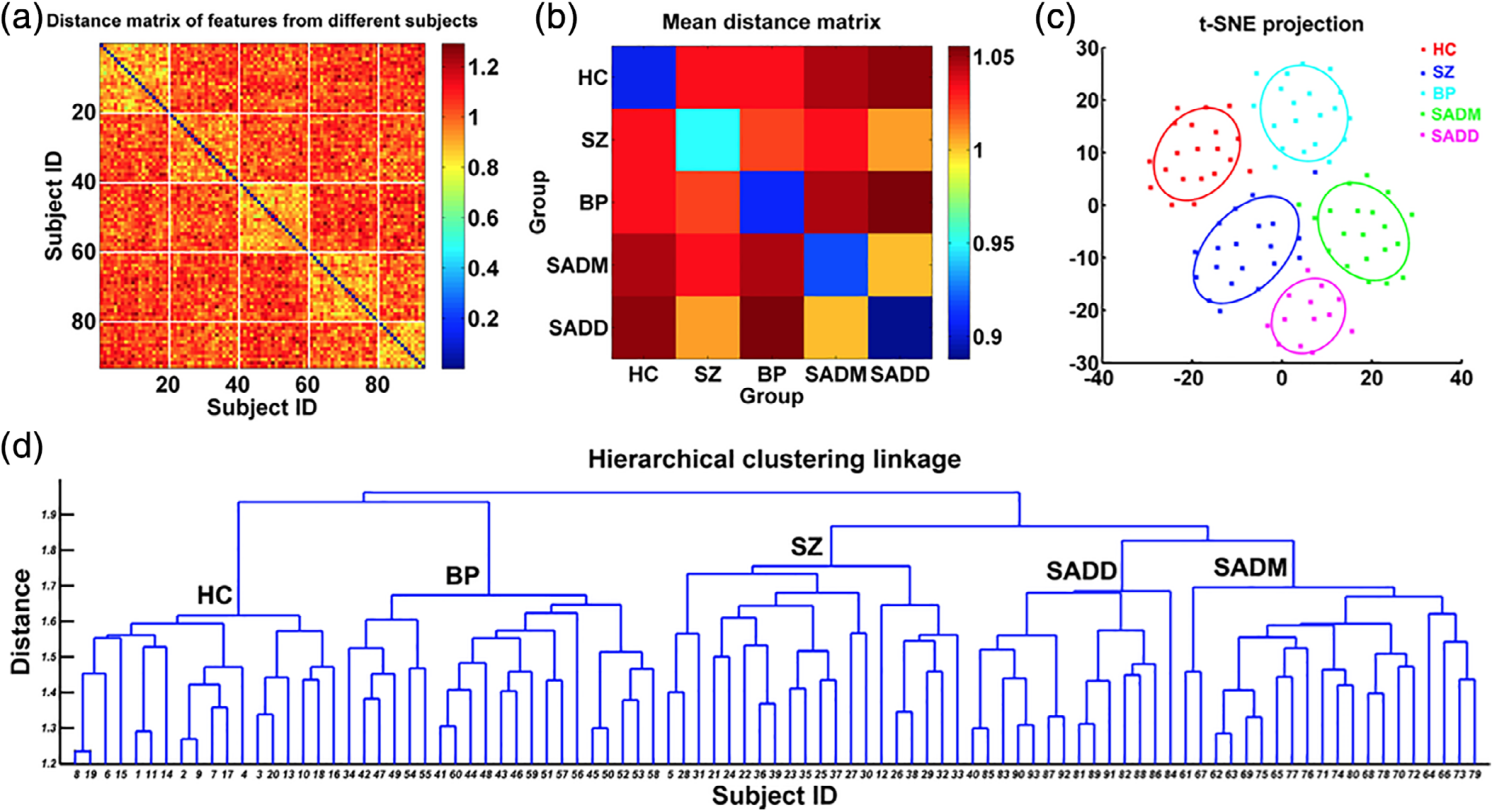
An example showing prediction of schizophrenia and bipolar using network measures and hierarchical clustering. (a) Distance matrix from the feature vectors. (b) The mean inter‐group and intra‐group distance matrix. (c) The Results from t‐distributed stochastic neighbor embedding (t‐SNE) method showing the projection results of subjects, where each point refers to a subject (group‐wise colored). (d) The linkage results from the hierarchical clustering method. BP, bipolar; HC, healthy control; SADD: schizoaffective (depression); SADM, schizoaffective (manic); SZ, schizophrenia. Figure reprinted with permission from Du et al. (2015)

Table 9 presents FNC‐based classification studies for automatic diagnoses of SZ, MDD/BP, ASD, and ADHD patients. Both FC and FNC measures have been utilized for subject‐level diagnosis (i.e., prediction) of severe mental disorders, such as SZ and bipolar. For example, by using the connectivity between functional brain networks (i.e., FNC) identified by ICA several recent studies have classified SZ (80% accuracy (A. Anderson & Cohen, 2013) 63–96% accuracy (Arbabshirani et al., 2013); 84.4% accuracy (Kaufmann et al., 2015). Another popular estimate for FC between ROIs identified based on different atlases has also been used to distinguish between SZ and healthy individuals (75% accuracy (Venkataraman et al., 2012); 82.8% accuracy (Su et al., 2013); 80.9% accuracy (Yu, Shen, Zeng, et al., 2013), 62% accuracy (Yu, Shen, Zhang, et al., 2013); 59.7–90.8% accuracy (Watanabe et al., 2014); 86% accuracy (J. Kim et al., 2016)). Further, Bassett et al. (2012) have use high‐level network organization as features for classification of SZ (75% accuracy). In addition to the single modality based FNC features, studies have also explored the fusion of FC with features from other modalities to classify SZ. For example, Yang and colleagues developed a hybrid machine learning approach using fMRI (voxels and ICA networks) and SNP features for classification of SZ and achieved 87% classification accuracy using the combined features (i.e., voxel, ICA network, and SNP data; Yang et al., 2010). Recent studies have also classified SZ and BP disorder using FC‐based features. Using ICA spatial maps of the temporal lobe and the default mode networks, Calhoun and colleagues classified SZ and bipolar patients with accuracies of 83–95% (Calhoun et al., 2008). Another study used the ICA spatial maps as features and a probabilistic Bayesian classifier to discriminate between SZ, BP disorder, and healthy individuals, and achieved the average three‐way correct classification rate within the range of 70–72% (Arribas et al., 2010). Few recent studies also performed of classification of mental illnesses with overlapping symptoms, such as SZ, schizoaffective disorder, and BP disorder with psychosis (Cardno & Owen, 2014; Cosgrove & Suppes, 2013; Pearlson et al., 2016). Du and colleagues used GIG‐ICA to extract resting‐state brain networks and classified SZ patients, BP disorder with psychosis, schizoaffective disorder with manic episode disorders schizoaffective disorder with depressive episodes exclusively and healthy individuals (Du et al., 2015). FNC features were selected using RFE method and a five‐class SVM classifier was used for training purpose, achieving 68.75% of classification accuracy (Figure 14).

**TABLE 9:** Functional network connectivity

References	Disorder	Number of subjects	Feature type	Classifier	Overall accuracy
Calhoun et al. (2008)	SZ/BP	HC = 26, SZ = 21, BP = 14, Total = 61	Network maps (DMN and temporal lobe) extracted by ICA	ED	83–95%
Demirci, Clark, and Calhoun (2008)	SZ	HC = 36, SZ = 34, Total = 70	Network maps extracted by ICA	Projection pursuit	80–90%
Yang et al. (2010)	SZ	HC = 20, SZ = 20, Total = 40	Multiple features include SNP, voxels in fMRI map, and components of fMRI activation	SVM	74–87%
Castro et al. (2011)	SZ	HC = 54, SZ = 52, Total = 106	Activation maps extracted by GLM and networks extracted by ICA	SVM	95%
Fan et al. (2011)	SZ	HC = 31, SZ = 31, Total = 62	Network maps extracted by ICA	SVM	87%
Bassett et al. (2012)	SZ	HC = 29, SZ = 29, Total = 58	FC between 90 ROIs (AAL atlas)	SVM	75%
W. Du et al. (2012)	SZ	HC = 28, SZ = 28, Total = 56	Network maps extracted by ICA	Fisher discriminant function	93–98%
Tang et al. (2012)	SZ	HC = 22, SZ = 22, Total = 44	FC between 90 ROIs (AAL atlas)	SVM	93.20%
Venkataraman et al. (2012)	SZ	HC = 18, SZ = 18, Total = 36	FC between 77 anatomical ROIs	RF	75%
A. Anderson and Cohen (2013)	SZ	HC = 29, SZ = 19, Total = 48	Graph measures	SVM	80%
Anticevic et al. (2013)	SZ/BP	HC = 90, SZ = 90, BP = 47, Total = 227	Thalamus seed‐based connectivity	SVM	73.90%
Arbabshirani et al. (2013)	SZ	HC = 28, SZ = 28, Total = 56	FNC between independent components extracted by ICA	Different linear and nonlinear methods	63–96%
Su et al. (2013)	SZ	HC = 32, SZ = 32, Total = 64	FC between 116 ROIs (AAL atlas)	SVM	82.80%
Yu, Shen, Zeng, et al. (2013)	SZ	HC = 38, SZ = 32, Total = 70	FC between 116 ROIs (AAL atlas)	SVM	80.90%
Yu, Shen, Zhang, et al. (2013)	SZ	HC = 25, HC sibling = 25, SZ = 24, Total = 74	FC between 116 ROIs (AAL atlas)	SVM	62.00%
Guo, Kendrick, Yu, Wang, and Feng (2014)	SZ	HC = 62, SZ = 69, Total = 131	FC between 90 ROIs (AAL atlas)	SVM	79–82%
Shen et al. (2014)	SZ	HC = 25, SZ = 24, Total = 49	Dynamic ALFF of ROIs	SVM	81.30%
Watanabe et al. (2014)	SZ	HC = 67, SZ = 54, Total = 121	FC between 347 spherical nodes	Lasso, elastic‐net, graph‐net and fused lasso	59.7–90.8%
H. Cheng, Newman, et al. (2015)	SZ	HC = 29, SZ = 19, Total = 48	Graph measures	SVM	80%
Du et al. (2015)	SZ/BP/Schizoaffective disorder	HC = 24, SZ = 24, BP = 24, SZA (manic) = 24, SZA (depressive) = 13, Total = 109	Network maps from ICA	SVM	68.75%
Kaufmann et al. (2015)	SZ	HC = 196, SZ = 71, Total = 267	FNC between independent components extracted by ICA	LDA	84.40%
Cabral et al. (2016)	SZ	HC = 74, SZ = 71, Total = 145	FC between 90 ROIs (AAL atlas)	SVM	70.50%
J. Kim et al. (2016)	SZ	HC = 50, SZ = 50, Total = 100	FC between 116 ROIs (AAL atlas)	DNN	86%
P Mikolas et al. (2016)	SZ	HC = 63, SZ = 63, Total = 126	Seed‐based FC	SVM	73%
Rashid et al. (2016)	SZ/BP	HC = 61, SZ = 60, BP = 38, Total = 159	Static FNC, dynamic FNC and combined static and dynamic FNC	SVM	59.12–88.68%
Cetin et al. (2016)	SZ	HC = 44, SZ = 47, Total = 91	Static FNC and dynamic FNC from fMRI and MEG data	LDC, NBC, and SVM	51.65–90.11%
Cetin et al. (2016)	SZ	HC = 348, SZ = 182, Total = 530	FNC between independent components extracted by ICA	Regularized LDA	69–78.3%
Y. Liu et al. (2017)	SZ	HC = 31, SZ = 48, Total = 79	Voxel‐mirrored homotopic connectivity	SVM	94.93%
J. S. Anderson et al. (2011)	ASD	HC = 40, ASD = 40, Total = 80	Whole brain FC between 7,266 ROIs	Statistic based classification score	79%
Murdaugh et al. (2012)	ASD	HC = 14, ASD = 13, Total = 27	FC between DMN ROIs	LR	96.30%
H. Wang, Chen, and Fushing (2012)	ASD	HC = 29, ASD = 29, Total = 58	FC between 106 ROIs (AAL atlas)	LR	82.80%
Deshpande et al. (2013)	ASD	HC = 15, ASD = 15, Total = 30	FC from fMRI and FA from DTI	SVM	95.90%
Nielsen et al. (2013)	ASD	HC = 517, ASD = 447, Total = 964	Whole brain FC between 7,266 ROIs	Statistic based classification score	60%
Uddin et al. (2013)	ASD	HC = 20, ASD = 20, Total = 40	Independent components extracted by ICA	LR	78–83%
Zhou et al. (2014)	ASD	HC = 153, ASD = 127, Total = 280	Graph measures	SVM and Bayesian network	70%
C. P. Chen et al. (2015)	ASD	HC = 126, ASD = 126, Total = 252	FC between 220 ROIs from meta‐analysis	RF	90.80%
Iidaka (2015)	ASD	HC = 328, ASD = 312, Total = 640	FC between 90 ROIs (AAL atlas)	Probabilistic neural network	90%
Plitt et al. (2015)	ASD	HC = 59, ASD = 59, Total = 118	Whole brain FC from different atlas	SVM	76.60%
H. Chen et al. (2016)	ASD	HC = 128, ASD = 112, Total = 240	Frequency distribution‐based FC	SVM	79.20%
Abraham et al. (2017)	ASD	HC = 468, ASD = 403, Total = 871	Whole brain FC from different atlas	SVM	68%
Jahedi, Nasamran, Faires, Fan, and Müller (2017)	ASD	HC = 126, ASD = 126, Total = 252	FC between 220 ROIs from the meta‐analysis of functional imaging studies	Conditional RF	71%
Ktena et al. (2018)	ASD	HC = 468, ASD = 403, Total = 871	Graph measures	CNN	80%
Sadeghi et al. (2017)	ASD	HC = 31, ASD = 28, Total = 59	Local and global functional network properties	SVM	92%
Bernas et al. (2018)	ASD	HC1 = 18, ASD1 = 12; HC2 = 12, ASD2 = 12, Total1 = 30, Total2 = 24	Time of in‐phase coherence between independent component extracted from ICA	LDA and SVM	86.70%
Heinsfeld, Franco, Craddock, Buchweitz, and Meneguzzi (2018)	ASD	HC = 530, ASD = 505, Total = 1,035	FC between 200 ROIs (CC200 ROI atlas)	Auto‐encoder	70%
C.‐Z. Zhu et al. (2008)	ADHD	HC = 12, ADHD = 12, Total = 24	ReHo	PCA‐FDA	85%
Bohland et al. (2012)	ADHD	168 subjects	FC from fMRI and features from sMRI and nonimage features	SVM	74%
Colby et al. (2012)	ADHD	HC = 491, ADHDI = 111, ADHDC = 163, ADHDH = 11, Total = 776	FC, graph measures, ReHo from fMRI and features from sMRI	SVM	55%
Dai et al. (2012)	ADHD	HC = 491, ADHD = 285, Total = 776	FC, ReHo from fMRI and cortical thickness, gray matter from sMRI	SVM	67.80%
Dey, Rao, and Shah (2012)	ADHD	HC,= 307, ADHD = 180, Total = 487	FC between ROIs	PCA‐LDA	65.60%
Sato et al. (2012)	ADHD	HC = 546, ADHD = 383, Total = 739	ALFF, ReHo and FC	SVM	67%
Fair et al. (2013)	ADHD	HC = 455, ADHD = 193, Total = 648	FC between 160 ROIs (Dosenbach atlas)	SVM	69.20%
Wang et al. (2013)	ADHD	HC = 23, ADHD = 23, Total = 46	ReHo	SVM	80%
Anderson et al. (2014)	ADHD	HC = 472, ADHD = 476, Total = 948	Graph measures from fMRI and features from sMRI	Decision tree	66.80%
Dey et al. (2014)	ADHD	HC = 307, ADHD = 180, Total = 487	Graph distance measures	SVM	73.55%
dos Santos Siqueira et al. (2014)	ADHD	HC = 340, ADHD = 269, Total = 609	Whole brain FC between 400 ROIs	SVM	77%
Deshpande et al. (2015)	ADHD	HC = 744, ADHDI = 173, ADHDC = 260, Total = 1,177	Directional and nondirectional whole brain FC	ANN	90%
Du, Wang, Jie, and Zhang (2016)	ADHD	HC = 98, ADHD = 118, Total = 216	FC between PCA selected regions	SVM	94.90%
Park et al. (2016)	ADHD	ADHDI = 13, ADHDC = 21, Total = 34	Whole brain FC between 384 ROIs during task	SVM	91.20%
Qureshi, Oh, Min, et al. (2017)	ADHD	HC = 67, ADHDI = 67, ADHDC = 67, Total = 201	FC from fMRI and features from sMRI	SVM	92.90%
Riaz et al. (2018)	ADHD	HC1 = 23, ADHD1 = 25; HC2 = 61, ADHD2 = 22; HC3 = 61, ADHD3 = 24; HC4 = 98, ADHD4 = 118, Total 1 = 48, Total 2 = 83, Total 3 = 85, Total 4 = 216	Whole brain FC between ROIs determined using the affinity propagation clustering algorithm and nonimage data	SVM	86.70%

**FIGURE 15:**
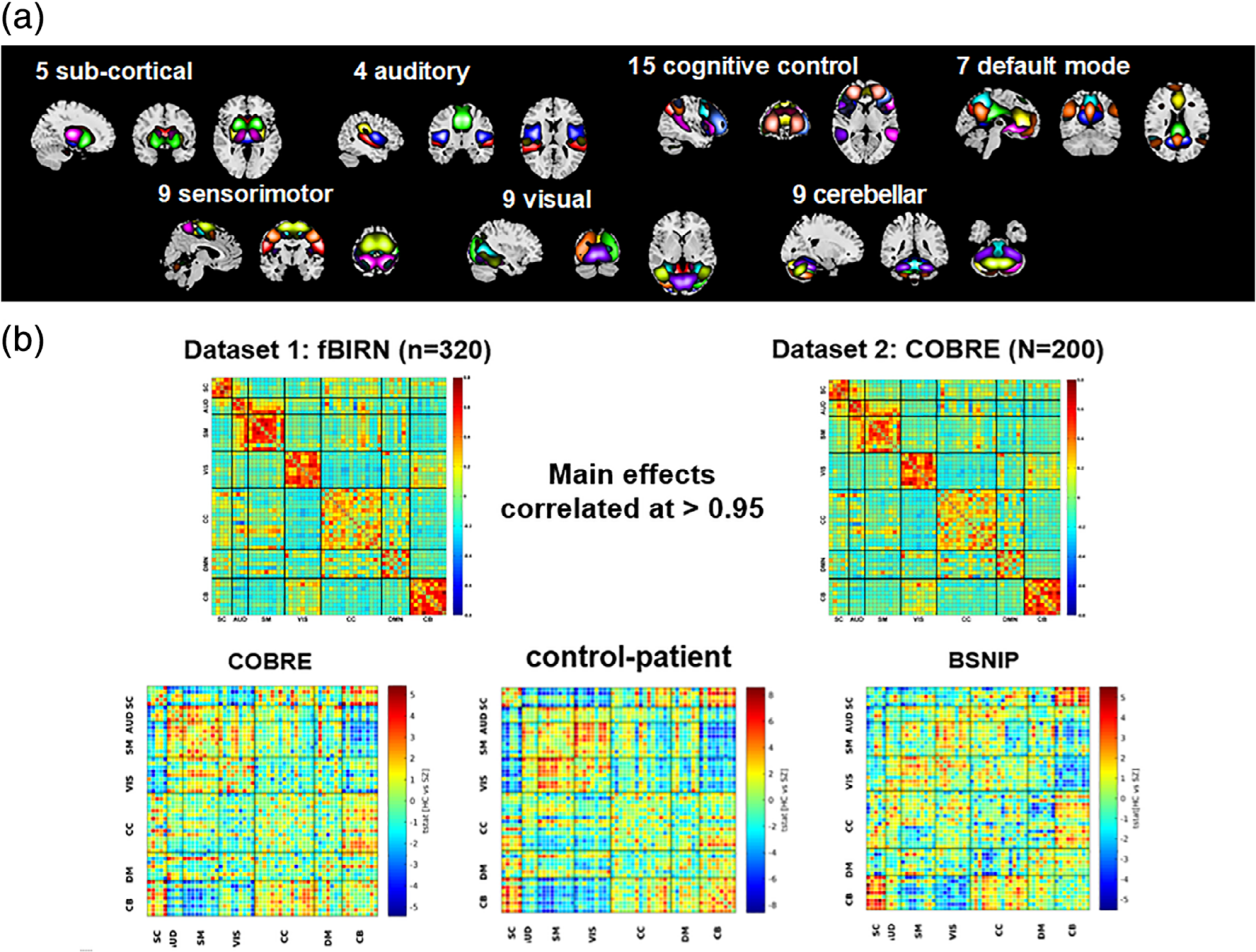
Illustration of the neuromark approach. The neuromark approach aims at linking neuromarkers among different diseases and separate studies. (a) The fully automated Group ICA components were found to be very stable which can be performed for individual subjects. (b) Strong correlation (>0.95) between functional network connectivity (FNC) was obtained across data from multiple sites, with consistent group difference between schizophrenia (SZ) and healthy cohorts. Figures reprinted with permission from Y. Du et al. (2019) and Lin et al. (2018)

FC measures have also been utilized as features for classification of autism and ADHD. Using resting‐state FC measures from 7,266 ROI unclosing only the gray matter, Anderson and colleagues achieved 89% accuracy for the subjects <20 years age and at 79% for all subject (J. S. Anderson et al., 2011). Murdaugh and colleagues used both seed‐based FC and whole‐brain FC in a logistic regression classifier to classify autism and reported 96.3% accuracy with both whole‐brain and seed‐based FC features (Murdaugh et al., 2012). Further, using FC between three ROI sets from ABIDE dataset, Plitt and colleagues applied RFE‐based feature selection and both logistic regression and SVM classifiers to classify autism and achieved an overall 76.7% accuracy (Plitt et al., 2015). In a multimodal classification study of autism, Deshpande and colleagues used FC estimates and fractional anisotropy (from DTI data) and obtained a maximum classification accuracy of 95.9% with a recursive cluster elimination based SVM classifier (Deshpande et al., 2013). Another recent resting‐state FC‐based (between 90 ROIs) prediction study used deep learning classifier (probabilistic neural network [PNN]) for classification of ASD and achieved a classification accuracy of about 90% (Iidaka, 2015). Interestingly, FC between signals in different frequency bands was used as features in a recent ASD classification study, where the Slow‐4 band (0.027–0.073 Hz) was found to capture the most discriminative features (H. Chen et al., 2016). To discriminate ADHD from healthy individuals, Zhu and colleagues used a PCA‐based Fisher discriminative analysis (PC‐FDA) with regional homogeneity (ReHo) from fMRI data as features, and showed a classification accuracy of 85% (C.‐Z. Zhu et al., 2008). Another study by Wang and colleagues also used ReHo from resting‐state fMRI data in a SVM classifier, and obtained a classification accuracy of 80% for discriminating ADHD from healthy individuals (Wang et al., 2013). Several other studies also used FC measures to successfully classify ADHD from HCs (Dey et al., 2014; D. Fair et al., 2013; João Ricardo Sato et al., 2012). Moreover, by leveraging a large‐scale resting‐state fMRI study of SZ from multiple sites (i.e., human connectome project), another recent study proposed a ICA‐based preprocessing pipeline to extract FNC and spatial map based imaging features as potential biomarkers. Results showed that compared to FNC‐based features, spatial map shows better classification performance in all experiments (Lin et al., 2019). Another study proposed a robust group information guided ICA (GIG‐ICA) based framework to estimate functional network maps and connectivity for linking neuromarkers among different diseases and separate studies, where the connectivity measures were independently computed and optimized to achieve independence based on each coming individual‐subject data. Findings from this study showed that the network features computed using the proposed method were more effective for predicting different diseases and classifying patients (Y. Du et al., 2019). One of the advantages of this framework is it does not require selecting components of interest and can be fully automated (Figure 15).

## 6 COMMON SHORTCOMINGS IN PREDICTOME STUDIES

### 6.1 Feature selection and reduction

An additional limitation of mental illness prediction studies includes the selection and dimensionality reduction of meaningful features. Almost all of the papers surveyed in this review have group‐level discriminative analysis followed by subject‐level classification. Many of these studies have first performed discriminative analyses using statistical tests (e.g., *t* tests) to extract significant features showing group difference, and then using these features performed subject‐level classification. However, the use of test dataset together with the training dataset during feature selection, extraction or reduction will introduce additional bias to the predictive model. This process of feature selection based on the group differences results identified from the whole sample could cause a “double dipping” issue, that may lead to a biased performance (Bishop, 2006; Demirci, Clark, Magnotta, et al., 2008). Another major issue with the group difference based feature selection approach is that the significance levels are based on *p* values of the statistical tests, which are not necessarily linearly associated with the discriminative power of the models. An alternative solution to the feature selection based on univariate group‐level statistical tests could be the use of filtering and wrapper methods (Blum & Langley, 1997; Hall & Smith, 1998). Filtering methods assign scores to each feature from which a number of top ones can be selected, while the wrapper methods consider selection of a set of feature as a search problem. Supervised feature selection methods have been most commonly used in the existing literature. However, since it has been reported that feature selection performance can be improved by increasing the sample size (Jain & Zongker, 1997), classification using a supervised feature selection algorithm on a small dataset might result in suboptimal performance. Further, unsupervised feature reduction methods, such as PCA have also been applied in the field of neuroimaging studies. As suggested by Osborne and Costello, unsupervised feature reduction methods on larger datasets could provide additional information for accurately generalizing population trends, which may lead to a more efficient model (Osborne & Costello, 2004).

### 6.2 Overfitting

Overfitting refers to a model that models the training data too well, resulting in very good classification performance on the training data (i.e., observed data) but very poor performance on independent, testing data (Pereira et al., 2009). Overfitting can be caused by utilizing models with large number of features from small sample size or complex models with many parameters, as the model would capture noisy features from the data more than the actual features of interest (Franke et al., 2010; Klöppel et al., 2008). Since neuroimaging datasets on mental illnesses have smaller samples in general and many features of interest, predictive models using these datasets are susceptible to overfitting. The majority of the surveyed studies reported in the current work performed predictive modeling based on a very small number of subjects, resulting in a decrease in the overall classification accuracy with smaller sample size. CV and regularization are common approaches to control for overfitting in neuroimaging data‐based predictive modeling.

### 6.3 Optimal model selection

In predictive modeling, model selection, more formally known as hyperparameter optimization or tuning, refers to the problem of choosing a set of optimal hyperparameters for a learning algorithm in order to achieve the best performance of the algorithm. The hyperparameter optimization step is performed during the training stage, typically during the CV of the training samples. One of the most commonly used classifiers is the SVM which is designed for binary classification that maximizes the boundary between classes in a high‐dimensional space. Linear SVM classifier includes a user‐defined soft margin hyperparameter that affects the trade‐off between errors on training data set and margin maximization. A smaller soft margin would introduce more errors, resulting in a larger margin. Nonlinear SVM classifier includes additional hyperparameters depending on the kernel of choice (e.g., sigma/gamma for RBF kernel and degree for polynomial kernel). Therefore, inefficient hyperparameter optimization could negatively influence model performance.

### 6.4 Challenge with reproducibility

In the existing brain‐based prediction literature, variability across raw data processing and analysis streams, feature types, feature selection schemes, choice of classifier, and CV methods may limit reproducibility of outcomes across independent datasets. Indeed, without any standard approach, the growing flexibility across machine learning pipelines is introducing less reliable replication across studies (Squeglia et al., 2016). In order to provide optimum diagnostic tools, the predictive models should be able to handle new samples obtained from independent research facilities. Further, the models should perform consistently across different data‐acquisition devices (e.g., Siemens/Philips/GE scanners), across different geographic areas (e.g., United States, Europe, etc.), and across diverse populations (e.g., same mental illnesses with distinctive comorbidity). Also, the performance should persist across different evaluation metrics (e.g., accuracy, sensitivity, specificity, etc.), as well as different medical settings (e.g., rural vs. city hospital; Woo, Chang, Lindquist, & Wager, 2017).

### 6.5 Prediction outcome comparison across studies

A common practice in prediction studies is to highlight the overall accuracy as the ultimate model performance measure. While many surveyed studies have reported that their algorithms performed better than other existing studies, this claim is often unsubstantiated and solely based on the comparison between their overall accuracy and that of the existing studies. Without well‐matched study variables (i.e., sample size, age, sex, scanner parameters and imaging sequences, medication, symptom scores, data modality, length of scanning for functional data, preprocessing pipeline, features of interest, feature selection methods, classifier type, and CV scheme), these comparisons are simply impractical. Further, any such performance‐related claims must be made after appropriate statistical significance tests.

### 6.6 Heterogeneity between patients

**FIGURE 16:**
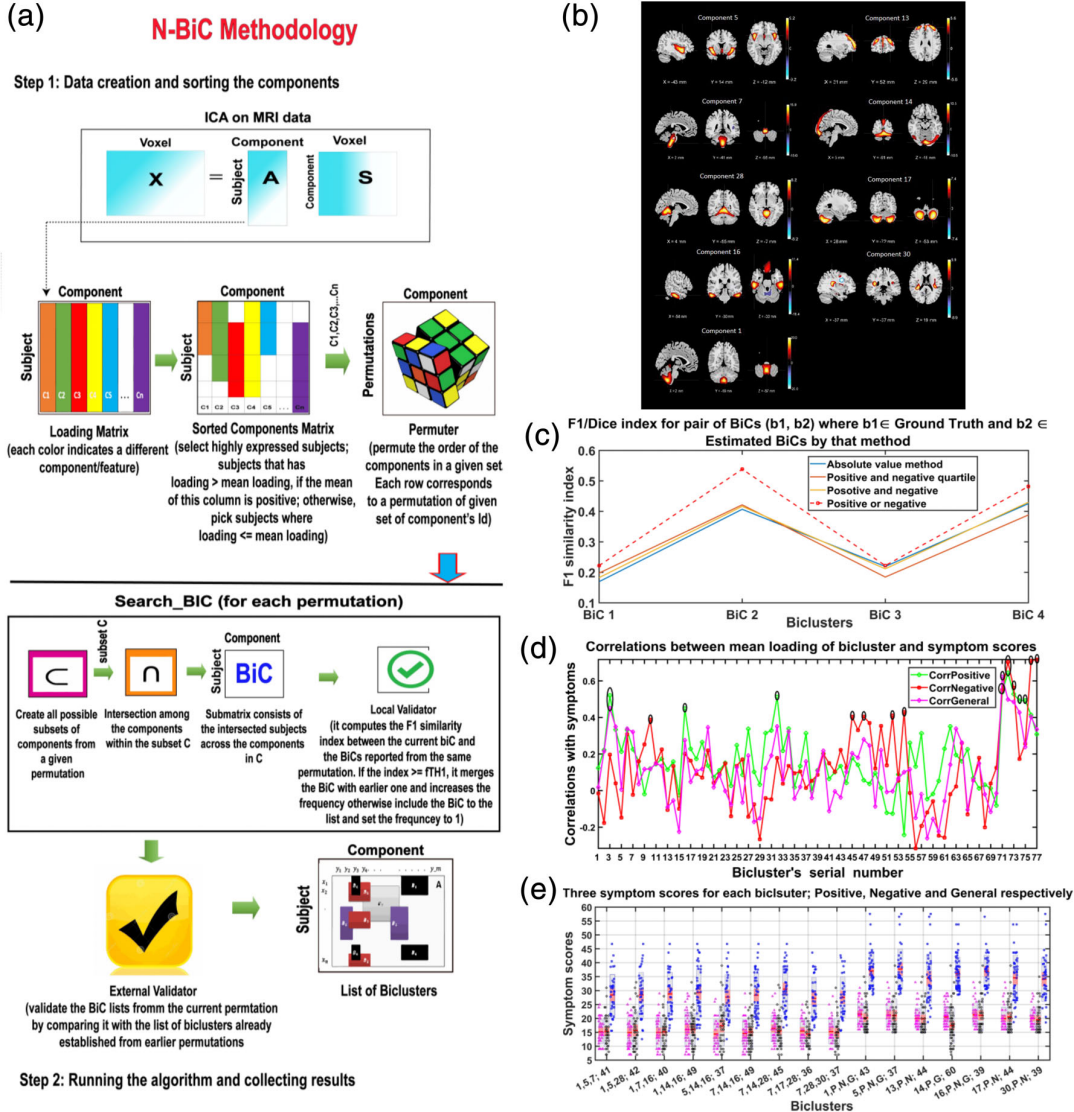
An approach for schizophrenia (SZ) prediction using multi‐component and symptom biclustering. (a) Block diagram of the general framework. (b) Discriminative components whose loading parameters were used as features. (c) F1 similarity index between estimated and ground truth biclusters, where a higher value indicates more similarity (better estimation). Red dotted line indicates method outperformed other methods. (d) Correlations between the symptom scores and biclusters. Colors represents different symptom scores; tall spikes indicate significant correlations. (e) Mean and standard deviation of symptom scores across biclusters. The dots represent the subject‐wise symptom score. Magenta: positive scores; black: negative scores; blue: general scores. Figures reprinted with permission from Rahaman, Turner, et al. (2019)

Another limitation for neuroimaging based machine learning studies is the substantial heterogeneity that exists between patients. In research‐based neuroimaging studies, participants are recruited based on well‐matched age, sex, or education background, typically with a particular type of brain pathology. In contrast, participants recruited in the clinical settings may include several types of pathology with variability in disease stage and demographic variables (i.e., age, sex etc.). As mentioned earlier, classification performance (e.g., accuracy) can be improved by using a larger training sample (Franke et al., 2010; Klöppel et al., 2008), and may also reduce the disease heterogeneity by integrating the whole spectrum of clinical and pathological features. Recent approaches, such as outlier detection (i.e., treating patient classification as an outlier detection problem (Mourão‐Miranda et al., 2011) can be implemented to reduce higher heterogeneity across patients. Further, two recent studies have performed multi‐component and symptom bi‐clustering where homogeneous clusters along various dimensions were identified for SZ subtype prediction (Gupta et al., 2017; Rahaman, Turner, et al., 2019; Figure 16).

## 7 FUTURE DIRECTIONS FOR BRAIN‐BASED PREDICTOME STUDIES

### 7.1 Prediction using deep learning techniques

In recent years, deep learning, which is a family of machine learning techniques, has gained significant attention for patterns recognition and classification in various data analysis fields including neuroimaging. The fundamental difference between traditional machine learning and deep learning is that, deep learning has the capability to detect and learn complex patterns from the raw data by utilizing consecutive nonlinear transformations. Due to its ability to identify complex and subtle (and potentially nonlinear) patterns based on data‐driven automatic feature learning process, deep learning has recently become an attractive tool in neuroimaging studies of mental illness. Deep learning‐based approaches are more effective when examining large number of features without any proper knowledge in feature selection. Using a hierarchical model of nonlinear layers, deep learning can model more complex data patterns. To summarize, the main advantages to use deep learning over traditional machine learning approaches include: (a) ability to implement data‐driven automatic feature learning and eliminating the subjectivity related to feature selection, and (b) the depth of model design that includes a hierarchy of nonlinear layers, allowing the model to efficiently identify complex data patterns.

Deep learning offers a promising tool for understanding the neural basis of mental illness by extracting complex, hidden patterns from high‐dimensional neuroimaging data (Kriegeskorte, 2015). Deep models have multiple advantages, including requiring exponentially smaller number of parameters to model the same thing as models by traditional machine learning approaches (Bengio, 2012). Further, for larger neuroimaging datasets, deep learning provides the opportunity to more efficiently diagnose mental illnesses (E. Castro, Ulloa, Plis, Turner, & Calhoun, 2015). In order to examine mental illness using deep learning, several studies have utilized a wide range of neuroimaging modalities including sMRI, resting fMRI, and multimodal data. For instance, using deep belief networks on both structural and functional MRI data, Plis and colleagues have demonstrated that deep learning methods are able to generate physiologically meaningful MRI‐based features that can uncover associations from high‐dimensional MRI data (Plis et al., 2014). Another study by Hjelm and colleagues used restricted Boltzmann machines (RBM) to capture intrinsic brain networks in fMRI data showed that RBMs could extract spatial networks and their activation with the accuracy of traditional matrix factorization methods such as ICA (Hjelm et al., 2014). Note that, RBM can be stacked to obtain deeper models as needed for deep learning which is a limitation in traditional approaches such as ICA, non‐negative matrix factorization (NMF), or sparse PCA. More recently, deep learning has been employed in classification of patients using neuroimaging data. Kim and colleagues used deep learning for classification of SZ patients from HCs based on functional connectivity patterns, and showed that their approach outperforms SVM by a significant margin (J. Kim et al., 2016). Using structural MRI data, Castro and colleagues developed a nonlinear independent component estimation (NICE) which is an extension of ICA using deep architecture, and detected gray matter abnormalities in patients with SZ. The resulting NICE components captured decreased patterns of GMC in SZ, showing potential for application in SZ prediction studies (Castro et al., 2016). Further, Jang and colleagues applied fully connected feed‐forward deep neural networks (DNN) to four sensorimotor task‐based fMRI data from 12 HC individuals. More specifically, the framework used a restricted Boltzmann machine‐based deep belief network classifier and obtained lower minimum error rates (mean ± *SD*; three‐layer DNN = 6.9 ± 3.8%, single‐layer DNN = 9.4 ± 4.6 and two‐layer DNN = 7.4 ± 4.1). Results showed that the DNN is able to classify a single‐volume fMRI extracted by hidden representations of task‐specific fMRI volumes distributed across multiple hidden layers, showing great potential for automatic mental illness prediction using fMRI volumes in cases where limited estimates of activation patterns or ad hoc statistical evaluation are available (Jang et al., 2017). Plis and colleagues implemented a deep learning method to identify association between multimodal neuroimaging data (i.e., sMRI and fMRI) in 144 SZ patients and 154 HC individuals. Dynamic FNC states and ICA‐based gray matter density maps were used as functional and structural features, respectively. Using the hidden association between brain structure and function, this study distinguished between SZ and HC participants (Plis et al., 2018). Another multimodal data integration study developed a neural network framework to study the brain development. Briefly, this study proposed a deep collaborative learning (DCL) method to investigated the difference of functional connectivity measures across different age groups, and achieved a maximum prediction accuracy of about 98% (W. Hu, Cai, Zhang, Calhoun, & Wang, 2019).

Within the field of neuroimaging, deep learning techniques have shown to achieve superior performance over many conventional machine learning approaches; however, currently there are some limitations and challenges of deep learning techniques which require further improvement prior to practical implementations. Some of the limitations of deep learning are discussed as follows:

1. Deep learning techniques are highly dependent on the quality and amount of training data which may result into overfitting (i.e., learning unrelated variations in the data), which may, in turn, result in lack of generalizability. Approaches such as regularization strategies (e.g., L1 or L2 norms, dropout, and weight decays) are being incorporated in deep learning methods in order to address the issue of overfitting (Hosseini‐Asl, Gimel'farb, & El‐Baz, 2016; J. Kim et al., 2016; F. Li, Huang, et al., 2014; S. Liu et al., 2014). The regularization strategies can improve the performance of the deep learning algorithms. Other approaches to avoid overfitting include dimensionality reduction (e.g., using region‐level features instead of voxel‐level features, selecting features using principal component analysis) of the data prior to training them using the model. Although, current evidence suggests that most deep learning models, including convolution neural network (CNN), can perform well with voxel‐level neuroimaging data without feature selection or reduction (Hosseini‐Asl et al., 2016).
2. Deep learning techniques require a large amount of training data to identify more generalized features and improved performance, and therefore demonstrate poorer performance with small sample size (Nie, Zhang, Adeli, Liu, & Shen, 2016). Further, noisy neuroimaging data may not improve the performance of the model beyond linear methods even with increasing sample size (e.g., ImageNet). One solution to this issue could be to use specific types of less‐noisy features to train the model. For example, in neuroimaging, voxel‐level features tend to be very detailed and noisy, whereas region‐level features show less sensitivity to noise as they overlook more localized patterns. Further, the accuracy of deep learning techniques may be improved by making full use of the automatically extracted features even for studies with smaller sample size.
3. Deep learning models offer a black box‐like system, which may introduce lack of transparency during the learning and testing steps (Alain & Bengio, 2016; Yosinski, Clune, Nguyen, Fuchs, & Lipson, 2015). In many cases, it is very difficult to understand the technical and logical bases of the model. The lack of transparency of deep learning may limit the interpretability of the neuroimaging results. Indeed, the multiple nonlinearities within deep models make it challenging to trace back the successive layers of weights to the original brain data, therefore limiting the ability to detect abnormalities within brain regions (Suk et al., 2015). To address this issue, currently two approaches are being implemented: (a) input modification methods (i.e., visualization techniques by systematically modifying the input, the resulting changes in the output, and in intermediate layers; example: occlusion method; Zeiler & Fergus, 2014) and (b) deconvolution methods (i.e., determining the contribution of features of the input to the output data; example: guided back‐propagation; Springenberg, Dosovitskiy, Brox, & Riedmiller, 2014).
4. Another challenging issue for deep learning methods is the workflow integration, particularly for clinical use of these models. In order to implement these deep models in the clinical settings, it is necessary to share the relevant knowledge of these models with the clinicians, as well as receive feedback from them as the end‐users during the model development stage.

### 7.2 Large‐scale/big data approaches

The most commonly observed limitation in the field of mental illness predictive studies is the limited sample size. The lack of larger datasets (i.e., number of subjects) as well as insufficient phenotypic details, such as clinical record, comorbidities, symptom and disease progression, treatment outcomes, and responses limit the scope of utilizing machine learning algorithm for developing personalized care of mental illness. The majority of the prediction studies in this review have a relatively small sample size. Although sample size is a common limitation across all prediction studies, compared to the other fields of studies in which machine learning is used, neuroimaging studies in general have very small sample size due to the difficulties of recruiting patients and the cost of collecting the data and the data also have high dimensionality. This may introduce several issues including decrease in classifier performance (Franke et al., 2010; Klöppel et al., 2009), lack of generalization for diagnosis purposes, and inability to address disease heterogeneity, and model overfitting due to poor sample size (Pereira et al., 2009). For optimal evaluation of machine learning methods, therefore larger sample size is required to minimize the variance in assessments of accuracy, sensitivity, specificity, and other performance measures.

To address this limitation within neuroimaging research, multiple ongoing efforts have created dataset repositories. The large‐scale or “big data” revolution shows promises to reduce data heterogeneity related issues in neuroimaging studies (Franke et al., 2010; Klöppel et al., 2008). While most existing neuroimaging studies consist of moderate sample sizes, typically less than 50 subjects, a few number of studies have started to embrace the “big data” for neuroimaging by either collecting tens of thousands of subjects or taking advantage of huge increases in the quantity of imaging and nonimaging features collected on each subject. Currently, there exist three types of big‐data initiatives: (a) centralized, (b) decentralized data sharing repositories, and (c) large‐scale studies.

#### 7.2.1 Centralized repositories

Centralized data sharing repositories offer open sharing of data. Many of these repositories include multiple sites and commercial cloud computing setups to provide support with high computational load. Some examples of centralized repositories (there are many dozens of these currently) include:

1. *NIMH data archive*: The Adolescent Brain Cognitive Development Study (ABCD) (Casey et al., 2018).
2. *NITRC*: For SZ, ASD, and ADHD (Buccigrossi et al., 2008).
3. COINS: Collaborative Informatics and Neuroimaging Suite or COINS for ASD patients (https://coins.mrn.org/).

Note that, these are only some examples of what have been shared vie the centralized repository platforms.

#### 7.2.2 Decentralized repositories

Additionally, to address the legal, ethical, and sociological concerns that might prohibit open data sharing initiatives and avoid re‐identification of study subjects, repositories with anonymized raw data are also being established.

1. *COINSTAC*: a decentralized and privacy‐enabled infrastructure model for brain imaging data (Gazula et al., 2018; Plis et al., 2016).

Further information on implementation of decentralized algorithms, enhancement of user interface, regression statistic calculation for decentralization, and comprehensive pipeline specifications can be found in Ming et al. (2017).

#### 7.2.3 Large‐scale studies

Moreover, several multi‐site studies have also started to share neuroimaging data in a collaborative setup. These studies include:

1. *FBIRN*: For SZ patients (J. M. Ford et al., 2008).
2. *COBRE*: For SZ patients (center of biomedical research excellence, http://cobre.mrn.org/).
3. *MCIC*: For SZ patients (Gollub et al., 2013).
4. *Functional Connectomes Project*: For healthy subjects (B. B. Biswal et al., 2010).
5. *ABIDE*: For ASD patients (Di Martino et al., 2014).
6. *ADHD‐200*: For children with ADHD (Consortium & others, 2012).

While the data sharing initiatives show potential (Milham et al., 2018), there are several methodological challenges of big data approaches in the field of neuroimaging which need to be addressed.

1. The lack of uniformity in data acquisition and processing across contributing sites could introduce performance bias of predictive modeling while using the pooled multi‐site data. For large‐scale imaging studies, there are several potential artifacts of concern which may include factors affecting imaging and other variables of interest, such as (a) head motion, (b) head size, (c) heart rate and respiratory variations, (d) variability in scanner hardware, and (e) variability in scanner hardware. To mitigate the effects of these confounding factors, more sophisticated image processing techniques should be developed (for examples of such processing techniques, see (1) (Salimi‐Khorshidi et al., 2014); rsfMRI data, (2) (Andersson & Sotiropoulos, 2016); dMRI data, and (3) (Fortin et al., 2017; Fortin et al., 2018; Johnson, Li, & Rabinovic, 2007); dMRI and sMRI data. Further efforts are required to standardize data acquisition parameters across all data sharing sites for more ideal data pooling. For example, when sMRI data were analyzed, inconsistency in field strength and imaging sequence design showed evidence of significant systematic differences in multi‐site studies (Fennema‐Notestine et al., 2007; Stonnington et al., 2008). Without standardized parameter agreement, the variability observed across the subjects is increasingly driven by variability in scanner and imaging parameters, which could potentially introduce false disease‐specific effects. Another study showed similar field related differences how in 10,000 subjects across 1.5 T and 3 T scanners, they also showed evidence of highly consistent changes associated with age (Panta et al., 2016). By designing and maintaining study protocols across all contribution sites, and developing analytic approaches which are more robust to site effects (e.g., end‐to‐end deep learning to predict and remove site effects), chances of observing disease‐specific effects are increased. The benefits of big data are many, and with improved integration between participating sites, in term of acquisition and other parameters, the potential inhomogeneities can be mitigated.
2. Another major issue for “big” imaging data is the statistical challenge. These rich datasets are designed to explore a variety of hypotheses. As researchers investigate multiple imaging modalities, many of them tend to explore various alternative models to search for significance without proper multiple comparison testing or CV framework. This makes CV and replication even more essential. Similarly, the effect size should also be reported, if using null hypothesis testing, big data can provide highly significant results for tiny effect sizes, which may not be particularly useful for any individual subjects. Also, the use of robust test statistics is important, for example, the use of, nonparametric testing, such as permutation‐based tests can be incorporated while examining multiple modalities (Winkler et al., 2016).
3. With big data comes the “curse of dimensionality”. Compared to the number of observations, there are many features in high dimensional data, making it susceptible to issues such as sparsity, multicolinearity, computational cost, model complexity, and overfitting. One potential solution could be to implement feature selection or reduction approaches, such as principal component analysis, prior to analyzing and modeling the data.
4. Neuroimaging data sharing through the big data consortiums have raised some ethical and privacy concerns, for example, the possibility of facial reconstruction from structural images. By removing recognizable facial features using the de‐facing approach prior to data sharing, this concern can potentially be addressed. Other ethical concerns include the risk of subject identification based on their geographical location, since typically these large‐scale studies are conducted within a particular region. By adopting multilayer, restricted data sharing approach, more controlled access to the full dataset can be achieved, thus eliminating the risk of subject identification. Another approach is to utilize federated learning or decentralized analysis approaches, for example, the COINSTAC tool allows one to perform regression as well as more advanced voxel‐wise and machine learning‐based approaches in a decentralized framework without requiring the data to be shared (Plis et al., 2016).

### 7.3 Standard machine learning competitions in neuroimaging

In the field of machine learning, recent standard predictive analyses‐based competitions have already contributed to the growth of technologies. Typical layouts of such competitions include: (a) provide the participants with a labeled training dataset and an unlabeled testing dataset, (b) based on the training dataset, the participants attempt to develop a predictive model with best performance, and then apply the trained model to predict the testing samples’ labels, and (c) finally, the participants submit the prediction results and performance. Using the standard training and testing dataset and some basic preprocessing provided by these competitions, the participants can focus on the predictive modeling aspects without any biased outcomes. Although, in neuroimaging, such competitions are not as common as other fields due to the restricted data sharing policies, a number of brain‐based machine learning competitions have been held in recent years. In 2011, the ADHD‐200 competition was held which included resting‐state fMRI data, as well as anatomical and phonotypical data of 776 subjects (491 typically developing children and 285 ADHD) for training purposes and 197 subjects as testing dataset (Consortium & others, 2012), with the goal of distinguishing between patients with ADHD and healthy, typically developing children. This competition demonstrated a greater prospect toward multi‐site, large‐scale ADHD data sharing. Another more recent machine learning competition was organized by the IEEE MLSP workshop that provided only neuroimaging‐based features, with the goal of automatic prediction of SZ patients from HCs (Silva et al., 2014). Briefly, the participants were provided with FNC measures from resting‐state fMRI, as well as the ICA loadings of SBM measures from structural MRI from 144 subjects (75 HCs and 69 SZ patients). The competition included 245 participating teams, with the winning team obtaining an AUC of around 0.90. Additionally, an AUC of around 0.94 was achieved by combining the top three models (Silva et al., 2014). Further, in 2018, to challenge participants in predicting MDD and HCs using structural MRI data, the predictive analytics competition (PAC) was arranged (https://www.photon-ai.com/pac). The competition included training data from 759 MDD patients and 1,033 HCs and unlabeled testing data from 448 subjects obtained across three different publicly available sites. The winners achieved a classification accuracy of 65%. These standard machine learning competitions show the potential for brain‐based mental illness predictions, as they are able to evaluate data with accurate, unbiased predictive power.

### 7.4 Benefits of leveraging dynamic connectivity features

Until recently, functional connectivity has been assumed to be relatively stable over the scanning time (usually several minutes). While convenient for analysis and interpretation purposes, this over‐simplified assumption was recently challenged by several studies focused on time‐varying multivariate connectivity patterns (Sakoğlu et al., 2010), as well as in studies focusing on time‐frequency analysis methods (C. Chang & Glover, 2010). Several other studies have also delved into the time‐resolved connectivity measures and their successful applications in identifying biomarkers using dynamic connectivity features (Allen et al., 2014; Calhoun, Miller, Pearlson, & Adalı, 2014; Du et al., 2016; Rashid et al., 2018; Rashid, Damaraju, Pearlson, & Calhoun, 2014; Zalesky & Breakspear, 2015). These studies reported that brain functional connectivity can vary within a short period (e.g., tens of seconds), and can successfully capture the connectivity disruptions in a disease population.

Only a few studies have utilized dynamic brain connectivity features to predict mental illness. Using static and dynamic connectivity features, Rashid and colleagues developed a classification framework to predict SZ, bipolar, and healthy subjects (Rashid et al., 2016). The classification performance measures among static, dynamic and combined static and dynamic connectivity features were compared using a 10‐fold CV framework. The results showed that dynamic FNC (classification accuracy: 84.28%) significantly outperformed the static FNC (classification accuracy: 59.12%), suggesting that dynamic patterns in functional connectivity might provide distinct and more information over the static FNC. Another study used a novel recurrent neural network (RNN) approach (i.e., a family of deep learning) to measure temporal dynamics and dependencies among brain networks (X.‐H. Wang, Jiao, & Li, 2018). Using SVM classifier and LOO/10‐fold CV, another study classified ADHD children using data from the ADHD‐200 Consortium. Specifically, the temporal variability between intrinsic connectivity networks and the demographic and covariate variables were utilized as features. The LOOCV achieved total accuracy of 78.75, while the 10‐folds CVs achieved average prediction accuracy of 75.54% ± 1.34, showing evidence of accurate prediction of ADHD using temporal dynamics and SVM classifier. Further, Demirtas and colleagues used SVM classifier and global spatiotemporal measures, static FNC and variability in FC, and reported that the combined static FC and variability in FC provided a classification accuracy of 81% (sensitivity: 81%; specificity: 81%) for classification of MDD (Demirtaş et al., 2016).

### 7.5 Fusion of dynamic connectivity and other data types

**FIGURE 17:**
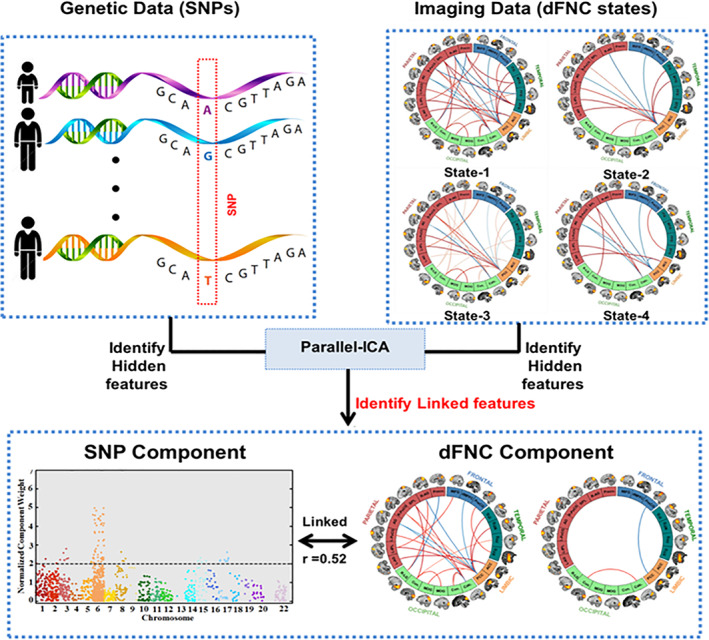
An imaging‐genomics framework to jointly estimate group differences in schizophrenia (SZ) and healthy controls (HC). The parallel ICA based multimodal framework incorporating imaging (dynamic FNC measures) and genomic (single nucleotide polymorphism, SNP) data. Figures reprinted with permission from Rashid et al. (2019)

Dynamic FNC measures estimated from fMRI data can be further integrated with other data types and modalities, for example, genomic and structural MRI data, to leverage inter‐modality based features for disease characterization. In a novel imaging‐genomic framework, Rashid and colleagues have recently modeled the association between dynamic FNC states and genomic features to examine the SZ‐related inter‐modality abnormalities (Rashid et al., 2019; Figure 17). Specifically, the parallel ICA algorithm (J. Liu et al., 2009)was utilized to combine genetic variants (i.e., single nucleotide polymorphism [SNP]) and functional features from fMRI data as subject‐specific states that are revealed from the dynamic FNC data using a sliding window and clustering approach (Allen et al., 2014; Rashid et al., 2014) to distinguish SZ from healthy individuals. Results identified a significant association between a SNP component (defined by large clusters of functionally related SNPs statistically correlated with phenotype components) and dynamic FNC component (defined by clusters of related connectivity links among distant brain regions distributed across discrete dynamic states, and statistically correlated with genomic components) in SZ. Moreover, the polygenetic risk score (PRS) for SZ (computed as a linearly weighted sum of the genotype profiles with weights derived from the odds ratios of the psychiatric genomics consortium [PGC]) showed negative correlation with the significant dynamic FNC component, which were mostly present within a state that exhibited a lower occupancy rate in individuals with SZ compared with HC, therefore identifying a potential dynamic FNC imaging biomarker for SZ. Another recent study by Abrol and colleagues proposed an mCCA+jICA framework to fuse dynamic FNC from fMRI data and gray matter maps from structural MRI data (Abrol, Rashid, Rachakonda, Damaraju, & Calhoun, 2017). The framework identified the associated changes in both modalities, highlighting significantly disrupted links between dynamic FNC and gray matter volumes in SZ patients. Results from this study reported significant group differences in gray matter maps, particularly in the superior parietal lobule, precuneus, postcentral gyrus, medial/superior frontal gyrus, superior/middle temporal gyrus, insula and fusiform gyrus. Further, results also highlighted alterations in several inter‐regional connectivity strength in SZ patients. In the field of brain‐based prediction of mental illness, fusion approaches using dFNC and other features could increase the discriminative power of the models, although future studies are required to confirm their utilities in this regard.

## 8 SUMMARY AND CONCLUSION

Recent brain‐based mental illness predictome studies have shown promising results, although some results require further validation due to small sample sizes. While many advanced algorithms have been developed and applied in the field of mental illness prediction, there exists many challenging issues which must be further resolved prior to their applications in clinical settings. In this work, we comprehensively review existing brain‐based prediction studies in several mental illnesses such as SZ, depressive disorders (i.e., MDD and BP), ASD, ADHD, SAD, OCD, PTSD, and substance disorder. We also highlight a number of existing approaches and future research directions. A major challenge in the field is the prediction of phenotypic heterogeneity that characterizes psychiatric disorders. However, recent approaches have started to address the disease subtypes. This can improve disease prediction, provide biological support for existing categories or support the revision of existing diagnostic categories. Another major challenge is the relatively small sample size reported across most studies. Without more robust validation, it is unclear how generalizable these results will be when applied to an independent dataset. However, recent data‐sharing initiatives have started to improve the sample size issue by offering adequate data to develop more robust and improved prediction models.

While brain‐based classification has proven challenging, there has been considerable progress made in recent years. With the accelerating growth of large volumes of patient data and data‐sharing initiatives in the field of neuroimaging and medicine, we anticipate diagnostic tools operating on comprehensive biomarker profile accessed from multiple modalities will be available for specific use cases in the near future. With more sophisticated deep learning models integrated with large‐scale data, we believe that predictive modeling tools will soon transition from the “proof‐of‐concept” stage to the “ready for clinical implementation” stage. Further, while patient characteristics appear to be more homogeneous within relatively small samples (Schnack & Kahn, 2016), failing to capture disease‐specific variability, with the power of “big” brain data and advanced machine learning algorithms, it is now possible to explore the heterogeneity within a disease (i.e., disease sub‐types). It is a common practice for clinicians to consider diagnostic homogeneity (i.e., patients with similar clinical symptoms belong to the same broader diagnostic category). We can also evaluate this within imaging data using, for example, N‐way clustering approaches to identify subgroups of homogeneous data followed by evaluation of clinical phenotypes. One recent example shows this in SZ and finds enhanced sensitivity to group differences and stronger links to symptoms scales that is typically found (Rahaman, Damaraju, & Calhoun, 2019; Rahaman, Turner, et al., 2019). By identifying complex and heterogeneous brain‐based disease patterns, predictive modeling can potentially be used clinically for more personalized medicine targeted at specific subtypes or clusters of the disorder with varying symptomology and disease progression. However, this can only be achieved by integrating clinical and technical expertise, possible by some back‐and‐forth feedback system between both fields’ experts, until the tools are optimized as well as simplified for clinical applications. Finally, we expect the brain‐based predictome to progress beyond the categorical diagnosis (i.e., identifying disease groups), and taking into account some of the key continuous measures, such as cognition and behavior, to provide a comprehensive diagnostic approach. We look forward to seeing the full potential of the brain‐based predictome realized.

There are several limitations to this work. In this survey, we restricted our search to MRI‐based, English journal articles only for specific mental disorders. Further, we did not focus on other modality‐based mental illness prediction studies, such as EEG, MEG, and PET. Also, we narrowed our focus on mental disorders only and did not consider other brain disorders such as Alzheimer's disorder, mild cognitive impairment, and Parkinson disease. Moreover, we mostly reported the best performing features and classifiers, and experimental setups.

## CONFLICT OF INTEREST

The authors declare no conflict of interest related to this work.

## Data Availability

Data sharing is not applicable to this article as no new data were created or analyzed in this study.
